# Hamiltonian description of non-reciprocal interactions

**DOI:** 10.1038/s41567-026-03317-0

**Published:** 2026-06-12

**Authors:** Yu-Bo Shi, Roderich Moessner, Ricard Alert, Marin Bukov

**Affiliations:** 1 Max Planck Institute for the Physics of Complex Systems, Dresden, Germany; 2 School of Physics, Nankai University, Tianjin, China; 3 Max Planck Institute for the Physics of Complex Systems and Cluster of Excellence ctd.qmat, Dresden, Germany; 4 Center for Systems Biology Dresden, Dresden, Germany; 5 Cluster of Excellence Physics of Life, TU Dresden, Dresden, Germany; 6 Departament de Física de la Matèria Condensada, Universitat de Barcelona, Barcelona, Spain; 7 Universitat de Barcelona Institute of Complex Systems, Barcelona, Spain; 8 Institució Catalana de Recerca i Estudis Avançats (ICREA), Barcelona, Spain

**Keywords:** Physics, Condensed-matter physics, Statistical physics, thermodynamics and nonlinear dynamics

## Abstract

In many systems, including sedimenting particles and bird flocks, interactions do not derive from a potential and are generally non-reciprocal, meaning that they do not obey the action–reaction principle. As a result, one cannot define a conventional energy function or use analytical and numerical tools that rely on it. Here we address this limitation by constructing a Hamiltonian with auxiliary degrees of freedom that, under a constraint, generates the original non-reciprocal dynamics. We show that Monte Carlo simulations based on the constrained Hamiltonian reproduce both stationary and non-stationary states of the original Langevin dynamics, as we illustrate for dissipative *XY* spins with vision-cone interactions. The symplectic structure inherent to the construction also lets us apply established ideas from Hamiltonian engineering, which we demonstrate by varying the amplitude of a periodic (Floquet) drive to tune the spin interactions between square- and chain-lattice geometries. Overall, our construction paves the way towards extending statistical mechanics and Hamiltonian dynamics to non-reciprocal systems.

## Abstract (web summary)

Many active and driven systems exhibit non-reciprocal interactions, making them hard to describe with standard methods. Now a constrained Hamiltonian embedding with auxiliary variables reproduces these dynamics in model spin systems.

## Main

Newton’s third law states that every action has a reaction equal in magnitude and opposite in direction. For two particles interacting via a potential, the interaction forces are reciprocal: **F**_2→1_ = −**F**_1→2_. However, for many mesoscopic and macroscopic particles, interactions do not arise from a potential but are instead mediated by a non-equilibrium field^1,2^. For example, interactions between colloids can be mediated by hydrodynamic flows, by a dissolved chemical, or by a temperature field^3–8^. Respectively, interactions between animals, such as birds, wildebeest or humans, can be mediated by their vision field^9–11^. In such systems, interactions are in general non-reciprocal: **F**_2→1_ ≠ −**F**_1→2_. Non-reciprocal interactions have been reported in systems including sedimenting particles^2,12–14^, spinning starfish embryos^15^, and active colloids and droplets^16–24^; they have also been programmed in colloids^25,26^ and in robots^27–29^. Despite recent advances^29–35^, a general framework of statistical mechanics for non-reciprocal systems is still missing^30^.

**Fig. 1:**
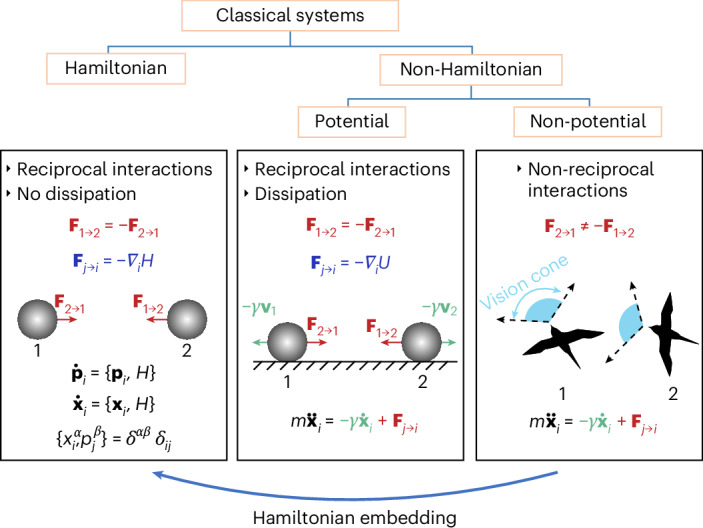
Classification of classical systems. Classical systems can be classified as either Hamiltonian (for example, two electric charges in vacuum, left) or non-Hamiltonian. The latter can have interactions that may be derived from a potential (for example, two electric charges on a frictional substrate, middle) or not (for example, birds with vision-cone interactions, right). In this work, we construct a Hamiltonian embedding for non-reciprocal systems.

To appreciate the reason for this, note that, in classical and statistical mechanics, one may distinguish between Hamiltonian and non-Hamiltonian systems (Fig. 1). Hamiltonian systems possess conjugate pairs of variables that span phase space (for example, position and momentum). Mathematically, this is expressed by the Poisson bracket {⋅, ⋅} which endows phase space with a so-called symplectic structure^36^. This enables the use of canonical transformations^37,38^, which preserve the Poisson bracket, to find integrals of motion or derive simplified effective models that approximate the dynamics^38–40^.

Non-Hamiltonian systems, meanwhile, lack conjugate variables, and hence do not enjoy any of the advantages offered by the symplectic structure. Broadly speaking, they fall into two categories: systems whose equations of motion are governed by conservative forces that can be derived from a potential, and those that cannot (Fig. 1). A simple example of a non-Hamiltonian system with interactions arising from a potential is a pair of particles subject to dissipation, for example through friction with a substrate (Fig. 1, middle). Here, we define dissipation by lack of conservation of phase-space volume under the dynamics. A notable advantage of having a potential is the ability to use Monte-Carlo methods^41^ to investigate the properties of the system coupled to a thermal bath.

Conversely, in the simplest setting, non-reciprocal systems are modelled by forces that cannot be derived from a single mutual interaction potential^30^. These non-conservative forces do not admit an energy function and are, furthermore, non-Hamiltonian in general. Physically, this means that non-reciprocal systems are intrinsically out of thermal equilibrium, which substantially complicates their study beyond the bare simulation of the underlying equations of motion. For instance, due to the absence of a potential, it is a priori unclear how to apply Monte-Carlo methods to simulate and analyse properties of their nonequilibrium steady states. Similarly, due to the lack of conjugate variables, deriving effective descriptions for non-reciprocal models cannot be done using canonical transformations.

**Fig. 2:**
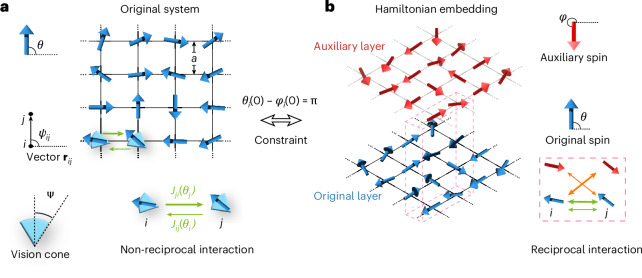
Schematic illustration of Hamiltonian embedding. **a**, Schematic illustration of *XY* spins with vision-cone interactions on a square lattice. Each spin (blue arrow) described by the angle *θ*_*j*_ interacts only with nearest neighbors within its vision cone of angle Ψ (cyan); a pair of unidirectional thin/thick green arrows show the non-reciprocal interaction strength. The vector from site *i* to site *j* is denoted by ${{\bf{r}}}_{ij}=a(\cos {\psi }_{ij},\sin {\psi }_{ij})$. **b**, Corresponding Hamiltonian embedding. A layer of auxiliary spins *φ*_*j*_ (red arrows) is coupled to the original spins (blue) reciprocally (see inset and equation (2)). Imposing the constraint that auxiliary spins mirror the original ones, *θ*_*i*_(0) − *φ*_*i*_(0) = π, we recover the equations of motion for the non-reciprocal system from the equations of motion of its Hamiltonian embedding.

In this work, we remove these obstacles by designing a Hamiltonian embedding for non-reciprocal systems. Specifically, for any equation of motion with pairwise interactions described by non-reciprocal forces, we present a construction of a reciprocal Hamiltonian, composed of the original system and an auxiliary degree of freedom for each original one (Fig. 2). By imposing a constraint on each (original, auxiliary) pair, the equations of motion derived from this Hamiltonian reproduce the original non-reciprocal dynamics. Our results apply equally to inertial and dissipative non-reciprocal dynamics; for dissipative non-reciprocal systems in particular, the original and auxiliary degrees of freedom form canonically conjugate variables.

We exemplify our construction using dissipative *XY* spins on a square lattice with vision-cone and chase-and-run interactions—paradigmatic examples of non-reciprocal interactions^33,42–44^. To demonstrate the utility of our construction, we first show (both analytically and numerically) the equivalence of the states, either steady or non-stationary, obtained from Monte-Carlo simulations with Glauber dynamics based on the Hamiltonian embedding on the one hand, and the original equations of motion subject to Langevin noise on the other. We then use the emergent symplectic structure of the Hamiltonian embedding to analyse the vision-cone *XY* system subject to a periodic drive using Floquet theory^45^, and derive effective equations of motion for the driven system. In the Supplementary Information, we provide a general proof for the construction, accompanied by applications to five experimental systems featuring non-reciprocal interactions: metal-dielectric Janus particles^19,24^, walker robots^46^, robotic metamaterials^27^, feedback-controlled active particles^25^ and sedimenting particles^12^.

## *XY* spins with non-reciprocal vision-cone interactions

Consider a square lattice of linear dimension *L* and lattice constant *a* with periodic boundary conditions. Each lattice site *i* contains a classical spin whose orientation, ${{\bf{S}}}_{i}=(\cos {\theta }_{i},\sin {\theta }_{i})$, can rotate continuously within the lattice plane, and is parametrized by the angle *θ*_*i*_. The spins interact via pairwise vision-cone interactions of strength

$${J}_{ij}({\theta }_{i})=\left\{\begin{array}{cc}J, & \,\mathrm{for}\,\,\min \left\{2{\rm{\pi }}-\left|{\theta }_{i}-{\psi }_{ij}\right|,\left|{\theta }_{i}-{\psi }_{ij}\right|\right\}\le \varPsi \\ 0, & \mathrm{else},\end{array}\right.,$$

where Ψ denotes half the vision cone angle, and *ψ*_*ij*_ is the angle between the line connecting sites *i* and *j* and the horizontal (Fig. 2a, inset). The reciprocal *XY* model is recovered for Ψ = π. We couple the system to an external bath of temperature *T* through a Gaussian (white) noise term *η*_*i*_(*t*) with zero mean and unit variance; this bath defines the notion of temperature used throughout this work. The interacting spins undergo Langevin dynamics, following the equation of motion

1 $${\dot{\theta}}_{i}(t)=-\mathop{\sum}\limits_{j \in \{\langle ij \rangle \}}{J}_{ij}({\theta}_{i})\sin ({\theta}_{i}-{\theta}_{j})+\sqrt{2T}{\eta}_{i}(t),$$

where the Boltzmann constant *k*_B_ = 1, and the damping coefficient *γ* = 1. The corresponding vision-cone *XY* interaction (Fig. 2a) gives rise to a minimal model for non-reciprocal interactions, which has recently gained significant attention. Previous work has studied the role of non-reciprocity on the transition from the disordered to the ferromagnetic phase^33,42–44^, as well as on the creation and annihilation of topological defects^43,47^.

Because a spin may be in the vision cone of its neighbour, but not the other way around (Fig. 2a), vision-cone interactions are non-reciprocal: *J*_*ij*_(*θ*_*i*_) ≠ *J*_*ji*_(*θ*_*j*_); hence, the magnitudes of the torques acting on spins *i* and *j*, as given by the right-hand side of equation (1), differ. Thus, one cannot assign an unambiguous interaction energy to each lattice bond (Fig. 2a, inset), and therefore vision-cone interactions cannot be derived from a potential.

## Constrained Hamiltonian embedding

We now present a procedure that enables us to obtain non-reciprocal interactions in the equations of motion from an effective Hamiltonian function, in the absence of the noise term *η*_*i*_. This is achieved by first extending the model by adding an auxiliary set of degrees of freedom, that is, doubling the configuration space. Introducing suitable couplings between original and auxiliary degrees of freedom using reciprocal interactions allows us to construct a well-defined Hamiltonian. The original non-reciprocal dynamics is generated from Hamilton’s equations of motion for states obeying one constraint on each pair of (original, auxiliary) degrees of freedom, as explained below. This constraint reduces the size of the configuration space back to that of the original non-reciprocal system. Crucially, the constraint is preserved by the dynamics, so that it suffices to impose it once, for example on the initial state, of the time evolution.

Let us demonstrate the procedure using the vision-cone dynamics from equation (1), see the Supplementary Information for more examples. We introduce a set of auxiliary spins, ${{\bf{a}}}_{i}=(\cos {\varphi }_{i},\sin {\varphi }_{i})$, on an auxiliary lattice layer (Fig. 2b, red spins), which point opposite to the spins in the original system: **a**_*i*_ = − **S**_*i*_. To grasp the essence of the idea, consider first two neighbouring spins **S**_*i*_, **S**_*j*_ which interact non-reciprocally. The key insight is to symmetrize the non-reciprocal interaction between **S**_*i*_ and **S**_*j*_, but then use the interaction with the neighbouring auxiliary spins **a**_*i*_, **a**_*j*_ to each cancel one part of the symmetric coupling between the **S** spins (Fig. 2b, inset). Note that this cancellation works only if the auxiliary spins are antiparallel to the original, **a**_*i*_ = −**S**_*i*_. This is the origin of the constraint that we need to enforce, which we call the mirror constraint. There is no interaction between the auxiliary spins **a**_*i*_.

Following this procedure, we write a Hamiltonian

2 $$H({\theta }_{i},{\varphi }_{i})={H}_{SS}({\theta }_{i})+{H}_{Sa}({\theta }_{i},{\varphi }_{i}),$$

with conjugate degrees of freedom {*θ*_*i*_, *φ*_*j*_} = *δ*_*ij*_, where {⋅, ⋅} is the Poisson bracket; hence, *φ*_*i*_ acts as the canonical momentum for *θ*_*i*_, see the Supplementary Information for details.

The first term, *H*_*SS*_, describes the symmetrized interactions within the original system,

3 $${H}_{SS}=-\mathop{\sum }\limits_{\langle ij\rangle }\left[{J}_{\!ij}({\theta }_{i}) + {J}_{\!ji}({\theta }_{j})\right]\cos ({\theta }_{i}-{\theta }_{j}).$$

Here, the energy of bond 〈*ij*〉 is given by the sum of the vision-cone interactions along that bond, ${J}_{\left\langle ij\right\rangle }\left({\theta }_{i},{\theta }_{j}\right)={J}_{ij}({\theta }_{i})+{J}_{ji}({\theta }_{j})$. Although this symmetrized interaction is reciprocal, it depends on the orientations *θ*_*i*_ and *θ*_*j*_.

The second term, *H*_*Sa*_, represents the coupling between the two layers, where the auxiliary spins **a**_*i*_ interact with the original spins **S**_*j*_, following

$${H}_{Sa}=\mathop{\sum }\limits_{\langle ij\rangle }\left[-{J}_{ij}({\theta }_{i})\cos ({\theta }_{j}-{\varphi }_{i})-{J}_{\!ji}({\theta }_{j})\cos ({\theta }_{i}-{\varphi }_{j})\right].$$

The magnitude of each of the two terms in *H*_*Sa*_ above directly corresponds to that in the symmetrized interaction (Fig. 2b, inset); as we shown next, this is essential to recover the original non-reciprocal dynamics.

The equations of motion generated by the Hamiltonian (equation (2)), ${\dot{\theta}}_{i}=\left\{{\theta }_{i},H\right\}$ and ${\dot{\varphi}}_{i}=\left\{{\varphi }_{i},H\right\}$, form a set of equations that couple the auxiliary and original degrees of freedom:

4 $$\begin{array}{rcl}{\dot{\theta}}_{i}(t) & = & {\partial}_{{\varphi}_{i}}H=\mathop{\sum}\limits_{j \in \{\langle ij\rangle \}}{J}_{ij}\left({\theta }_{i}\right)\sin \left({\varphi }_{i}-{\theta }_{j}\right),\\ {\dot{\varphi}}_{i}\left(t\right) & = & -{\partial }_{{\theta }_{i}}H=\mathop{\sum }\limits_{j\in \{\langle ij\rangle \}}-{J}_{ij}({\theta }_{i})\sin ({\theta }_{i}-{\theta }_{j})\\ & & -{J}_{\!ji}({\theta }_{j})\left[\sin ({\theta }_{i}-{\varphi }_{j})+\sin ({\theta }_{i}-{\theta }_{j})\right]\\ & & +{\partial }_{{\theta }_{i}}\,{J}_{ij}({\theta}_{i})\left[\cos ({\theta }_{j}-{\varphi }_{i})+\cos ({\theta }_{j}-{\theta }_{i})\right].\end{array}$$

The ${\partial }_{{\theta }_{i}}{J}_{ij}({\theta }_{i})$ term appears because the coupling strength *J*_*ij*_(*θ*_*i*_) depends on the spin degree of freedom *θ*_*i*_; this implicit dependence is at the origin of the discrepancy between the dynamics generated by the reciprocal textbook *XY* model with the replacement *J*_*ij*_ ↦ *J*_*ij*_(*θ*_*i*_) and the dynamics of equation (1)^47^.

If the mirror constraint is imposed on the initial conditions,

5 $${{\bf{a}}}_{i}(0)=-{{\bf{S}}}_{i}(0)\,\,\iff \,\,{\theta }_{i}(0)-{\varphi }_{i}(0)={\rm{\pi }},$$

it is preserved under the equations of motion, that is, *θ*_*i*_(*t*) − *φ*_*i*_(*t*) = π for all times, as we show in the Supplementary Information. Because, under the constraint, the dynamics of the *φ*_*i*_(*t*) degree of freedom is tied to that of the *θ*_*i*_(*t*) spins, the equations of motion (equation (4)) uncouple. Moreover, each degree of freedom recovers precisely the non-reciprocal vision-cone interaction term from equation (1). Formally, the constraint (equation (5)) causes the unwanted terms in the last two lines of equation (4) to cancel, and makes the remaining equations identical to one another. Notice that, after applying the constraint, the vision cones for *φ*_*i*_ and *θ*_*i*_ point in the same directions, even though *θ*_*i*_ − *φ*_*i*_ = π.

The procedure illustrated above is not limited to vision-cone *XY* interactions. In the Supplementary Information, we first present a general proof for arbitrary non-reciprocal interactions. To illustrate the wide applicability of our framework, we construct the Hamiltonian for five specific examples: metal-dielectric Janus particles^19,24^, self-aligning active particles such as walker robots^46,48^, robotic metamaterials^27^, feedback-controlled active particles^25^ and sedimenting particles^12^. In these examples, the dynamics are overdamped, that is, dissipation dominates over inertia. However, using suitable kinetic-energy terms, the Hamiltonian embedding also applies to inertial (that is, non-dissipative) non-reciprocal dynamics, suggesting a broad applicability (Supplementary Information).

According to Liouville’s theorem, without constraint, the Hamiltonian embedding gives rise to non-dissipative dynamics in the embedding phase space, even if the original equations of motion are dissipative. This feature is brought about by the symplectic structure inherent to the Hamiltonian embedding (for example, in the vision-cone *XY* spins that we discussed, *θ* and *φ* are conjugate variables). By contrast, on the submanifold defined by the constraint, the dynamics are dissipative. This apparent contradiction is resolved by noticing that the constrained submanifold is a set of measure zero in the embedding phase space that does not contribute to phase-space volume.

As a direct consequence of the constraint, trajectories with initial conditions on the constraint manifold remain confined to it for all time, and hence never intersect with trajectories that start outside. Therefore, dynamics outside the manifold, which are reciprocal, do not affect the dynamics on the constraint manifold, which are non-reciprocal. Hence, the phase-space probability distribution evolves independently on the constraint manifold and outside it. It is therefore possible for an initial ensemble on the constraint manifold to evade thermalization, even when the embedding Hamiltonian dynamics outside the manifold are ergodic. This provides a way to embed non-equilibrium configuration-space distributions of non-reciprocal systems into the larger Hamiltonian phase space. Because the non-reciprocal manifold is a set of measure zero within the total phase space, it does not affect expectation values of observables defined on the total phase space. Hence, observables on the embedding phase space are insensitive to non-stationary behaviours on the non-reciprocal manifold, such as the breaking of continuous time-translation symmetry or persistent configuration-space currents.

Furthermore, notice that, when evaluated on the constraint (equation (5)), the total Hamiltonian *H* vanishes identically; therefore, *H* does not describe the energy of the original non-reciprocal system, but merely provides a generator for its dynamics. Moreover, the ground-state manifold of *H* need not obey the constraint. These unusual features reflect the inherent out-of-equilibrium character of the original non-reciprocal system.

Finally, we note that the form of the Hamiltonian embedding is not unique. For instance, one can distribute the interaction symmetrically among the two types of degrees of freedom without changing the resulting equations of motion upon imposing the constraint. It is currently an open question whether this freedom can be used to imprint additional properties or constraints into the embedding Hamiltonian.

In what follows, we illustrate the utility of the proposed Hamiltonian description by (1) demonstrating that the long-time states of the original Langevin dynamics (equation (1)), either steady or nonstationary, coincide with those of Glauber dynamics generated by the Hamiltonian (equation (2)), thus enabling the use of Monte-Carlo methods to study non-reciprocal dynamics. In addition, (2) we engineer the strength of vision cone interactions along different lattice directions with the help of high-frequency periodic drives, explicitly using the symplectic structure of the Hamiltonian formulation; this enables the application of Floquet engineering to control the phase transitions of non-reciprocal systems.

## Equivalence between Hamiltonian Monte-Carlo and Langevin dynamics subject to white noise

The absence of a well-defined interaction energy per bond precludes the direct application of Monte-Carlo methods to study non-reciprocal systems. To circumvent this problem, recent works have introduced new concepts and constructions^49–51^ to perform Monte-Carlo simulations, such as the ‘selfish energy’ of a spin on the lattice site *i*, ${E}_{i}=-{\sum }_{j\in \langle ij\rangle }\,{J}_{ij}({\theta }_{i})\cos ({\theta }_{i}-{\theta }_{j})$ (refs. ^33,34,52^), or modified acceptance rates that include non-reciprocal interactions through a Legendre-like transform similar to a grand-canonical ensemble^53^. A notable common disadvantage of these tools is that it is a priori unclear whether the resulting steady states correspond to those of the microscopic Langevin dynamics; moreover, many non-reciprocal systems evolve into non-stationary states. In addition, these methods do not enjoy formal mathematical guarantees for convergence, unlike Monte-Carlo methods for reciprocal systems^41^.

Starting from the reciprocal Hamiltonian embedding, we propose a Monte-Carlo algorithm to sample non-equilibrium steady states and nonstationary states of non-reciprocal systems. Specifically, we apply constrained Glauber dynamics^54^ at temperature *T* defined by the transition rates $w\left({\theta }_{i}\to {\theta }_{i}^{{\prime} }\right)=1/2\left[1-\tanh (\Delta E({\theta }_{i},{\theta }_{i}^{{\prime} })/(2T))\right]$ with

6 $$\Delta E\left({\theta }_{i},{\theta }_{i}^{{\prime} }\right)=\frac{1}{2}{\left.\left\{H({\theta }^{{\prime} },\varphi )-H(\theta ,{\varphi }^{{\prime} })\right\}\right|}_{{\rm{constr.}}}\,.$$

Here, the notation {⋅}∣_constr._ means that the operations in the bracket are carried out first, and only then is the constraint applied. As a result, the auxiliary variable *φ*_*i*_ drops out of the expression for the energy difference $\Delta E({\theta }_{i},{\theta }_{i}^{{\prime} })$. Consequently, we only need to simulate the dynamics of the original **S**-spin layer, thus keeping the number of simulated degrees of freedom equal to that of the original system.

For large system sizes, *L* ≫ 1, these Glauber dynamics give rise to a Fokker–Planck equation for the configuration-space distribution $\rho ({\boldsymbol{\theta }},{t}_{{\rm{MC}}})$ (Methods):

7 $$\frac{\partial \rho (\boldsymbol{\theta },{t}_{{\rm{MC}}})}{\partial {t}_{{\rm{MC}}}}=\alpha \mathop{\sum }\limits_{i=1}^{{L}^{2}}{\partial }_{{\theta }_{i}}\left[T{\partial }_{{\theta }_{i}}\rho (\boldsymbol{\theta },{t}_{{\rm{MC}}})-{F}_{i}(\boldsymbol{\theta })\rho (\boldsymbol{\theta },{t}_{{\rm{MC}}})\right],$$

where ${F}_{i}(\boldsymbol{\theta })=-{\partial }_{{\theta }_{i}}H{\left.(\boldsymbol{\theta },{\bf{\varphi }})\right|}_{{\rm{constr.}}}$ is the (non-reciprocal) force acting on spin **S**_*i*_. Here, *α* is a scaling factor relating the physical time and Monte-Carlo time: *t* = *αt*_MC_, set by temperature *T* and the support size Δ of the uniform distribution according to which the Glauber updates are performed (Methods).

In the Methods, we prove that equation (7) coincides with the Fokker–Planck equation obtained from the original Langevin equations of motion. Therefore, the Hamiltonian embedding allows us to define transition rates that enable the use of the Glauber algorithm in non-reciprocal systems. Our Monte-Carlo scheme allows us to sample nonequilibrium states since detailed balance is broken by the constraint (equation (6); Supplementary Information). Hence, we can bypass brute-force Langevin dynamics and directly sample both non-equilibrium steady states as well as oscillatory non-stationary states, as we now demonstrate in numerical simulations.

### Non-equilibrium steady states

For the vision-cone spins from equation (1), using the Hamiltonian embedding (equation (2)) we compute the energy difference entering the constrained Glauber transition rates as $$\Delta E({\theta }_{i},{\theta }_{i}^{{\prime} })=-\frac{1}{2}{\sum }_{j\in \langle ij\rangle }[{J}_{ij}({\theta }_{i}^{{\prime} })+{J}_{ij}({\theta }_{i})]$$$$\times [\cos ({\theta }_{i}^{{\prime} }-{\theta }_{j})-\cos ({\theta }_{i}-{\theta }_{j})]$$.

**Fig. 3:**
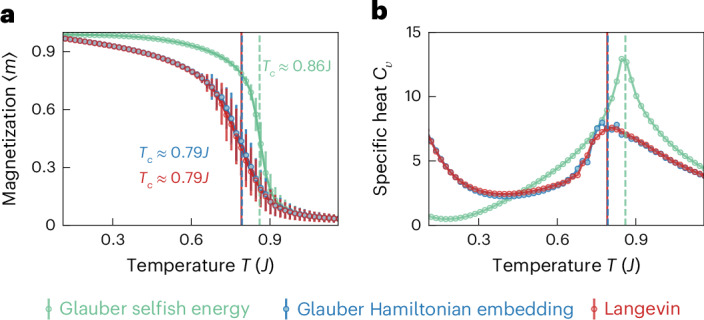
Non-equilibrium steady state in *XY* spins with vision-cone interactions. **a**,**b**, Average magnetization 〈*m*〉 with standard deviation *σ*_*m*_ (error bars) (**a**), and specific heat *C*_*v*_ (**b**) as functions of temperature *T*, obtained from Monte-Carlo simulations with Glauber dynamics based on either the selfish energy (green) or the constrained Hamiltonian embedding (blue), as well as simulations of the original Langevin dynamics (red). Whereas the constrained Glauber and Langevin simulations show the same critical temperature within the statistical uncertainty: *T*_*c*_/*J* = 0.79 ± 0.04 (Langevin) and *T*_*c*_/*J* = 0.79 ± 0.04 (constrained Glauber), the selfish-energy Glauber dynamics predicts *T*_*c*_/*J* = 0.86 ± 0.02*J*. The square lattice has linear dimension *L* = 100, and the vision cone angle is Ψ = 0.85π. For the Langevin dynamics, we set a timestep *Jδt* = 0.01 and use 100*L*^2^ iterations. In the constrained Glauber algorithm, we set a support size Δ = 0.1, and use 200*L*^4^ iterations. A total of *n*_eq_ = 5 × 10^4^ data points are uniformly sampled from the last 50*L*^2^ (50*L*^4^) iterations for Langevin (constrained Glauber) dynamics of ${\mathcal{N}}=100$ independent trajectories, each initialized with a different random seed, at each temperature. The total number of samples is therefore $n={n}_{\mathrm{eq}}\times {\mathcal{N}}=5\times 1{0}^{6}$. For selfish-energy Glauber dynamics, *n*_eq_ = 10^4^ and ${\mathcal{N}}=100$ (ref. ^76^).

In Fig. 3, we compare (a) the average magnetization $\langle m\rangle =\langle \sqrt{{({\sum }_{i}\cos {\theta }_{i})}^{2}+{\left({\sum }_{i}\sin {\theta }_{i}\right)}^{2}}/{L}^{2}\rangle$ and (b) the specific heat, ${C}_{v}=\left(\left\langle {H}_{SS}^{2}\right\rangle -{\left\langle {H}_{SS}\right\rangle }^{2}\right)/{\left(LT\right)}^{2}$, obtained as the variance of the energy in the original spin layer^55^, as a function of temperature, for constrained Glauber dynamics (blue) versus Langevin dynamics (green). We initialize the system in an ordered state, *θ*_*i*_ = 0, with initial energy of the original system *H*_*SS*_ = 3*L*^2^. Consistent with previous works^33,42–44^, our data indicate the existence of a phase transition between an ordered phase at low temperature and a disordered phase at high temperature (Fig. 3a). Accordingly, the specific heat *C*_*v*_ exhibits a peak at the phase transition point (Fig. 3b). Importantly, both the Langevin and the constrained Monte-Carlo simulations exhibit the same critical temperature, *T*_*c*_/*J* = 0.79 ± 0.04, for the phase transition, within statistical uncertainty (Supplementary Information). By contrast, a Monte-Carlo simulation using the Hamiltonian (3) without the effect of the auxiliary spins **a**_*i*_ produces a critical temperature of *T*_*c*_/*J* ≈ 1.70 (refs. ^56^) (not shown).

Unlike Glauber dynamics based on the selfish energy^33^^,34^, our constrained-Hamiltonian Monte-Carlo update rule results in the same configuration-space density for the non-equilibrium steady state as the Langevin equation. Indeed, the critical temperatures for the phase transition estimated from the Langevin and the selfish-energy approach do not coincide in general (compare green and red curves in Fig. 3), although for some parameters the values may agree within statistical uncertainty. By ensuring the equivalence of the Langevin and the constrained Monte-Carlo dynamics, our Hamiltonian embedding resolves this long-standing issue in the study of vision-cone interactions^47^.

### Non-stationary states

We now show that the Hamiltonian embedding also describes non-reciprocal systems that exhibit long-time non-stationary states, such as sustained oscillations, corresponding to the breaking of time-translation symmetry.

**Fig. 4:**
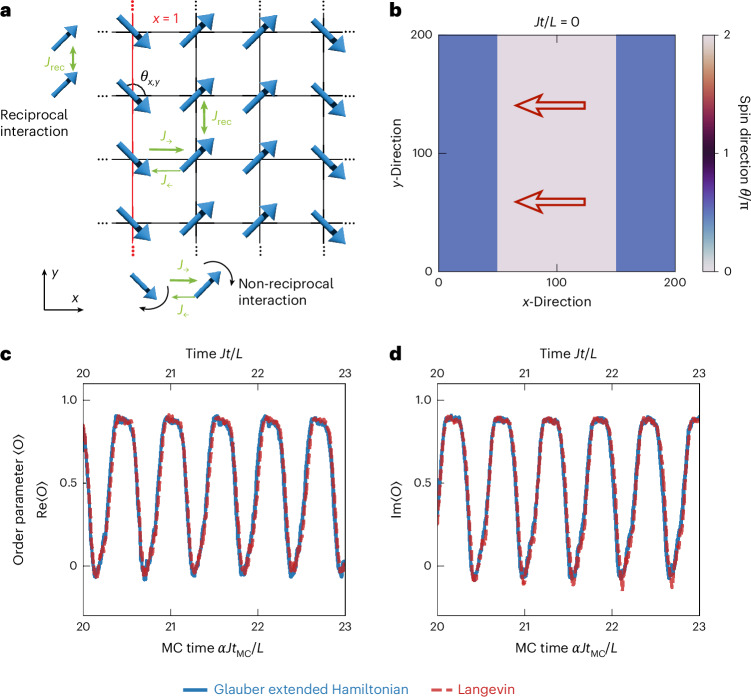
Non-stationary state of *XY* spins with chase-and-run interactions. **a**, *XY* spins on a square lattice with non-reciprocal interactions along the *x*-direction, and reciprocal interactions along the *y*-direction. The *x* = 1 column used to define the order parameter *O*(*t*) is marked in red. **b**, Stripe pattern used as an initial steady state, which moves perpetually to the left due to the non-reciprocal interactions (Supplementary Video 1). **c**,**d**, Long-time evolution of the real (**c**) and imaginary (**d**) part of the stripe order parameter 〈*O*(*t*)〉 averaged over an ensemble of ${\mathcal{N}}=100$ independent realizations of the noisy dynamics. The data show excellent agreement between the Langevin (dashed line, top horizontal axis) and the constrained Glauber simulations (solid line, bottom horizontal axis) with transition rates obtained from the Hamiltonian embedding. The time axis is rescaled by the linear system size *L*. The parameters are *J*_→_/*J* = 1, *J*_←_/*J* = − 0.99, *J*_rec_/*J* = 10, *T*/*J* = 0.08, Δ = 0.05 and *L* = 200, with the scaling factor *α*(Δ, *T*) = 2.37 × 10^−3^ extracted numerically.

Consider the dynamics of *XY* spins on a square lattice following equation (1), but with non-reciprocal interactions *J*_←_ ≠ *J*_→_ along the *x*-direction (independent of the spin orientation), and reciprocal interactions *J*_rec_ along the *y*-direction (Fig. 4a). The initial state is the vertical stripe pattern shown in Fig. 4b. Whenever *J*_←_ ≈ − *J*_→_, the spins exhibit a chase-and-run dynamics^29^ and the pattern moves to the left (for *J*_→_ > 0) at a constant speed (Supplementary Video 1). In the thermodynamic limit, this non-stationary state propagates perpetually due to an interplay between noise and many-body interactions, breaking continuous time-translation symmetry. These sustained dynamics do not have an equilibrium analogue, and hence present an ideal testbed for the validity of our constrained Glauber dynamics.

Following the procedure in the ‘Constrained Hamiltonian embedding’ section, we calculate a Hamiltonian embedding for this model (Methods); then, using equation (6), we find the expression for the energy difference $\Delta E(\theta ,{\theta }^{{\prime} })$ (equation (27)).

Figure 4c,d shows the dynamics of the complex-valued order parameter $O(t)={L}^{-1}{\sum }_{y=1}^{L}{{\rm{e}}}^{i{\theta }_{1,y}(t)},$ which characterizes the average orientation of the spins within the *x* = 1 column of the lattice (Fig. 4a, red). Then, with periodic boundary conditions, the real and imaginary parts of 〈*O*(*t*)〉 exhibit temporal oscillations as a result of the collective spin rotation at the interface between the stripes, which moves due to the non-reciprocal interactions. As anticipated from our analytical theory, we find an excellent agreement between the Langevin and Monte-Carlo dynamics at all times, including transients. This provides numerical evidence that Glauber dynamics with transition rates determined from the Hamiltonian embedding capture correctly the non-stationary-state behaviour of non-reciprocal systems.

We emphasize that, while above we used two specific models to demonstrate the equivalence between Monte-Carlo dynamics and the original non-reciprocal Langevin equation, the Hamiltonian embedding applies to generic non-reciprocal systems, as we show in the Supplementary Information.

## Hamiltonian engineering for non-reciprocal systems

Whereas the Glauber dynamics in the previous section rely on the embedding Hamiltonian to define transition rates, they do not make use of the underlying symplectic (or phase-space) structure, as Monte-Carlo simulations only require an energy function. The symplectic structure of the embedding Hamiltonian enables the use of canonical transformations to analyse the physics of non-reciprocal systems, for example, in identifying sets of strongly and weakly interacting degrees of freedom that define fast and slow variables, or in determining conserved quantities. Its existence is also inherently related to the existence of a Hamiltonian as a generator of dynamics.

**Fig. 5:**
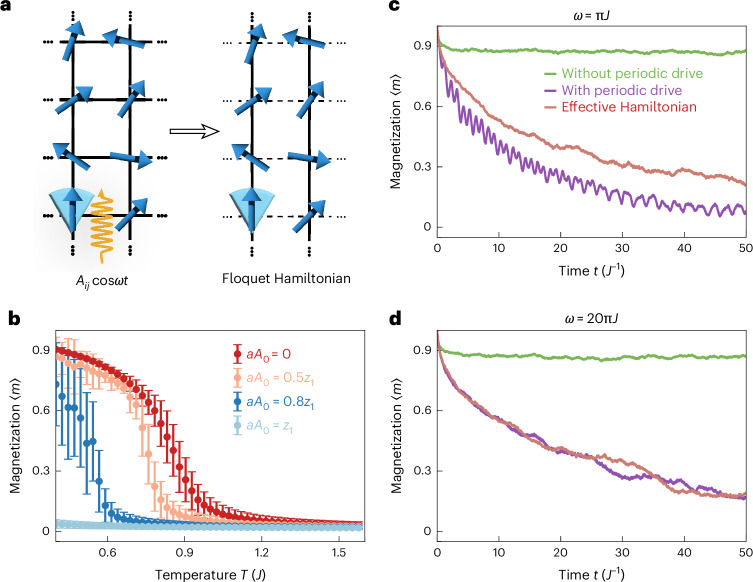
Floquet engineering of non-reciprocal *XY* spins. The Hamiltonian embedding enables the use of Floquet techniques to analyse driven non-reciprocal interacting systems. **a**, Schematic illustration of the periodically driven *XY* spins (left, equation (9)) and the effective time-averaged interactions (right, equation (10)). Thin dashed black lines indicate suppression of interactions in the *x*-direction. **b**, Average magnetization 〈*m*〉 and standard deviation *σ*_*m*_ (error bars) obtained from Langevin dynamics as a function of temperature *T* for different drive amplitude strengths *A*_0_. As *A*_0_ increases, the transition temperature decreases; as predicted by the time-averaged Floquet Hamiltonian, when $A_0=z_{\alpha} a^{-1},$ where $z_{\alpha}$ is a zero of the $\mathcal{J}_0$ Bessel function (see text), the system no longer exhibits a long-range ordered phase. We use 100*L*^2^ iterations with a timestep of *Jδt* = 0.01; *ω* = 20π*J* and *Ψ* = 7π/8. A total of *n*_eq_ = 10^4^ data points are uniformly sampled from the last 50*L*^2^ timesteps of each of ${\mathcal{N}}=100$ independent trajectories, each initialized with a different random seed, at every temperature. The total number of samples used to compute averages and standard error bars is therefore $n={n}_{{\rm{eq}}}\times {\mathcal{N}}=1{0}^{6}$ (ref. ^76^). **c**,**d**, Average magnetization 〈*m*〉 obtained from Langevin dynamics as a function of time *t* for two drive frequencies: *ω* = π*J* (**c**) and *ω* = 20π*J* (**d**), for *T* = 0.5*J*. As the frequency increases, the dynamics corresponding to the equations of motion derived from ${H}_{F}^{(0)}$ (orange) become closer to the exact periodically driven Langevin dynamics (purple). Both show a significant deviation from the Langevin dynamics of the non-driven system from equation (1) (green). Parameters: *A*_0_ = 2*a*^−1^, and *L* = 64. No ensemble averaging is performed here.

As a concrete example of leveraging the symplectic structure provided by the embedding Hamiltonian, here we demonstrate the application of Hamiltonian engineering to modify the properties of non-reciprocal systems using high-frequency periodic driving^45,57,58^. To this end, we consider a hypothetical experiment on a system of *XY* spins interacting non-reciprocally on a square lattice (Fig. 5a). Hamiltonian engineering allows one to tune the interaction strength independently along the different lattice directions, which may enable one to explore regimes not accessible otherwise. As an example, below we show how to use high-frequency periodic driving to modify the interactions and achieve a dimensional crossover, whereby a square lattice of spins with vision-cone interactions is effectively turned into a one-dimensional chain (Fig. 5a).

To define the drive, note that the *XY* model arises naturally in the description of Josephson junction arrays; when subject to an external a.c. magnetic field, the field can be modelled by a time-dependent vector potential *A*_*ij*_ which couples to the phase difference *θ*_*i*_ − *θ*_*j*_ of the superconducting order parameter^59^. While the discussion here is not bound to a specific experimental system, this setting illustrates concretely how a periodic drive can modify the interaction term when it is not possible to directly tune its coupling strength *J*_*ij*_. In the presence of the drive, the equations of motion read as

8 $${\dot{\theta}}_{i}(t)= - \mathop{\sum}\limits_{j \in \{\langle ij \rangle \}}{J}_{ij}({\theta}_{i})\sin ({\theta}_{i}-{\theta}_{j}-{A}_{ij}\cos \omega t)+\sqrt{2T}{\eta}_{i}(t).$$

We model the external periodic drive along the *x*-direction with frequency *ω* and amplitude ${A}_{ij}=a{A}_{0}\cos {\psi }_{ij}$, where the angle ψ_*ij*_ is defined in Fig. 2a, and *a* is the lattice constant^60–62^.

We now demonstrate that the periodic drive enables us to effectively suppress the spin interactions along the *x*-direction only (Fig. 5a) while keeping the vision-cone interaction along the *y*-direction—an instance of Floquet engineering. In the literature on quantum systems^45,57,58^, this phenomenon is known as dynamical localization, because an external drive is used to suppress the transfer of spin-state population between neighbouring lattice sites, controlled by the corresponding spin–spin interaction strength. In the case of non-reciprocal interactions, the periodic drive leads to weakening and eventually complete decoupling along the *x*-bonds of the lattice. In systems with limited control over the interaction strength, this strategy would allow experiments to study the fate of the phase transition, as the dimensionality of the system is effectively changed from *d* = 2 (a square lattice) to *d* = 1 (a collection of uncoupled chains). However, the Floquet engineering framework uses a Hamiltonian, which is not directly available in non-reciprocal systems.

The Hamiltonian formalism from the ‘Constrained Hamiltonian embedding’ section proves convenient to analyse the driven system due to its symplectic structure. Adding the auxiliary spin **a**_*j*_, we construct the time-periodic Hamiltonian embedding

9 $$\begin{array}{rcl}H\left(t\right) & = & -\mathop{\sum }\limits_{\langle ij\rangle }\,{J}_{ij}({\theta }_{i})\left[\cos ({\theta }_{i}-{\theta }_{j}-{A}_{ij}\cos \omega t)\right.\\ & & +\left.\cos ({\varphi }_{i}-{\theta }_{j}-{A}_{ij}\cos \omega t)\right]+(i\leftrightarrow j),\end{array}$$

with $\left\{{\theta }_{j},{\varphi }_{i}\right\}={\delta }_{ij}$. To derive an effective description at high driving frequencies, *J*_*ij*_/*ω* ≪ 1, we apply the Floquet–Magnus expansion ${H}_{F}={H}_{F}^{(0)}+{\mathcal{O}}({\omega }^{-1})$ (ref. ^45^); the leading-order Floquet Hamiltonian is the time-average

10 $$\begin{array}{rcl}{H}_{F}^{(0)} & = & -\mathop{\sum }\limits_{\langle ij\rangle }{{\mathcal{J}}}_{0}\left({A}_{ij}\right){J}_{ij}({\theta }_{i})\left[\cos \left({\theta }_{i}-{\theta }_{j}\right)+\cos \left({\varphi }_{i}-{\theta }_{j}\right)\right]\\ & & +(i\leftrightarrow j),\end{array}$$

where ${{\mathcal{J}}}_{0}\left({A}_{ij}\right)$ is the Bessel function of the first kind. Higher-order terms in the Floquet–Magnus expansion can be readily obtained in a straightforward way using this formalism; they control the validity of the approximation.

To verify the validity of the Floquet Hamiltonian description in the non-reciprocal setting, we study the average magnetization as a function of time *t* for two drive frequencies in Fig. 5c,d. The deviation between the results from the exact Langevin equations of motion (purple) and the effective equations of motion derived from ${H}_{F}^{(0)}$ (orange) decreases as the frequency increases (from Fig. 5c to Fig. 5d); moreover, the drive modifies the behaviour significantly (compare orange and green curves).

By tuning the amplitude *A*_0_ of the drive close to a zero *z*_α_ of the Bessel function, ${{\mathcal{J}}}_{0}({z}_\alpha)=0$, one can suppress spin interactions in the *x*-direction, thereby realizing a dimensional crossover from a two-dimensional lattice to a set of one-dimensional chains (Fig. 5a). Figure 5b shows the average magnetization obtained from simulations of the spin dynamics (equation (8)) in the high-frequency regime as a function of temperature *T*. As we gradually increase the drive amplitude *A*_0_, the transition temperature *T*_*c*_ decreases. To leading order in *ω*^−1^, when $A_0=z_{\alpha} a^{-1}$, the system decouples into a set of independent one-dimensional *XY* chains and no longer exhibits an ordered phase at any finite reservoir temperature. The behaviour in Fig. 5b is consistent with our analysis using the Floquet Hamiltonian ${H}_{F}^{(0)}$, because the dimensional crossover occurs precisely at the zeroes of the Bessel function. Finally, note that, even though periodic drives typically lead to enhanced energy absorption, the heating rates are either power-law or exponentially suppressed with increasing the drive frequency *ω* (ref. ^63^).

In summary, Floquet Hamiltonian engineering based on the framework from the ‘Constrained Hamiltonian embedding’ section provides enhanced control over model parameters in non-reciprocal systems, enabling access to wider regions of their phase diagrams and allowing one to probe dimensional crossovers. More broadly, the Hamiltonian embedding—of which computing the Floquet Hamiltonian is one application—unlocks generic Hamiltonian methods for non-reciprocal systems. We anticipate its symplectic structure to similarly enable the transfer of other techniques to non-reciprocal systems.

## Discussion and outlook

In the past, isolated examples of Hamiltonian descriptions of non-reciprocal interactions have been reported, such as for the Lotka–Volterra predator–prey model^64^. In this work, we have constructed a general Hamiltonian embedding for non-reciprocal systems by introducing auxiliary degrees of freedom that couple to the original ones in a reciprocal way. By imposing a constraint on the phase-space dynamics of the embedding, we preserve the total number of independent degrees of freedom and recover the original non-reciprocal interactions. As a direct consequence of our construction, it follows that the phase space of the reciprocal Hamiltonian *H* from equation (2) contains a non-reciprocal submanifold defined by the constraint, which is closed under the dynamics.

Crucially, our construction works for pairwise interactions, and may not apply to arbitrary non-potential systems (Fig. 1); a generalization to velocity-dependent (for example, Lorentz-like) forces is presented in the Supplementary Information. Unlike in systems where non-reciprocity arises as a result of projecting/tracing out bath degrees of freedom that mediate the interactions (for example, a chemical concentration field)^1^, we emphasize that here the auxiliary degree of freedom does not play the role of a bath for the original one; instead, the projecting/tracing out procedure is replaced by the constraint. The framework is generic and applies to both inertial and dissipative dynamics featuring non-reciprocal pairwise interactions.

Our Hamiltonian embedding advances a century-old effort of using auxiliary degrees of freedom to describe generic non-conservative forces. First attempts date back to Bateman^65^, where a Lagrangian was constructed for a dissipative oscillator; however, these early results cannot be generalized beyond harmonic oscillators as they neglect the dynamical constraint from equation (5). Markovian embeddings have also been frequently used to describe and simulate non-Markovian systems with memory kernels^66–68^. More recently, Galley presented a general formalism to describe non-conservative forces by formulating Hamilton’s principle as an initial value problem^55^: it requires doubling the degrees of freedom and relies on an educated guess for the interaction term that couples them. This approach produces the correct non-reciprocal equations of motion, when supplemented with our Hamiltonian (equation (2)). A key contribution of our work is the generic construction of the interaction energy between the original and the auxiliary degrees of freedom for any pairwise non-reciprocal interaction; previous literature has not discussed non-reciprocal interactions within a Hamiltonian framework. Critically, our approach differs from Galley’s in the implementation: Galley imposes an equality condition on the degrees of freedom at the final time, from which the so-called physical limit^55^ arises by setting the boundary terms in the variational principle to vanish; the conceptual difference in our approach is the constraint that we impose at the initial time, which is then automatically preserved by the dynamics at all times.

We discussed two applications using *XY* spins on a square lattice with vision-cone interactions: (1) We proved the equivalence between Glauber dynamics based on the Hamiltonian embedding and the original non-reciprocal Langevin dynamics. In particular, we showed that both procedures yield the same critical temperature for the transition between the ferromagnetic and non-magnetized phases. These results open the door to investigating the nature of this and similar phase transitions in large systems using fast Monte-Carlo algorithms. (2) We make use of the inherent symplectic structure of the embedding to derive an effective Hamiltonian for periodically driven vision-cone *XY* spins using Floquet theory; this allows us to interpret the disappearance of ferromagnetic order when varying the drive amplitude as a dimensional crossover. These results suggest exciting possibilities for experiments to apply Floquet engineering techniques^45,57,58^ to investigate regimes in parameter space that are otherwise inaccessible in the non-reciprocal system.

We note that the Hamiltonian embedding does not introduce new physics content per se beyond what is already captured by the equations of motion defining the original non-reciprocal system. However, it enables the study of non-reciprocal systems using analytical and numerical tools from equilibrium or non-equilibrium physics that require a Hamiltonian structure. For instance, one can now use Monte-Carlo algorithms that shortcut long transients of Langevin simulations and lead directly to the steady state. Numerical techniques based on the embedding may require keeping track of twice the number of original degrees of freedom; however, the numerical complexity of any algorithm remains unchanged by adding the auxiliary degrees of freedom (which effectively doubles the system size). Likewise, one can apply various canonical transformations (for example, Schrieffer–Wolff-like transformations such as the Birkhoff–Gustavson normal form^38–40^) to identify fast and slow effective degrees of freedom in non-reciprocal models. An illustrative example is the Floquet–Magnus expansion for periodically driven systems: higher-order terms in the inverse drive frequency contain effective spin–spin interactions^45^. Note that, whereas it is possible to derive effective stroboscopic equations of motion in the high-frequency regime directly from the equations of motion to any order in the inverse frequency *ω*^−1^ by using two-times perturbation theory^69^, the Hamiltonian formulation has the advantage of providing a systematic model-agnostic algorithmic procedure. This formalism can prove useful to analyse the behaviour of time-crystalline non-reciprocal states^70^.

The Hamiltonian framework we propose has the potential to expand the scope of research on non-reciprocal systems and enable novel applications. In experiments that simulate classical and quantum models^27,71–73^, our construction can offer a new way to engineer non-reciprocal dynamics using reciprocal Hamiltonian systems subject to a constraint. Promising future directions include developing kinetic Monte-Carlo schemes to capture non-steady states^41^, canonically quantizing the Hamiltonian embedding, or using it to construct statistical mechanics and thermodynamic descriptions^74,75^ of non-reciprocal systems.

## Methods

### Equivalence between constrained Glauber Monte-Carlo and Langevin dynamics

The constrained Glauber Monte-Carlo algorithm with transition rates defined using the Hamiltonian embedding and the Langevin equation with generic non-reciprocal interactions, share the same Fokker–Planck equation. This equation describes the configuration space density distribution $\rho (\boldsymbol{\theta },t)$, with $\boldsymbol{\theta }=({\theta }_{i},\ldots ,{\theta }_{{L}^{2}})$. We now demonstrate this equivalence explicitly.

First, we sketch the derivation of the Fokker–Planck equation from the Langevin equation. Denoting the force acting on spin *θ*_*i*_ by ${F}_{i}(\boldsymbol{\theta })$, and using Itô formalism, the equation of motion is

11 $${\theta }_{i}(t+\Delta t)-{\theta }_{i}(t)={F}_{i}(\boldsymbol{\theta })\,\Delta t+\sqrt{2T}\,\Delta {W}_{i}(t)\,,$$

where Δ*W*_*i*_(*t*) is a Wiener increment obeying 〈Δ*W*_*i*_〉 = 0 and 〈Δ*W*_*i*_ Δ*W*_*j*_〉 = *δ*_*ij*_Δ*t*. Following the standard steps, we arrive at the Fokker–Planck equation for the configuration-space probability density $\rho (\boldsymbol{\theta },t)$:

12 $${\partial }_{t}\rho (\boldsymbol{\theta },t)=-\mathop{\sum }\limits_{i}{\partial }_{{\theta }_{i}}\left[{F}_{i}(\boldsymbol{\theta })\,\rho (\boldsymbol{\theta },t)\right]+T\mathop{\sum }\limits_{i}{\partial }_{{\theta }_{i}}^{2}\rho (\boldsymbol{\theta },t).$$

In what follows, we independently derive equation (12) from the Glauber dynamics with transition probability rates

13 $$w({\theta }_{i}\to {\theta }_{i}^{{\prime} })=\frac{1}{2}\left(1-\tanh \frac{\Delta E(\theta ,{\theta }^{{\prime} })}{2T}\right),$$

14 $$\Delta E(\theta ,{\theta }^{{\prime} })=\frac{1}{2}{\left.\left(H({\theta }^{{\prime} },\varphi )-H(\theta ,{\varphi }^{{\prime} })\right)\right|}_{{\rm{constr}}.(\theta ,\varphi )}$$

defined by eliminating the auxiliary degree of freedom from the Hamiltonian embedding by enforcing the constraint. We note that *T* here is the temperature of the bath; the non-reciprocal system of interest is never in a thermal state at any point during its evolution.

In each Glauber update step *n*, we pick a spin at random and update it according to ${\theta }_{j}\to {\theta }_{j}^{{\prime} }={\theta }_{j}+\delta$. The probability of selecting spin *j* is uniformly distributed on the square lattice of linear size *L*, and is hence given by 1/*L*^2^. At the same time, the update angle *δ* is drawn uniformly from the interval [−Δ, Δ], with probability density 1/(2Δ).

The discrete-time master equation governing these Glauber dynamics is then given by

15 $$\begin{array}{ll}\rho (\boldsymbol{\theta },n+1)-\rho (\boldsymbol{\theta },n)=\displaystyle\frac{1}{2\Delta }{\displaystyle\int }_{-\Delta }^{\Delta }{\rm{d}}\delta \displaystyle\frac{1}{{L}^{2}}\mathop{\sum }\limits_{i}\left[\rho ({\theta }_{i}+\delta ,{\{\boldsymbol{\theta }\}}_{j\ne i},n)\right.\\\qquad\qquad\qquad\qquad\qquad\left.w({\theta }_{i}+\delta \to {\theta }_{i})-\rho (\boldsymbol{\theta },n)\,w({\theta }_{i}\to {\theta }_{i}+\delta )\right].\end{array}$$

Treating Δ ≪ 1 as a small parameter, we can expand the integrand to leading order in *δ* using

16 $$\begin{array}{rcl}\rho ({\theta }_{i}+\delta ,{\{\boldsymbol{\theta }\}}_{j\ne i},n) & = & \rho (\boldsymbol{\theta },n)+{\partial }_{{\theta }_{i}}\rho (\boldsymbol{\theta },n)\delta +\frac{1}{2}{\partial }_{{\theta }_{i}}^{2}\rho (\boldsymbol{\theta },n){\delta }^{2}\\ & & +{\mathcal{O}}({\delta }^{3})\,,\\ w({\theta }_{i}\to {\theta }_{i}+\delta ) & = & \frac{1}{2}+\frac{1}{4T}{F}_{i}(\boldsymbol{\theta })\delta +\frac{1}{8T}{\partial }_{{\theta }_{i}}{F}_{i}(\boldsymbol{\theta }){\delta }^{2}\\ & & +{\mathcal{O}}({\delta }^{3})\,,\\ w({\theta }_{i}+\delta \to {\theta }_{i}) & = & \frac{1}{2}-\frac{1}{4T}{F}_{i}(\boldsymbol{\theta })\delta -\frac{1}{8T}{\partial }_{{\theta }_{i}}{F}_{i}(\boldsymbol{\theta }){\delta }^{2}\\ & & +{\mathcal{O}}({\delta }^{3})\,,\end{array}$$

where the force acting on spin *i* is obtained from the embedding Hamiltonian under the constraint as

17 $$\begin{array}{rcl}{F}_{i}(\boldsymbol{\theta }) & = & -\frac{1}{2}\left({\partial }_{{\theta }_{i}}-{\partial }_{{\varphi }_{i}}\right)H{\left.(\boldsymbol{\theta },{\bf{\varphi }})\right|}_{\mathrm{constr}.(\theta ,\varphi )}\\ & = & -{\partial }_{{\theta }_{i}}H{\left.(\boldsymbol{\theta },{\bf{\varphi }})\right|}_{\mathrm{constr}.(\theta ,\varphi )}\,.\end{array}$$

We emphasize that one first computes the derivatives and only then applies the constraint.

Noticing that linear terms in *δ* vanish because the integral limits are symmetric, we obtain to leading order in Δ:

18 $$\begin{array}{rcl} & & \rho (\boldsymbol{\theta },n+1)-\rho (\boldsymbol{\theta },n)\\ & & \approx \frac{1}{2\Delta {L}^{2}}{\displaystyle\int }_{-\Delta }^{\Delta }{\delta }^{2}{\rm{d}}\delta \displaystyle\mathop{\sum }\limits_{i}\frac{1}{4T}{\partial }_{{\theta }_{i}}\left[-\rho (\boldsymbol{\theta },n){F}_{i}(\boldsymbol{\theta })+T{\partial }_{{\theta }_{i}}\rho (\boldsymbol{\theta },n)\right]\\ & & =\frac{1}{{L}^{2}}\frac{{\Delta }^{2}}{12T}\displaystyle\mathop{\sum }\limits_{i}{\partial }_{{\theta }_{i}}\left[-\rho (\boldsymbol{\theta },n){F}_{i}(\boldsymbol{\theta })+T{\partial }_{{\theta }_{i}}\rho (\boldsymbol{\theta },n)\right]+{\mathcal{O}}({\Delta }^{4}).\end{array}$$

Recalling that in equation (11) all *L*^2^ spins get updated simultaneously at each time step, we define dimensionless Monte-Carlo time as

19 $${t}_{{\rm{MC}}}=\frac{n}{{L}^{2}}.$$

In the limit of large system sizes, *L*^2^ ≫ 1, the corresponding time increment d*t*_MC_ = 1/*L*^2^ is small, and Monte-Carlo time becomes continuous; we obtain

20 $$\rho (\boldsymbol{\theta },n+1)-\rho (\boldsymbol{\theta },n)={\partial }_{{t}_{\mathrm{MC}}}\rho (\boldsymbol{\theta },{t}_{\mathrm{MC}})\,{\rm{d}}{t}_{\mathrm{MC}}.$$

This allows us to formally define a continuous-time limit for the master equation:

21 $$\begin{array}{rcl}\frac{\partial \rho (\boldsymbol{\theta },{t}_{\mathrm{MC}})}{\partial {t}_{\mathrm{MC}}} & = & \frac{{\Delta }^{2}}{12T}\displaystyle\mathop{\sum }\limits_{i}{\partial }_{{\theta }_{i}}\left[-\rho (\boldsymbol{\theta },{t}_{\mathrm{MC}}){F}_{i}(\boldsymbol{\theta })+T{\partial }_{{\theta }_{i}}\rho (\boldsymbol{\theta },{t}_{\mathrm{MC}})\right]\\ & & +{\mathcal{O}}({\Delta }^{4}).\end{array}$$

In the small-update regime Δ ≪ 1, equation (21) is equivalent to the Fokker–Planck equation derived earlier for the Langevin dynamics (equation (12)), except for the prefactor Δ^2^/(12*T*).

In units where the friction coefficient is set to unity, it follows from the equations of motion that energy has the same unit as frequency (or inverse time) (see, for example, equation (1)). Because the only model-independent energy scale in the Glauber rate *w* is the temperature *T* (which also has units of energy), the physical time unit in equation (21) is set by the inverse bath temperature *T*. Hence, Monte-Carlo time *t*_MC_ is related to the physical (Langevin) time *t*_Lan_ by

22 $${t}_{{\rm{Lan}}}=\frac{{\Delta }^{2}}{12T}{t}_{{\rm{MC}}}.$$

Therefore, starting with Glauber dynamics using the transition rates obtained from the Hamiltonian embedding, we derived the Fokker–Planck equation (12) which describes the Langevin dynamics of the original non-reciprocal system. This establishes the equivalence between the two approaches. We present a detailed formal derivation that uses the extended embedding variables in the Supplementary Information.

Finally, we note that the derivation above does not make use of the details of a particular model. For as long as one can construct a Hamiltonian embedding, the latter can be used to define transition rates *w* using equation (13), and the equivalence of the Langevin and Monte-Carlo descriptions follows.

### Hamiltonian embedding for chase-and-run spin model

Consider *XY* spins on a square lattice of linear size *L* with periodic boundary conditions. Denoting the lattice sites by *i* = (*x*, *y*), the corresponding Langevin equation of motion for the chase-and-run spin model discussed in the main text reads as

23 $$\begin{array}{l}{\dot{\theta}}_{x,y} \,= -{J}_{\to}\sin ({\theta }_{x,y}-{\theta}_{x+1,y})-{J}_{\leftarrow}\sin ({\theta}_{x,y}-{\theta}_{x-1,y})\\ \qquad \quad \,-{J}_{\mathrm{rec}}[\sin ({\theta}_{x,y}-{\theta}_{x,y+1})+\sin ({\theta}_{x,y}-{\theta}_{x,y-1})]\\ \qquad \quad \, +\sqrt{2T}{\eta}_{x,y}(t)\,.\end{array}$$

Here *J*_←_ ≠ *J*_→_ are the non-reciprocal interactions along the *x*-direction, and *J*_rec_ denotes the reciprocal interactions along the *y*-direction (see main text). Temperature is denoted by *T*, and *η*_*x*,*y*_(*t*) adds white noise to the dynamics of each spin (equation (1)).

The spins are initialized in the perfect stripe state defined by

24 $${\theta }_{x,y}=\left\{\begin{array}{ll}0, & f\mathrm{or}\,\,x\in \left(\frac{L}{4},\frac{3L}{4}\right)\\ \frac{{\rm{\pi }}}{2}, & \mathrm{else}\end{array}\right.,$$

which is perturbed by the noise in the dynamics.

The Hamiltonian embedding for the non-reciprocal *XY* spins in equation (23) reads as

25 $$\begin{array}{rcl}{H}_{{\rm{tot}}} & = & {H}_{SS}+{H}_{Sa}-{H}_{aa},\\ {H}_{SS} & = & -\mathop{\sum }\limits_{x,y}({J}_{\to }+{J}_{\leftarrow })[\cos ({\theta }_{x,y}-{\theta }_{x+1,y})+\cos ({\theta }_{x,y}-{\theta }_{x-1,y})]\\ & & +{J}_{{\rm{rec}}}[\cos ({\theta }_{x,y}-{\theta }_{x,y+1})+\cos ({\theta }_{x,y}-{\theta }_{x,y-1})],\\ {H}_{Sa} & = & -\mathop{\sum }\limits_{x,y}{J}_{\leftarrow }\cos ({\theta }_{x,y}-{\varphi }_{x+1,y})+{J}_{\to }\cos ({\theta }_{x,y}-{\varphi }_{x-1,y}),\\ {H}_{aa} & = & -\mathop{\sum }\limits_{x,y}{J}_{{\rm{rec}}}[\cos ({\varphi }_{x,y}-{\varphi }_{x,y+1})+\cos ({\varphi }_{x,y}-{\varphi }_{x,y-1})],\end{array}$$

where $\{{\theta }_{x,y},{\varphi }_{{x}^{{\prime} },{y}^{{\prime} }}\}={\delta }_{x,{x}^{{\prime} }}{\delta }_{y,{y}^{{\prime} }}$ are conjugate variables.

A straightforward calculation shows that the Hamilton equations of motion derived from equation (25) reduce to the Langevin equation (23), when subject to the constraint

26 $${\theta }_{x,y}-{\varphi }_{x,y}={\rm{\pi }}\,.$$

Thus, the energy difference used in the Glauber update rule can be evaluated following equation (6) as

27 $$\begin{array}{rcl}\Delta E({\theta }_{x,y},{\theta }_{x,y}^{{\prime} }) & = & -{J}_{\to }\left[\cos \left({\theta }_{x,y}^{{\prime} }-{\theta }_{x+1,y}\right)-\cos ({\theta }_{x,y}-{\theta }_{x+1,y})\right]\\ & & -{J}_{\leftarrow }\left[\cos \left({\theta }_{x,y}^{{\prime} }-{\theta }_{x-1,y}\right)-\cos ({\theta }_{x,y}-{\theta }_{x-1,y})\right]\\ & & -{J}_{{\rm{rec}}}\mathop{\sum }\limits_{\sigma =\pm 1}\left[\cos \left({\theta }_{x,y}^{{\prime} }-{\theta }_{x,y+\sigma }\right)\right.\\ & & -\left.\cos ({\theta }_{x,y}-{\theta }_{x,y+\sigma })\right]\,.\end{array}$$

We use this expression in the constrained Glauber simulations of the chase-and-run dynamics.

## Online content

Any methods, additional references, Nature Portfolio reporting summaries, source data, extended data, supplementary information, acknowledgements, peer review information; details of author contributions and competing interests; and statements of data and code availability are available at 10.1038/s41567-026-03317-0.

## Supplementary information

Supplementary Information Supplementary Discussion and Supplementary Figs. 1–9.

Peer Review File

Supplementary Video 1 Non-stationary state of *XY* spins with chase-and-run interactions. *XY* spins are placed on a square lattice with non-reciprocal interactions along the *x*-direction, and reciprocal interactions along the *y*-direction. The video shows the time evolution of a stripe pattern used as an initial steady state (see colour bar for spin orientation), which moves perpetually to the left due to the non-reciprocal interactions. Left: Langevin dynamics simulated using Heun’s method. Right: constrained Glauber dynamics with transition rates obtained from the embedding Hamiltonian. Time (see panel captions) is rescaled by the linear system size *L*. The video shows an excellent agreement between the two approaches. The parameters are *J*_→_/*J* = 1, *J*_←_/*J* = − 0.99, *J*_rec_/*J* = 10, *T*/*J* = 0.08, Δ = 0.05 and *L* = 200, with the scaling factor *α*(Δ, *T*) = 2.37 × 10^−3^ extracted numerically.

## Data Availability

The data associated with this Article are available via Zenodo at 10.5281/zenodo.19427734 (ref. ^76^).

## Code availability

The codes associated with this Article are available via Zenodo at 10.5281/zenodo.19427734 (ref. ^76^).
