# Supplementary material for: Hamiltonian description of non-reciprocal interactions
Source: Nat Phys. 2026 Jun 12;22(8):1350–9. doi: 10.1038/s41567-026-03317-0 (PMC13461368; doi:10.1038/s41567-026-03317-0)
Supplement: Supplementary file 1 [file 41567_2026_3317_MOESM1_ESM.pdf]

nature physics

Supplementary information https://doi.org/10.1038/s41567-026-03317-0

Hamiltonian description of non-reciprocal interactions

In the format provided by the authors and unedited

CONTENTS

S1. Constrained Hamiltonian embedding of nonreciprocal systems 2
A. Velocity-independent interaction forces 2
B. Velocity-dependent interaction forces 3
1. General form of velocity-dependent Lagrangians with reciprocal interactions 3
2. A direct Lagrangian embedding for velocity-dependent nonreciprocal forces 5
3. Velocity-dependent nonreciprocal interactions: General framework 7
4. Example: Lorentz-like forces 8

S2. Examples of nonreciprocal systems treated within the Hamiltonian embedding 9
A. XY spins with vision cone interactions 9
B. Metal-dielectric Janus particles 10
C. Self-aligning active particles 12
D. Programmed robotic metamaterials 13
E. Feedback-controlled active particles with vision-cone interactions 13
F. Sedimenting particles with hydrodynamic interactions 14

S3. Symplectic structure of the Hamiltonian embedding: Implementing the constraint for interactions with overdamped and undamped nonreciprocal dynamics 15
A. Overdamped nonreciprocal systems 15
B. Undamped nonreciprocal systems 16

S4. Equivalence between nonreciprocal Langevin dynamics and Glauber Monte-Carlo dynamics 18
A. Langevin steady-state distribution 19
1. Steady-state distribution 19
2. Time-dependent non-stationary distribution 21
3. Eliminating the auxiliary degree of freedom from Langevin dynamics 22
B. Glauber dynamics description 22
1. Steady-state distribution from global balance 23
2. Non-stationary distributions from Monte-Carlo Glauber dynamics 24
3. Eliminating the auxiliary degree of freedom from the Glauber transition rates 26

S5. Determining the critical temperature for the vision-cone model 27

S6. Supplementary results for the Monte-Carlo simulations 27
A. Constrained Glauber algorithm 27
B. Constrained Glauber algorithm after eliminating the auxiliary spins 28
C. Finite-size effects 29
D. Glauber dynamics without auxiliary spins 30
E. Soft-vision-cone interactions: Comparison between selfish energy and constrained Glauber dynamics 30

S7. Chase-and-run dynamics for nonreciprocal XY spins 31
A. Langevin dynamics 33
B. Constrained Glauber dynamics 34
1. Monte-Carlo time vs. physical time 35
2. Order-parameter dynamics 35

S8. Entropy production in the Hamiltonian embedding 36

References 37

S1. CONSTRAINED HAMILTONIAN EMBEDDING OF NONRECIPROCAL SYSTEMS

In this section, we first outline a general approach for constructing Hamiltonian embeddings, focusing on velocity-independent interaction forces. We then discuss the more general case of velocity-dependent interactions.

A. Velocity-independent interaction forces

We begin by emphasizing that, in its simplest form, (non-)reciprocity is a property defined for *pairwise interacting* systems. Let us denote by $\mathbf{F}_{i\to j}(\mathbf{r}_i, \mathbf{r}_j)$ the force exerted by particle i at position $\mathbf{r}_i$ on particle j at position $\mathbf{r}_j$. We consider translation invariant systems where $\mathbf{F}_{i\to j}(\mathbf{r}_i, \mathbf{r}_j) = \mathbf{F}_{i\to j}(\mathbf{r}_i - \mathbf{r}_j)$ depends only on the distance between the two particles involved. In addition, in this first section, we only consider momentum-independent forces, and thus leave out of consideration Lorentz-like forces.

Reciprocal (R) interactions obey Newton's third law

$$\mathbf{F}^{\mathrm{R}}_{i\to j}(\mathbf{r}_i - \mathbf{r}_j) = -\mathbf{F}^{\mathrm{R}}_{j\to i}(\mathbf{r}_j - \mathbf{r}_i), \tag{S.1}$$

i.e, the force exerted by particle i on particle j, $\mathbf{F}^{\mathrm{R}}_{i\to j}(\mathbf{r}_i - \mathbf{r}_j)$, is equal in magnitude and opposite in direction to the force $\mathbf{F}^{\mathrm{R}}{}_{j\to i}(\mathbf{r}_j - \mathbf{r}_i)$ exerted by particle j on particle i.

We define nonreciprocal interactions via the relation

$$\mathbf{F}^{\mathrm{NR}}_{i\to j}(\mathbf{r}_i - \mathbf{r}_j) \neq -\mathbf{F}^{\mathrm{NR}}_{j\to i}(\mathbf{r}_j - \mathbf{r}_i). \tag{S.2}$$

Nonreciprocal interactions refer to an asymmetry in the interaction between two particles in which the action and reaction are not equal.

In general, the equations of motion for particle i may be governed by a combination of reciprocal and nonreciprocal interactions. Hence in its most general form, it takes the form:

$$m\ddot{\mathbf{r}}_i + \gamma\dot{\mathbf{r}}_i = \sum_{j\neq i}\left[\mathbf{F}^{\mathrm{NR}}_{j\to i}(\mathbf{r}_i - \mathbf{r}_j) + \mathbf{F}^{\mathrm{R}}_{j\to i}(\mathbf{r}_i - \mathbf{r}_j)\right], \tag{S.3}$$

where we denote the mass by m and the damping factor by γ.

We now assume that the interaction forces can be derived from a one-way interaction potential:

$$\mathbf{F}_{i\to j}(\mathbf{r}_i - \mathbf{r}_j) = -\frac{\partial}{\partial \mathbf{r}_i} U_{i\to j}(\mathbf{r}_i - \mathbf{r}_j). \tag{S.4}$$

Reciprocal interactions are conservative and share the same (mutual) interaction potential, and hence obey the relation $U^{\mathrm{R}}_{i\to j} = U^{\mathrm{R}}_{j\to i}$ which, therefore, implies Newton's third law (Eq. (S.1)); it is common to use the simplified notation $U^{\mathrm{R}}_{i\to j} = U^{\mathrm{R}}$. By contrast, nonreciprocal interactions obey $U^{\mathrm{NR}}_{i\to j} \neq U^{\mathrm{NR}}_{j\to i}$, and are hence nonconservative. Since each interaction function $U^{\mathrm{NR}}_{i\to j}(\mathbf{r}_i - \mathbf{r}_j)$, $U^{\mathrm{NR}}_{j\to i}(\mathbf{r}_i - \mathbf{r}_j)$ depends on the positions of both particles involved, one cannot write down an energy function such that the Hamiltonian equations of motion for the corresponding conjugate variables are nonreciprocal; this is because the resulting force acting on particle i will inevitably contain the gradients of both interaction potentials, which would make the net force reciprocal.

To resolve this problem, we introduce a copy of the system described by the auxiliary degrees of freedom $\{\mathbf{x}_i, \mathbf{p}^{\mathbf{x}}_j\} = \delta_{ij}$. The original degrees of freedom obey the Poisson bracket $\{\mathbf{r}_i, \mathbf{p}^{\mathbf{r}}_j\} = \delta_{ij}$. One can construct a general Hamiltonian embedding for the joint system using only reciprocal interactions, as follows:

$$\begin{aligned} H = \quad & \sum_j \frac{\left(\mathbf{p}^{\mathbf{r}}_j - \frac{\gamma}{2}\mathbf{x}_j\right)^2}{2m} - \frac{\left(\mathbf{p}^{\mathbf{x}}_j + \frac{\gamma}{2}\mathbf{r}_j\right)^2}{2m} \\ & + \sum_{\langle ij\rangle}\left[U^{\mathrm{NR}}_{i\to j}(\mathbf{r}_i - \mathbf{r}_j) + U^{\mathrm{NR}}_{j\to i}(\mathbf{r}_j - \mathbf{r}_i) - U^{\mathrm{NR}}_{i\to j}(\mathbf{x}_j - \mathbf{r}_i) - U^{\mathrm{NR}}_{j\to i}(\mathbf{x}_i - \mathbf{r}_j)\right] \\ & + \sum_{\langle ij\rangle}\left[U^{\mathrm{R}}(\mathbf{r}_i - \mathbf{r}_j) - U^{\mathrm{R}}(\mathbf{x}_i - \mathbf{x}_j)\right]. \end{aligned} \tag{S.5}$$

The notation $\langle ij\rangle$ denotes a summation over pairs of particles i and j (each counted once). Here the kinetic energy is quadratic in the momenta $\mathbf{p}^{\mathbf{r}}_j, \mathbf{p}^{\mathbf{x}}_j$, and hence the system is inertial and the EOM is second order in time. Note the apparent similarity of the

kinetic energy above to that of a particle in a magnetic field; however, a notable difference is that, in the Hamiltonian H above, the minimal coupling is between $(\mathbf{p}^{\mathbf{r}}_j, \mathbf{x}_j)$ [as opposed to $(\mathbf{p}^{\mathbf{r}}_j, \mathbf{r}_j)$], etc.

As we showed in the Main Text, in general, the equations of motion derived from the Hamiltonian H defines a coupled system of equations

$$
\begin{aligned}
\frac{\mathrm{d}\mathbf{r}_i}{\mathrm{d}t} &= \frac{\mathbf{p}^{\mathbf{r}}_i - \frac{\gamma}{2}\mathbf{x}_i}{m} , \\
\frac{\mathrm{d}\mathbf{p}^{\mathbf{r}}_i}{\mathrm{d}t} &= \frac{\gamma}{2}\frac{\left(\mathbf{p}^{\mathbf{x}}_i + \frac{\gamma}{2}\mathbf{r}_i\right)}{m} - \frac{\partial}{\partial \mathbf{r}_i}\sum_{j\in\langle ij\rangle}\left[U^{\mathrm{NR}}_{i\to j}(\mathbf{r}_i - \mathbf{r}_j) + U^{\mathrm{NR}}_{j\to i}(\mathbf{r}_j - \mathbf{r}_i) - U^{\mathrm{NR}}_{i\to j}(\mathbf{x}_j - \mathbf{r}_i) + U^{\mathrm{R}}(\mathbf{r}_i - \mathbf{r}_j)\right] , \\
\frac{\mathrm{d}\mathbf{x}_i}{\mathrm{d}t} &= -\frac{\mathbf{p}^{\mathbf{x}}_i + \frac{\gamma}{2}\mathbf{r}_i}{m} , \\
\frac{\mathrm{d}\mathbf{p}^{\mathbf{x}}_i}{\mathrm{d}t} &= \frac{\gamma}{2}\frac{\left(\mathbf{p}^{\mathbf{r}}_i - \frac{\gamma}{2}\mathbf{x}_i\right)}{m} + \frac{\partial}{\partial \mathbf{x}_i}\sum_{j\in\langle ij\rangle}\left[U^{\mathrm{NR}}_{j\to i}(\mathbf{x}_i - \mathbf{r}_j) + U^{\mathrm{R}}(\mathbf{x}_i - \mathbf{x}_j)\right] .
\end{aligned}
\tag{S.6}
$$

However, if we impose the constraint

$$\mathbf{r}_i(0) - \mathbf{x}_i(0) = 0, \qquad \mathbf{p}^{\mathbf{r}}_i(0) + \mathbf{p}^{\mathbf{x}}_i(0) = 0, \tag{S.7}$$

at time $t = 0$, it follows immediately from the above equations of motion that the constraint is preserved at all times, i.e.,

$$\frac{\mathrm{d}}{\mathrm{d}t}\left[\mathbf{r}_i(t) - \mathbf{x}_i(t)\right]\Big|_{\text{constr.}} = 0, \qquad \frac{\mathrm{d}}{\mathrm{d}t}\left[\mathbf{p}^{\mathbf{r}}_i(t) + \mathbf{p}^{\mathbf{x}}_i(t)\right]\Big|_{\text{constr.}} = 0. \tag{S.8}$$

Moreover, the joint equations of motion reduce to the original nonreciprocal dynamics in Eq. (S.3). More precisely, each degree of freedom (i.e., both the $\mathbf{r}_j$ and the $\mathbf{x}_j$ degree of freedom) follows the same equations of motion; the dynamics of the $\mathbf{x}_j$ degree of freedom perfectly mirrors that of $\mathbf{r}_j$. We note that the negative kinetic energy for the auxiliary $\mathbf{x}_j$ is a sufficient condition to preserve the constraint at all times.

In Sec. S2 below, we give the explicit form of the constrained Hamiltonian for various models with nonreciprocal equations of motion.

B. Velocity-dependent interaction forces

In Sec. S1 A, we restricted our attention to velocity-independent interactions. We now consider pairwise interactions that may depend on particle velocities, while still requiring the equations of motion to remain second order in time. This requirement severely constrains the admissible form of the interaction Lagrangian and will be the starting point for the embedding constructions discussed below.

1. General form of velocity-dependent Lagrangians with reciprocal interactions

We begin by reviewing a few important facts about velocity-dependent reciprocal interactions. We will then build on these ideas to define the embedding for their nonreciprocal counterparts.

Consider an inertial system of N particles with coordinates $\{\mathbf{r}_i\}$ and Lagrangian

$$\mathcal{L}(\{\mathbf{r}\}, \{\dot{\mathbf{r}}\}) = \sum_{i=1}^{N}\frac{m_i}{2}\dot{\mathbf{r}}_i^2 - \mathcal{U}(\{\mathbf{r}\}, \{\dot{\mathbf{r}}\}), \tag{S.9}$$

where $\mathcal{U}$ denotes the interaction energy. The Euler–Lagrange equations read as

$$m_i\ddot{\mathbf{r}}_i = -\partial_{\mathbf{r}_i}\mathcal{U} + \frac{\mathrm{d}}{\mathrm{d}t}\partial_{\dot{\mathbf{r}}_i}\mathcal{U} \equiv \mathbf{F}_i , \tag{S.10}$$

which defines the total force $\mathbf{F}_i$ acting on particle i. In general, the total-time-derivative term contains accelerations through $\left(\partial_{\dot{\mathbf{r}}_i}\partial_{\dot{\mathbf{r}}_j}\mathcal{U}\right)\ddot{\mathbf{r}}_j$, which is seen by carrying out the total time derivative. If we require the force $\mathbf{F}_i$ to depend only on positions and velocities, and not explicitly on accelerations, these terms must vanish identically along arbitrary solutions of the equations of motion. Therefore,

$$\partial_{\dot{\mathbf{r}}_i}\partial_{\dot{\mathbf{r}}_j}\mathcal{U} = 0 \qquad \forall\, i, j. \tag{S.11}$$

Hence, the interaction part of the Lagrangian can be at most linear in the velocities, and in its full generality takes the form

$$\mathcal{U}(\{\mathbf{r}\},\{\dot{\mathbf{r}}\}) = \phi(\{\mathbf{r}\}) + \sum_j \mathbf{A}_j(\{\mathbf{r}\})\cdot\dot{\mathbf{r}}_j, \tag{S.12}$$

where we introduced the scalar potential ϕ, and $\mathbf{A}_j$ are generalized vector potentials. Here, the notation and physical interpretation are borrowed from electromagnetism.

Substituting this form into the Euler–Lagrange equations yields

$$\mathbf{F}_i = -\partial_{\mathbf{r}_i}\phi - \sum_j \partial_{\mathbf{r}_i}\left[\mathbf{A}_j\cdot\dot{\mathbf{r}}_j\right] + \sum_j (\dot{\mathbf{r}}_j\cdot\partial_{\mathbf{r}_j})\mathbf{A}_i. \tag{S.13}$$

It is convenient to write the force in the α-th direction in the linear form

$$(\mathbf{F}_i)_\alpha = V_{i\alpha}(\{\mathbf{r}\}) + \sum_{j,\beta} T_{i\alpha}^{j\beta}(\{\mathbf{r}\})\,\dot{r}_{j\beta}, \tag{S.14}$$

with the definitions

$$V_{i\alpha} = -\partial_{r_{i\alpha}}\phi, \qquad T_{i\alpha}^{j\beta} = -\partial_{r_{i\alpha}}(\mathbf{A}_j)_\beta + \partial_{r_{j\beta}}(\mathbf{A}_i)_\alpha. \tag{S.15}$$

Conversely, if the force has this form and satisfies a set of integrability conditions, one can reconstruct the potentials locally. First, since $V_{i\alpha} = -\partial_{r_{i\alpha}}\phi$, the field must be a gradient field in order for ϕ to be well defined, i.e., $\partial_{r_{j\beta}}V_{i\alpha} = \partial_{r_{i\alpha}}V_{j\beta}$. Second, since the tensor $T_{i\alpha}^{j\beta}$ already satisfies the closure (Bianchi-type) condition, $\partial_{r_{k\gamma}}T_{i\alpha}^{j\beta} + \partial_{r_{i\alpha}}T_{k\gamma}^{j\beta} + \partial_{r_{j\beta}}T_{i\alpha}^{k\gamma} = 0$, the vector potential $\mathbf{A}_i$ can be reconstructed from the force in Eq. (S.14) by integrating $T_{i\alpha}^{j\beta}$ along a path Γ in configuration space. In particular,

$$\phi(\{\mathbf{r}\}) = -\int_\Gamma \sum_i \mathbf{V}_i(\{\mathbf{r}\})\cdot \mathrm{d}\mathbf{r}_i, \tag{S.16}$$

and, up to a gauge choice,

$$(\mathbf{A}_i)_\alpha(\{\mathbf{r}\}) = -\int \mathrm{d}^{3N}r' \left[\partial_{r'_{j\beta}} G_{3N}\left(\{\mathbf{r}\}-\{\mathbf{r}'\}\right)\right] T_{i\alpha}^{j\beta}\left(\{\mathbf{r}'\}\right) \tag{S.17}$$

where G_{3N} is a $(3N)$-dimensional Green's function obtained from solving the equation

$$-\sum_{i,\alpha}\nabla^2_{r_{i\alpha}} G_{3N}\left(\{\mathbf{r}\}-\{\mathbf{r}'\}\right) = \delta^{(3N)}\left(\{\mathbf{r}\}-\{\mathbf{r}'\}\right). \tag{S.18}$$

In the translation-invariant case, the tensor $T_{i\alpha}^{j\beta}$ depends only on the relative coordinate $\mathbf{r} = \mathbf{r}_i - \mathbf{r}_j$. Translational invariance then requires the induced vector potential to depend only on relative separations as well, so it can be written as a sum of pairwise contributions,

$$(\mathbf{A}_i)_\alpha(\{\mathbf{r}\}) = \sum_j \mathcal{A}_\alpha(\mathbf{r}_i - \mathbf{r}_j). \tag{S.19}$$

Correspondingly, the tensor structure reduces to $T_{i\alpha}^{j\beta} = \mathcal{T}_{\alpha\beta}(\mathbf{r})$. Once the dependence on the full configuration space is reduced to a dependence on the single relative coordinate $\mathbf{r}$, the original $(3N)$-dimensional Green's-function problem collapses to the ordinary three-dimensional Poisson problem. The relevant Green's function in three dimensions is therefore

$$G_3(\mathbf{r}) = \frac{1}{4\pi|\mathbf{r}|}. \tag{S.20}$$

Substituting this form into the solution above gives the pairwise vector potential

$$\mathcal{A}_\alpha(\mathbf{r}) = \frac{1}{4\pi}\int \mathrm{d}^3r'\,\frac{r_\beta - r'_\beta}{|\mathbf{r}-\mathbf{r}'|^3}\,\mathcal{T}_{\alpha\beta}(\mathbf{r}'). \tag{S.21}$$

In the case of reciprocal interactions, it is useful to specialize to the translation-invariant two-body case, as we did in the previous section for velocity-independent interactions. In this setting, the interaction between particles i and j can depend only on their relative coordinate and, because of Eq. (S.11), depend at most linearly on their velocities. We therefore introduce

$$\mathbf{r}_{ij} = \mathbf{r}_i - \mathbf{r}_j, \qquad \mathbf{v}_{ij} = \dot{\mathbf{r}}_i - \dot{\mathbf{r}}_j, \qquad \nabla \equiv \nabla_{\mathbf{r}_{ij}}. \tag{S.22}$$

We now show that reciprocity imposes a strong simplification. Although one may start from a velocity-dependent interaction written separately in terms of $\dot{\mathbf{r}}_i$ and $\dot{\mathbf{r}}_j$, Newton's third law requires that, up to an irrelevant total time derivative, the dependence on velocity enters only through the relative velocity $\mathbf{v}_{ij}$ since it requires that $\frac{\mathrm{d}}{\mathrm{d}t}(\partial_{\dot{\mathbf{r}}_i}\mathcal{U}^{\mathrm{R}}_{ij}) = -\frac{\mathrm{d}}{\mathrm{d}t}(\partial_{\dot{\mathbf{r}}_j}\mathcal{U}^{\mathrm{R}}_{ij})$.

As a result, any reciprocal pair interaction in this class can be written in the generalized-potential form

$$\mathcal{U}^{\mathrm{R}}_{ij} = \phi(\mathbf{r}_{ij}) + \mathbf{A}(\mathbf{r}_{ij}) \cdot \mathbf{v}_{ij}. \tag{S.23}$$

Here $\phi(\mathbf{r}_{ij})$ is an ordinary scalar interaction potential, while $\mathbf{A}(\mathbf{r}_{ij})$ plays the role of an effective vector interaction potential. For identical particles, exchange symmetry further requires $\phi(-\mathbf{r}) = \phi(\mathbf{r})$ and $\mathbf{A}(-\mathbf{r}) = -\mathbf{A}(\mathbf{r})$. The total reciprocal interaction is then obtained by summing this pair contribution over all pairs $\langle ij\rangle$.

This form has a simple physical interpretation. The scalar potential generates the familiar conservative interaction, whereas the vector-potential term produces the most general velocity-dependent reciprocal force that does not introduce accelerations explicitly into the equations of motion. The force exerted by particle j on particle i takes on the compact form

$$\mathbf{F}^{\mathrm{R}}_{j\to i} = -\nabla\phi(\mathbf{r}_{ij}) + \left[(\nabla\mathbf{A}(\mathbf{r}_{ij}))^T - \nabla\mathbf{A}(\mathbf{r}_{ij})\right] \cdot \mathbf{v}_{ij}. \tag{S.24}$$

Thus, the velocity-dependent part is purely antisymmetric in the velocity sector. In three dimensions, this contribution can be recognized as a Lorentz-like term, $-\mathbf{v}_{ij} \times [\nabla \times \mathbf{A}(\mathbf{r}_{ij})]$, generated by the effective magnetic field $\mathbf{B} = \nabla \times \mathbf{A}$ associated with $\mathbf{A}$.

Written in this way, reciprocity is manifest: after using $\mathbf{r}_{ji} = -\mathbf{r}_{ij}$ and $\mathbf{v}_{ji} = -\mathbf{v}_{ij}$, one immediately finds $\mathbf{F}^{\mathrm{R}}_{j\to i} = -\mathbf{F}^{\mathrm{R}}_{i\to j}$. The representation is also gauge-dependent in the usual sense: the transformation $\mathbf{A}(\mathbf{r}) \to \mathbf{A}(\mathbf{r}) + \nabla\chi(\mathbf{r})$ changes the generalized potential $\phi(\mathbf{r}_{ij})$ only by the total derivative $\mathrm{d}\chi(\mathbf{r}_{ij})/\mathrm{d}t$, and therefore leaves the equations of motion unchanged.

In summary, the most general translation-invariant reciprocal pair interaction compatible with Eq. (S.11) is fully characterized by a scalar interaction potential ϕ and a vector interaction potential $\mathbf{A}$. This provides the natural starting point for discussing velocity-dependent nonreciprocal forces below. In the following subsections, we use this structure as the building block for Hamiltonian embeddings of nonreciprocal velocity-dependent forces.

2. *A direct Lagrangian embedding for velocity-dependent nonreciprocal forces*

Velocity-dependent interactions require more care than their velocity-independent counterparts because the interaction force is no longer obtained from a spatial gradient alone, but from the full Euler–Lagrange operator, as we now discuss. Throughout this subsection, "potential" therefore refers to a *generalized* interaction potential in the Lagrangian sense. We first present the most direct extension of the embedding construction from Sec. S1 A, and then identify its regime of validity.

For a pair of particles (i, j), let the reciprocal sector be described by the generalized potential

$$U^{\mathrm{R}}(\mathbf{r}_i - \mathbf{r}_j, \dot{\mathbf{r}}_i - \dot{\mathbf{r}}_j), \tag{S.25}$$

consistent with translation invariance. For the nonreciprocal sector, we allow a directed (i.e., one-way) generalized potential

$$U^{\mathrm{NR}}_{j\to i}(\mathbf{r}_i, \mathbf{r}_j, \dot{\mathbf{r}}_i, \dot{\mathbf{r}}_j), \tag{S.26}$$

which generates the force exerted by particle j on particle i,

$$\mathbf{F}^{\mathrm{NR}}_{j\to i} = -\partial_{\mathbf{r}_i} U^{\mathrm{NR}}_{j\to i} + \frac{\mathrm{d}}{\mathrm{d}t}\,\partial_{\dot{\mathbf{r}}_i} U^{\mathrm{NR}}_{j\to i}. \tag{S.27}$$

The reciprocal force $\mathbf{F}^{\mathrm{R}}_{j\to i}$ follows analogously from U^{R}. The target equations of motion are then

$$m\ddot{\mathbf{r}}_i + \gamma\dot{\mathbf{r}}_i = \sum_{j\in\langle ij\rangle} \Big[\mathbf{F}^{\mathrm{NR}}_{j\to i}(\mathbf{r}_i, \mathbf{r}_j, \dot{\mathbf{r}}_i, \dot{\mathbf{r}}_j) + \mathbf{F}^{\mathrm{R}}_{j\to i}(\mathbf{r}_i - \mathbf{r}_j, \dot{\mathbf{r}}_i - \dot{\mathbf{r}}_j)\Big]. \tag{S.28}$$

To embed Eq. (S.28) into a reciprocal system of original and auxiliary degrees of freedom, we introduce an auxiliary copy $\mathbf{x}_i$ and consider the Bateman-like Lagrangian [1]

$$\begin{aligned}\mathcal{L} = \sum_i \left[\frac{m}{2}\dot{\mathbf{r}}_i^2 - \frac{m}{2}\dot{\mathbf{x}}_i^2 + \frac{\gamma}{2}\left(\dot{\mathbf{r}}_i\cdot\mathbf{x}_i - \mathbf{r}_i\cdot\dot{\mathbf{x}}_i\right)\right] \\ - \sum_{\langle ij\rangle} \Big[U^{\mathrm{NR}}_{j\to i}(\mathbf{r}_i, \mathbf{r}_j, \dot{\mathbf{r}}_i, \dot{\mathbf{r}}_j) + U^{\mathrm{NR}}_{i\to j}(\mathbf{r}_j, \mathbf{r}_i, \dot{\mathbf{r}}_j, \dot{\mathbf{r}}_i) - U^{\mathrm{NR}}_{j\to i}(\mathbf{x}_i, \mathbf{r}_j, \dot{\mathbf{x}}_i, \dot{\mathbf{r}}_j) - U^{\mathrm{NR}}_{i\to j}(\mathbf{x}_j, \mathbf{r}_i, \dot{\mathbf{x}}_j, \dot{\mathbf{r}}_i) \\ + U^{\mathrm{R}}(\mathbf{r}_i - \mathbf{r}_j, \dot{\mathbf{r}}_i - \dot{\mathbf{r}}_j) - U^{\mathrm{R}}(\mathbf{x}_i - \mathbf{x}_j, \dot{\mathbf{x}}_i - \dot{\mathbf{x}}_j)\Big].\end{aligned} \tag{S.29}$$

The first line contains the kinetic energy. The mixed term proportional to γ is the standard Bateman construction for linear damping [1], while the negative sign of the auxiliary kinetic energy is what preserves our mirror constraint dynamically, as shown in Eq. (S.35) below.

The Euler–Lagrange equations,

$$\frac{\mathrm{d}}{\mathrm{d}t}\frac{\partial\mathcal{L}}{\partial\dot{\mathbf{r}}_i}-\frac{\partial\mathcal{L}}{\partial\mathbf{r}_i}=0,\qquad \frac{\mathrm{d}}{\mathrm{d}t}\frac{\partial\mathcal{L}}{\partial\dot{\mathbf{x}}_i}-\frac{\partial\mathcal{L}}{\partial\mathbf{x}_i}=0, \tag{S.30}$$

yield for the original variables

$$\begin{aligned} m\ddot{\mathbf{r}}_i+\gamma\dot{\mathbf{x}}_i=&-\sum_{j\in\langle ij\rangle}\partial_{\mathbf{r}_i}\Big[U^{\mathrm{NR}}_{j\to i}(\mathbf{r}_i,\mathbf{r}_j,\dot{\mathbf{r}}_i,\dot{\mathbf{r}}_j)+U^{\mathrm{R}}(\mathbf{r}_i-\mathbf{r}_j,\dot{\mathbf{r}}_i-\dot{\mathbf{r}}_j)\Big]\\ &+\sum_{j\in\langle ij\rangle}\frac{\mathrm{d}}{\mathrm{d}t}\,\partial_{\dot{\mathbf{r}}_i}U^{\mathrm{NR}}_{j\to i}(\mathbf{r}_i,\mathbf{r}_j,\dot{\mathbf{r}}_i,\dot{\mathbf{r}}_j)\\ &+\sum_{j\in\langle ij\rangle}\frac{\mathrm{d}}{\mathrm{d}t}\,\partial_{\dot{\mathbf{r}}_i}U^{\mathrm{R}}(\mathbf{r}_i-\mathbf{r}_j,\dot{\mathbf{r}}_i-\dot{\mathbf{r}}_j)\\ &+\boldsymbol{\Xi}_i, \end{aligned} \tag{S.31}$$

where the terms not present in the original nonreciprocal equations of motion are collected into

$$\begin{aligned} \boldsymbol{\Xi}_i=&-\sum_{j\in\langle ij\rangle}\partial_{\mathbf{r}_i}\Big[U^{\mathrm{NR}}_{i\to j}(\mathbf{r}_j,\mathbf{r}_i,\dot{\mathbf{r}}_j,\dot{\mathbf{r}}_i)-U^{\mathrm{NR}}_{i\to j}(\mathbf{x}_j,\mathbf{r}_i,\dot{\mathbf{x}}_j,\dot{\mathbf{r}}_i)\Big]\\ &+\sum_{j\in\langle ij\rangle}\frac{\mathrm{d}}{\mathrm{d}t}\,\partial_{\dot{\mathbf{r}}_i}\Big[U^{\mathrm{NR}}_{i\to j}(\mathbf{r}_j,\mathbf{r}_i,\dot{\mathbf{r}}_j,\dot{\mathbf{r}}_i)-U^{\mathrm{NR}}_{i\to j}(\mathbf{x}_j,\mathbf{r}_i,\dot{\mathbf{x}}_j,\dot{\mathbf{r}}_i)\Big]. \end{aligned} \tag{S.32}$$

Accordingly, the auxiliary variables obey

$$\begin{aligned} m\ddot{\mathbf{x}}_i+\gamma\dot{\mathbf{r}}_i=&-\sum_{j\in\langle ij\rangle}\partial_{\mathbf{x}_i}\Big[U^{\mathrm{NR}}_{j\to i}(\mathbf{x}_i,\mathbf{r}_j,\dot{\mathbf{x}}_i,\dot{\mathbf{r}}_j)+U^{\mathrm{R}}(\mathbf{x}_i-\mathbf{x}_j,\dot{\mathbf{x}}_i-\dot{\mathbf{x}}_j)\Big]\\ &+\sum_{j\in\langle ij\rangle}\frac{\mathrm{d}}{\mathrm{d}t}\,\partial_{\dot{\mathbf{x}}_i}U^{\mathrm{NR}}_{j\to i}(\mathbf{x}_i,\mathbf{r}_j,\dot{\mathbf{x}}_i,\dot{\mathbf{r}}_j)\\ &+\sum_{j\in\langle ij\rangle}\frac{\mathrm{d}}{\mathrm{d}t}\,\partial_{\dot{\mathbf{x}}_i}U^{\mathrm{R}}(\mathbf{x}_i-\mathbf{x}_j,\dot{\mathbf{x}}_i-\dot{\mathbf{x}}_j). \end{aligned} \tag{S.33}$$

We now impose the mirror constraint at the initial time,

$$\mathbf{r}_i(0)=\mathbf{x}_i(0),\qquad \dot{\mathbf{r}}_i(0)=\dot{\mathbf{x}}_i(0). \tag{S.34}$$

On this manifold, $\boldsymbol{\Xi}_i\equiv 0$ identically, and Eqs. (S.31) and (S.33) collapse onto the same equation, namely Eq. (S.28) for $\mathbf{r}_i$ and, equivalently, for $\mathbf{x}_i$. It is convenient to define the difference variable $\boldsymbol{\Delta}_i\equiv\mathbf{r}_i-\mathbf{x}_i$. Subtracting the two equations of motion then gives, on the constraint manifold,

$$m\ddot{\boldsymbol{\Delta}}_i-\gamma\dot{\boldsymbol{\Delta}}_i=0. \tag{S.35}$$

Hence, the initial conditions in Eq. (S.34) imply $\boldsymbol{\Delta}_i(t)\equiv 0$ for all times: the constraint manifold is dynamically preserved, and the embedding reproduces the desired nonreciprocal dynamics.

Whenever the velocity-momentum map is non-singular, the corresponding Hamiltonian follows from a Legendre transform,

$$\mathbf{p}^{\mathbf{r}}_i=\frac{\partial\mathcal{L}}{\partial\dot{\mathbf{r}}_i},\qquad \mathbf{p}^{\mathbf{x}}_i=\frac{\partial\mathcal{L}}{\partial\dot{\mathbf{x}}_i},\qquad H=\sum_i\left(\mathbf{p}^{\mathbf{r}}_i\cdot\dot{\mathbf{r}}_i+\mathbf{p}^{\mathbf{x}}_i\cdot\dot{\mathbf{x}}_i\right)-\mathcal{L}. \tag{S.36}$$

Thus, as usual, a Hamiltonian embedding exists whenever the Lagrangian description is regular. We therefore focus on the Lagrangian formulation here.

This construction is the closest analogue of the velocity-independent embedding, but it is not fully general. It has a structural limitation: because the nonreciprocal sector is encoded through an ordinary generalized potential that depends on both the original and the auxiliary velocities, the admissible forces are restricted by Eq. (S.11). In particular, within this ansatz, the force

can be at most linear in the velocities if the equations of motion are to remain second order in time. The present embedding, therefore, captures only the subset of velocity-dependent nonreciprocal interactions that fit into a conventional generalized-potential description. Nevertheless, the similarity of this formulation of the embedding to electromagnetism and the associated gauge structure may provide new insights into the general structure of these embeddings. In Sec. S1 B 3, we introduce a more flexible construction in which the velocity dependence is attached only to the original degrees of freedom, thereby allowing for a broader class of nonreciprocal forces.

3. Velocity-dependent nonreciprocal interactions: General framework

We now extend our construction to velocity-dependent nonreciprocal interactions. While the direct Lagrangian embedding from Sec. S1 B 2 works for equations of motions that are linear in the velocity, here we present a more flexible formulation that accommodates a broader class of velocity-dependent forces.

Consider the Lagrangian for the doubled system:

$$\begin{aligned}\mathcal{L} = &\sum_i \left[\frac{m}{2}\dot{\mathbf{r}}_i^2 - \frac{m}{2}\dot{\mathbf{x}}_i^2 + \frac{\gamma}{2}\left(\dot{\mathbf{r}}_i\cdot\mathbf{x}_i - \mathbf{r}_i\cdot\dot{\mathbf{x}}_i\right)\right] \\ &- \sum_{\langle ij\rangle}\Big[U^{\mathrm{NR}}_{j\to i}(\mathbf{r}_i,\mathbf{r}_j,\dot{\mathbf{r}}_i,\dot{\mathbf{r}}_j) + U^{\mathrm{NR}}_{i\to j}(\mathbf{r}_j,\mathbf{r}_i,\dot{\mathbf{r}}_j,\dot{\mathbf{r}}_i) \\ &- U^{\mathrm{NR}}_{j\to i}(\mathbf{x}_i,\mathbf{r}_j,\dot{\mathbf{r}}_i,\dot{\mathbf{r}}_j) - U^{\mathrm{NR}}_{i\to j}(\mathbf{x}_j,\mathbf{r}_i,\dot{\mathbf{r}}_j,\dot{\mathbf{r}}_i) \\ &+ U^{\mathrm{R}}(\mathbf{r}_i-\mathbf{r}_j,\dot{\mathbf{r}}_i-\dot{\mathbf{r}}_j) - U^{\mathrm{R}}(\mathbf{x}_i-\mathbf{x}_j,\dot{\mathbf{x}}_i-\dot{\mathbf{x}}_j)\Big]. \end{aligned} \tag{S.37}$$

The key difference from Eq. (S.29) is that the velocity-dependent nonreciprocal potential couples only to the original degrees of freedom $\dot{\mathbf{r}}_i$, and not to the auxiliary ones $\dot{\mathbf{x}}_i$. This modification enables the embedding to capture velocity-dependent forces that are not restricted to being linear in velocity.

From the Euler-Lagrange equations, the original degrees of freedom satisfy

$$\begin{aligned} m\ddot{\mathbf{r}}_i + \gamma\dot{\mathbf{x}}_i = &- \sum_{j\in\langle ij\rangle} \partial_{\mathbf{r}_i}\Big[U^{\mathrm{NR}}_{j\to i}(\mathbf{r}_i,\mathbf{r}_j,\dot{\mathbf{r}}_i,\dot{\mathbf{r}}_j) + U^{\mathrm{R}}(\mathbf{r}_i-\mathbf{r}_j,\dot{\mathbf{r}}_i-\dot{\mathbf{r}}_j)\Big] \\ &+ \sum_{j\in\langle ij\rangle}\frac{\mathrm{d}}{\mathrm{d}t}\partial_{\dot{\mathbf{r}}_i}U^{\mathrm{R}}(\mathbf{r}_i-\mathbf{r}_j,\dot{\mathbf{r}}_i-\dot{\mathbf{r}}_j) \\ &+ \boldsymbol{\Xi}_i, \end{aligned} \tag{S.38}$$

where the unwanted correction terms are collected in

$$\begin{aligned} \boldsymbol{\Xi}_i = &- \sum_{j\in\langle ij\rangle}\partial_{\mathbf{r}_i}\Big[U^{\mathrm{NR}}_{j\to i}(\mathbf{r}_i,\mathbf{r}_j,\dot{\mathbf{r}}_i,\dot{\mathbf{r}}_j) - U^{\mathrm{NR}}_{j\to i}(\mathbf{x}_i,\mathbf{r}_j,\dot{\mathbf{r}}_i,\dot{\mathbf{r}}_j)\Big] \\ &+ \sum_{j\in\langle ij\rangle}\frac{\mathrm{d}}{\mathrm{d}t}\partial_{\dot{\mathbf{r}}_i}\Big[U^{\mathrm{NR}}_{j\to i}(\mathbf{r}_i,\mathbf{r}_j,\dot{\mathbf{r}}_i,\dot{\mathbf{r}}_j) + U^{\mathrm{NR}}_{i\to j}(\mathbf{r}_j,\mathbf{r}_i,\dot{\mathbf{r}}_j,\dot{\mathbf{r}}_i) \\ &- U^{\mathrm{NR}}_{j\to i}(\mathbf{x}_i,\mathbf{r}_j,\dot{\mathbf{r}}_i,\dot{\mathbf{r}}_j) - U^{\mathrm{NR}}_{i\to j}(\mathbf{x}_j,\mathbf{r}_i,\dot{\mathbf{r}}_j,\dot{\mathbf{r}}_i)\Big]. \end{aligned} \tag{S.39}$$

The auxiliary degrees of freedom evolve according to

$$\begin{aligned} m\ddot{\mathbf{x}}_i + \gamma\dot{\mathbf{r}}_i = &- \sum_{j\in\langle ij\rangle}\partial_{\mathbf{x}_i}\Big[U^{\mathrm{NR}}_{j\to i}(\mathbf{x}_i,\mathbf{r}_j,\dot{\mathbf{r}}_i,\dot{\mathbf{r}}_j) + U^{\mathrm{R}}(\mathbf{x}_i-\mathbf{x}_j,\dot{\mathbf{x}}_i-\dot{\mathbf{x}}_j)\Big] \\ &+ \sum_{j\in\langle ij\rangle}\frac{\mathrm{d}}{\mathrm{d}t}\partial_{\dot{\mathbf{x}}_i}U^{\mathrm{R}}(\mathbf{x}_i-\mathbf{x}_j,\dot{\mathbf{x}}_i-\dot{\mathbf{x}}_j). \end{aligned} \tag{S.40}$$

We impose the mirror constraint at the initial time:

$$\mathbf{r}_i(0) = \mathbf{x}_i(0), \qquad \dot{\mathbf{r}}_i(0) = \dot{\mathbf{x}}_i(0). \tag{S.41}$$

As expected, on the constraint manifold, $\boldsymbol{\Xi}_i \equiv 0$ vanishes identically, and both the original and auxiliary equations reduce to the same dynamical equations. Once again, the constraint defines a closed submanifold: differentiating the constraint yields

$$\frac{\mathrm{d}}{\mathrm{d}t}\left[\mathbf{r}_i(t) - \mathbf{x}_i(t)\right]\Big|_{\text{constr.}} = 0, \qquad \frac{\mathrm{d}}{\mathrm{d}t}\left[\dot{\mathbf{r}}_i(t) - \dot{\mathbf{x}}_i(t)\right]\Big|_{\text{constr.}} = 0. \tag{S.42}$$

This ensures that trajectories satisfying the constraint at $t = 0$ remain on the constraint manifold for all times.

The key advantage of this formulation is that the velocity dependence of the nonreciprocal potential U^{NR} is decoupled from the auxiliary degrees of freedom. Consequently, the embedding can accommodate nonreciprocal forces with polynomial or even transcendental velocity dependence, not merely linear terms. The force exerted by particle j on particle i is given by

$$\mathbf{F}^{\mathrm{NR}}_{j\to i}(\mathbf{r}_i, \mathbf{r}_j, \dot{\mathbf{r}}_i, \dot{\mathbf{r}}_j) = -\partial_{\mathbf{r}_i} U^{\mathrm{NR}}_{j\to i}(\mathbf{r}_i, \mathbf{r}_j, \dot{\mathbf{r}}_i, \dot{\mathbf{r}}_j) \tag{S.43}$$

which is not restricted to linear velocity dependence. This demonstrates the generality and flexibility of the embedding framework for velocity-dependent nonreciprocal interactions.

4. Example: Lorentz-like forces

As a concrete example of velocity-dependent nonreciprocal interactions, consider pairwise Lorentz-like forces of the form

$$m\ddot{\mathbf{r}}_i + \gamma\dot{\mathbf{r}}_i = \sum_{j\in\langle ij\rangle} \mathbf{F}^{\mathrm{NR}}_{j\to i}, \qquad \mathbf{F}^{\mathrm{NR}}_{j\to i} = \dot{\mathbf{r}}_i \times \mathbf{B}_{j\to i}(\mathbf{r}_i, \mathbf{r}_j), \tag{S.44}$$

with

$$\mathbf{B}_{j\to i}(\mathbf{r}_i, \mathbf{r}_j) = \nabla_{\mathbf{r}_i} \times \mathbf{A}_{j\to i}(\mathbf{r}_i, \mathbf{r}_j). \tag{S.45}$$

In general, $\mathbf{A}_{j\to i} \neq \mathbf{A}_{i\to j}$, so the force exerted by particle j on particle i need not be the opposite to the force exerted by i on j; this interaction is therefore nonreciprocal. The overdamped limit is recovered by setting $m = 0$.

This example is useful because it allows us to make explicit the difference between the two velocity-dependent embeddings introduced above.

Direct generalized-potential embedding. The construction of Sec. S1 B 2 applies provided the Lorentz-like force can be generated from a generalized (one-way) potential. A sufficient condition is that there exists a companion vector field $\tilde{\mathbf{A}}_{j\to i}$ such that

$$\left(\partial_{\mathbf{r}_i} \tilde{\mathbf{A}}_{j\to i}\right)^T = \partial_{\mathbf{r}_j} \mathbf{A}_{j\to i}. \tag{S.46}$$

Then the directed generalized potential

$$U^{\mathrm{NR}}_{j\to i} = -\mathbf{A}_{j\to i}(\mathbf{r}_i, \mathbf{r}_j) \cdot \dot{\mathbf{r}}_i - \tilde{\mathbf{A}}_{j\to i}(\mathbf{r}_i, \mathbf{r}_j) \cdot \dot{\mathbf{r}}_j \tag{S.47}$$

satisfies

$$-\partial_{\mathbf{r}_i} U^{\mathrm{NR}}_{j\to i} + \frac{\mathrm{d}}{\mathrm{d}t}\partial_{\dot{\mathbf{r}}_i} U^{\mathrm{NR}}_{j\to i} = \dot{\mathbf{r}}_i \times \left(\nabla_{\mathbf{r}_i} \times \mathbf{A}_{j\to i}\right), \tag{S.48}$$

where Eq. (S.46) ensures the cancellation of all terms proportional to $\dot{\mathbf{r}}_j$. Substituting Eq. (S.47) into the Lagrangian embedding of Sec. S1 B 2, Eq. (S.29), yields an embedding of the form in Eq. (S.44). More explicitly, if we set the reciprocal sector to zero for simplicity, the embedding Lagrangian becomes

$$\begin{aligned}\mathcal{L} = &\sum_i \left[\frac{m}{2}\dot{\mathbf{r}}_i^2 - \frac{m}{2}\dot{\mathbf{x}}_i^2 + \frac{\gamma}{2}\left(\dot{\mathbf{r}}_i \cdot \mathbf{x}_i - \mathbf{r}_i \cdot \dot{\mathbf{x}}_i\right)\right] \\ &+ \sum_{\langle ij\rangle} \Big[\mathbf{A}_{j\to i}(\mathbf{r}_i, \mathbf{r}_j) \cdot \dot{\mathbf{r}}_i + \tilde{\mathbf{A}}_{j\to i}(\mathbf{r}_i, \mathbf{r}_j) \cdot \dot{\mathbf{r}}_j + \mathbf{A}_{i\to j}(\mathbf{r}_j, \mathbf{r}_i) \cdot \dot{\mathbf{r}}_j + \tilde{\mathbf{A}}_{i\to j}(\mathbf{r}_j, \mathbf{r}_i) \cdot \dot{\mathbf{r}}_i \\ &\qquad - \mathbf{A}_{j\to i}(\mathbf{x}_i, \mathbf{r}_j) \cdot \dot{\mathbf{x}}_i - \tilde{\mathbf{A}}_{j\to i}(\mathbf{x}_i, \mathbf{r}_j) \cdot \dot{\mathbf{r}}_j - \mathbf{A}_{i\to j}(\mathbf{x}_j, \mathbf{r}_i) \cdot \dot{\mathbf{x}}_j - \tilde{\mathbf{A}}_{i\to j}(\mathbf{x}_j, \mathbf{r}_i) \cdot \dot{\mathbf{r}}_i\Big].\end{aligned} \tag{S.49}$$

The Euler–Lagrange equations derived from Eq. (S.49) reduce to Eq. (S.44) on the constraint manifold. With the mirror constraint

$$\mathbf{r}_i(0) = \mathbf{x}_i(0), \qquad \dot{\mathbf{r}}_i(0) = \dot{\mathbf{x}}_i(0), \tag{S.50}$$

the two copies remain locked for all times, and both follow the same Lorentz-like dynamics.

General embedding. If no field $\tilde{\mathbf{A}}_{j\to i}$ satisfying Eq. (S.46) exists, the direct route above is not viable. One can nevertheless use the broader construction of Sec. S1 B 3, which does not require the Lorentz-like force itself to arise from a generalized potential. Defining

$$\mathbf{G}_{j\to i}(\mathbf{r}_i, \mathbf{r}_j, \dot{\mathbf{r}}_i) = -\dot{\mathbf{r}}_i \times \mathbf{B}_{j\to i}(\mathbf{r}_i, \mathbf{r}_j), \tag{S.51}$$

we introduce the embedding Lagrangian

$$\begin{aligned}\mathcal{L} = &\sum_i \left[\frac{m}{2}\dot{\mathbf{r}}_i^2 - \frac{m}{2}\dot{\mathbf{x}}_i^2 + \frac{\gamma}{2}\left(\dot{\mathbf{r}}_i \cdot \mathbf{x}_i - \mathbf{r}_i \cdot \dot{\mathbf{x}}_i\right)\right] \\ &- \sum_{\langle ij\rangle} \Big[\mathbf{G}_{j\to i}(\mathbf{r}_i, \mathbf{r}_j, \dot{\mathbf{r}}_i) \cdot (\mathbf{r}_i - \mathbf{x}_i) + \mathbf{G}_{i\to j}(\mathbf{r}_j, \mathbf{r}_i, \dot{\mathbf{r}}_j) \cdot (\mathbf{r}_j - \mathbf{x}_j)\Big].\end{aligned} \tag{S.52}$$

Away from the constraint manifold, the equations of motion for $\mathbf{r}_i$ and $\mathbf{x}_i$ contain extra terms proportional to $\mathbf{r}_i - \mathbf{x}_i$ and $\dot{\mathbf{r}}_i - \dot{\mathbf{x}}_i$. On the mirror-constraint manifold, these terms vanish identically. Hence, for initial conditions satisfying

$$\mathbf{r}_i(0) = \mathbf{x}_i(0), \qquad \dot{\mathbf{r}}_i(0) = \dot{\mathbf{x}}_i(0), \tag{S.53}$$

the constraint is dynamically preserved and the reduced dynamics become

$$m\ddot{\mathbf{r}}_i + \gamma\dot{\mathbf{r}}_i = \sum_{j\in\langle ij\rangle} \dot{\mathbf{r}}_i \times \mathbf{B}_{j\to i}(\mathbf{r}_i, \mathbf{r}_j). \tag{S.54}$$

In summary, Lorentz-like forces cleanly distinguish the scope of the two velocity-dependent embeddings. When the integrability condition (S.46) holds, the interaction admits a bona fide generalized-potential representation and the direct construction of Sec. S1 B 2 is sufficient. When it does not, the more general framework of Sec. S1 B 3 still provides a consistent embedding, with the mirror constraint selecting the physical nonreciprocal dynamics.

S2. EXAMPLES OF NONRECIPROCAL SYSTEMS TREATED WITHIN THE HAMILTONIAN EMBEDDING

Below, we discuss concrete examples of this general Hamiltonian embedding for nonreciprocal interactions, see Fig. S1.

A. XY spins with vision cone interactions

A first illustrative example is the vision-cone XY spins, $\mathbf{S}_i = (\cos\theta_i, \sin\theta_i)$, on a square lattice discussed in the Main Text. Here, the nonreciprocal equations of motion take the form [cf. Eq. (**??**) and Fig. S1(a)]:

$$\dot{\theta}_i = -\sum_{j\in\langle ij\rangle} J_{ij}(\theta_i)\sin\big(\theta_i - \theta_j\big) + \sqrt{2T}\eta_i. \tag{S.55}$$

To construct the Hamiltonian, we introduce the auxiliary spins $\mathbf{a}_i$ parametrized by the angle φ_i. Proceeding as explained in the Main Text, we obtain:

$$\begin{aligned}H &= -\sum_{\langle ij\rangle}\left[J_{ij}(\theta_i)(\mathbf{S}_i\cdot\mathbf{S}_j + \mathbf{a}_i\cdot\mathbf{S}_j) + J_{ji}(\theta_j)(\mathbf{S}_j\cdot\mathbf{S}_i + \mathbf{a}_j\cdot\mathbf{S}_i)\right], \\ &= -\sum_{\langle ij\rangle}\left\{J_{ij}(\theta_i)[\cos\big(\theta_i-\theta_j\big) + \cos\big(\varphi_i-\theta_j\big)] + J_{ji}(\theta_j)[\cos\big(\theta_j-\theta_i\big) + \cos\big(\varphi_j-\theta_i\big)]\right\}.\end{aligned} \tag{S.56}$$

The equations of motion are given in Eq. (**??**), ignoring the noise term. The origin and technical handling of the constraint are discussed in detail in Sec. S3.

As we now demonstrate, this way of constructing the Hamiltonian is generic and can be applied to other models with nonreciprocal interactions.

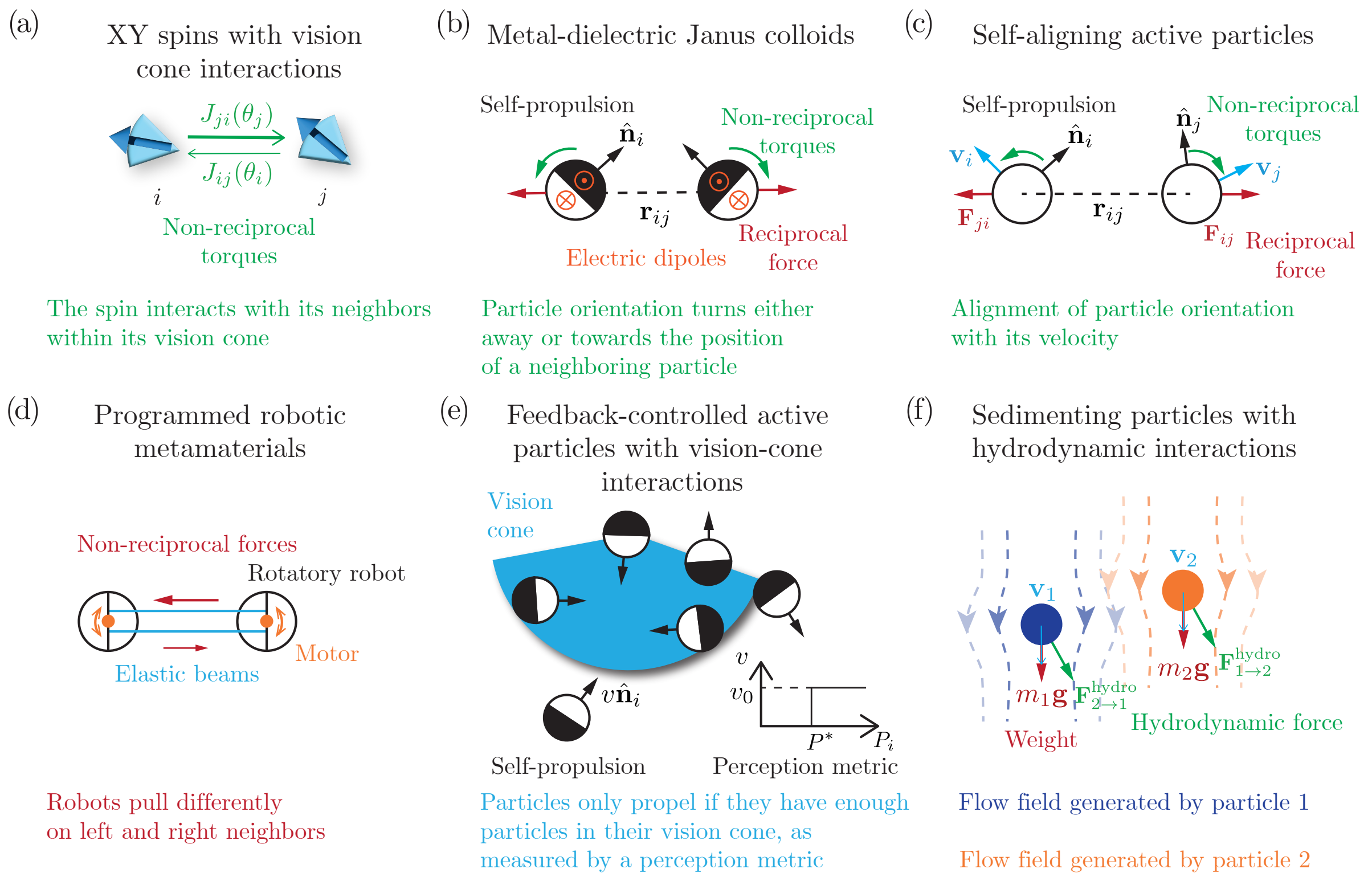

FIG. S1. Schematic representation of various models exhibiting nonreciprocal equations of motion. (a) XY spins with vision-cone interactions (Sec. S2 A) experiences torques from the neighboring spins within its vision cone. (b) Metal-dielectric self-propelled Janus colloidal particles (Sec. S2 B) experience self-propulsion, reciprocal repulsive forces and nonreciprocal torques. (c) Self-aligning active particles (Sec. S2 C) interact via reciprocal repulsive forces and exhibit self-propulsion. The nonreciprocal torques cause each particle to rotate toward its velocity direction. (d) Programmed robotic metamaterials (Sec. S2 D) consist of robotic units connected by springs that exert asymmetric forces on the left and right sides. (e) Feedback-controlled active particles (Sec. S2 E) interact via reciprocal forces and self-propel when a sufficient number of neighboring particles enter their vision cone. (f) Sedimenting particles settling through a viscous fluid under gravity (Sec. S2 F) affect each other by generating flow fields. The resulting interactions are intrinsically nonreciprocal [2, 3].

B. Metal-dielectric Janus particles

The next example is that of metal-dielectric self-propelled Janus colloidal particles. These particles are silica spheres coated with titanium on one hemisphere. Suspended in water, they sediment and form a monolayer on a conductive coverslip, separated from another conductive coverslip, which together form a capacitor [4–6]. The particles are driven by an AC electric field produced by a voltage between the conductive coverslips. The electric field induces dipoles in each of the particles' hemispheres, which leads both to self-propulsion as well as to electrostatic interactions between the particles. The interactions consist of a repulsive force and a torque that tends to make the particles turn either towards or away from one another [5, 6]. At low area fractions and self-propulsion speeds, the system behaves as an isotropic active gas. At higher area fractions and speeds, the system either undergoes phase separation when particles turn towards each other [5] or transitions to a polar-ordered flock when particles turn away from one another [6].

To explain these phenomena, previous work proposed a two-dimensional microscopic model based on the dipolar interactions between the hemispheres of the particles (Fig. S1b) [5]. The interaction torques, which tend to rotate a particle either away or towards the position of a second particle, are intrinsically nonreciprocal; they do not have a reciprocal limit [5, 6]. The position and orientation of particle i are denoted by $\mathbf{r}_i$ and $\hat{\mathbf{n}}_i$, respectively. The equations of motion are given by

$$\frac{\mathrm{d}\mathbf{r}_i}{\mathrm{d}t} = v_0\hat{\mathbf{n}}_i + \sum_{j\neq i}\frac{\mathbf{F}_{ji}}{\gamma_\mathrm{t}} +_i^{\mathrm{t}},$$
$$\frac{\mathrm{d}\hat{\mathbf{n}}_i}{\mathrm{d}t} = \sum_{j\neq i}\frac{\mathbf{\Gamma}_{ji}}{\gamma_\mathrm{r}} + \hat{\mathbf{n}}_i\times\eta_i^{\mathrm{r}}. \tag{S.57}$$

Here, η_i^{t} and η_i^{r} are translational and rotational Gaussian white noises with zero mean and with $\langle\eta_{i,\alpha}^{\mathrm{t}}(t)\eta_{j,\beta}^{\mathrm{t}}(t')\rangle = 2D_\mathrm{t}\delta_{ij}\delta_{\alpha\beta}\delta(t-$

$t')$ and $\langle \eta^{\mathrm{r}}_{i,z}(t)\eta^{\mathrm{r}}_{j,z}(t')\rangle = 2D_{\mathrm{r}}\delta_{ij}\delta(t-t')$, where D_{t} and D_{r} are the translational and rotational diffusion coefficients of the particles. Here, Latin indices indicate particles, whereas Greek indices indicate spatial components. Note that the rotational noise has components only along the $\hat{\mathbf{z}}$ axis so that the particle orientation $\hat{\mathbf{n}}_i$ remains on the xy plane.

The particle positions change due to self-propulsion at a speed v_0, and due to the reciprocal repulsive force $\mathbf{F}_{ji}$ exerted by particle j on particle i, with γ_{t} being the translational friction coefficient. This interaction force can be derived from an interaction potential $U(|\mathbf{r}_i - \mathbf{r}_j|)$ between particles i and j. In addition, $\mathbf{\Gamma}_{ji}$ denotes the interaction torque, which is intrinsically nonreciprocal, $\mathbf{\Gamma}_{ji} \neq -\mathbf{\Gamma}_{ij}$, and γ_{r} is the associated rotational friction coefficient.

Assuming turn-towards torques, the expressions for the force $\mathbf{F}_{ji}$ and the torque $\mathbf{\Gamma}_{ji}$ are given by [5]

$$\begin{aligned}\mathbf{F}_{ji} &= \frac{3}{4\pi\epsilon}\frac{(d_{\mathrm{h}}+d_{\mathrm{t}})^2}{r_{ji}^4}\exp\left(-\frac{r_{ji}}{\lambda}\right)\hat{\mathbf{r}}_{ji},\\ \mathbf{\Gamma}_{ji} &= \frac{3\ell}{4\pi\epsilon}\frac{d_{\mathrm{h}}^2-d_{\mathrm{t}}^2}{r_{ji}^4}\exp\left(-\frac{r_{ji}}{\lambda}\right)\hat{\mathbf{n}}_i\times\hat{\mathbf{r}}_{ji} = \tau_{ji}\times\hat{\mathbf{n}}_i,\end{aligned} \tag{S.58}$$

where ϵ is the dielectric permittivity of the solvent, and d_{h} and d_{t} are the effective dipole strengths of the head and tail hemispheres. Here, to shorten the notation, we have rewritten the interaction torque in terms of the quantity

$$\tau_{ji} \equiv -\frac{3\ell}{4\pi\epsilon}\frac{d_{\mathrm{h}}^2-d_{\mathrm{t}}^2}{r_{ji}^4}\exp\left(-\frac{r_{ji}}{\lambda}\right)\hat{\mathbf{r}}_{ji}. \tag{S.59}$$

Here $\mathbf{r}_{ji} = \mathbf{r}_i - \mathbf{r}_j$ is the distance vector with its length and direction given by $r_{ji} = |\mathbf{r}_{ji}|$ and $\hat{\mathbf{r}}_{ji} = \mathbf{r}_{ji}/r_{ji}$, respectively.

So far, we discussed all forces and torques. We now introduce the auxiliary particles with position and orientation denoted as $\mathbf{x}_i$ and $\hat{\mathbf{m}}_i$. The Hamiltonian embedding is given by

$$H = \sum_i -v_0\hat{\mathbf{n}}_i\cdot(\mathbf{r}_i+\mathbf{x}_i) + \sum_{i\neq j}\left[\frac{U(|\mathbf{r}_i-\mathbf{r}_j|)}{\gamma_{\mathrm{t}}} - \frac{U(|\mathbf{x}_i-\mathbf{x}_j|)}{\gamma_{\mathrm{t}}} + \frac{\tau_{ji}}{\gamma_{\mathrm{r}}}\cdot(\hat{\mathbf{n}}_i+\hat{\mathbf{m}}_i) + \frac{\tau_{ij}}{\gamma_{\mathrm{r}}}\cdot\left(\hat{\mathbf{n}}_j+\hat{\mathbf{m}}_j\right)\right]. \tag{S.60}$$

We can derive the corresponding equations of motion from the Hamiltonian description. The translational motion of the particles is governed by

$$\frac{\mathrm{d}\mathbf{r}_i}{\mathrm{d}t} = -\frac{\partial H}{\partial\mathbf{r}_i} = v_0\hat{\mathbf{n}}_i - \sum_{j\neq i}\left[\frac{1}{\gamma_{\mathrm{t}}}\frac{\partial U(|\mathbf{r}_i-\mathbf{r}_j|)}{\partial\mathbf{r}_i} - \frac{\partial}{\partial\mathbf{r}_i}\frac{\tau_{ij}}{\gamma_{\mathrm{r}}}\cdot(\hat{\mathbf{n}}_i+\hat{\mathbf{m}}_i) - \frac{\partial}{\partial\mathbf{r}_i}\frac{\tau_{ji}}{\gamma_{\mathrm{r}}}\cdot\left(\hat{\mathbf{n}}_j+\hat{\mathbf{m}}_j\right)\right], \tag{S.61}$$

and the orientational motion of the particle follows

$$\frac{\mathrm{d}\hat{\mathbf{n}}_i}{\mathrm{d}t} = \frac{\partial H}{\partial\hat{\mathbf{n}}_i}\times\hat{\mathbf{n}}_i = -v_0\left(\mathbf{r}_i+\mathbf{x}_i\right)\times\hat{\mathbf{n}}_i + \sum_{j\neq i}\frac{\tau_{ji}}{\gamma_{\mathrm{r}}}\times\hat{\mathbf{n}}_i, \tag{S.62}$$

where we omit the corresponding equations for $\mathbf{x}_i$ and $\mathbf{m}_i$.

Applying the constraints

$$\begin{aligned}\mathbf{r}_i(t)+\mathbf{x}_i(t) &= 0,\\ \hat{\mathbf{n}}_i(t)+\hat{\mathbf{m}}_i(t) &= 0\end{aligned} \tag{S.63}$$

to the equations of motion yields

$$\begin{aligned}\frac{\mathrm{d}\mathbf{r}_i}{\mathrm{d}t} &= v_0\hat{\mathbf{n}}_i + \sum_{j\neq i}\frac{\mathbf{F}_{ji}}{\gamma_{\mathrm{t}}},\\ \frac{\mathrm{d}\hat{\mathbf{n}}_i}{\mathrm{d}t} &= \sum_{j\neq i}\frac{\mathbf{\Gamma}_{ji}}{\gamma_{\mathrm{r}}},\end{aligned} \tag{S.64}$$

which is the same as Eq. (S.57), except for the noise terms $\eta_i^{\mathrm{t}}, \eta_i^{\mathrm{r}}$.

C. Self-aligning active particles

Next we study systems of self-aligning active particles — a model that has been proposed for cells, vibrated disks, and walker robots [7–10]. The interplay of self-alignment and central forces between the particles has been found to give rise to flocking or collective oscillations, depending on whether the system is confined, and whether it is liquid or solid [7–11].

Self-aligning active particles, with positions $\mathbf{r}_i$ and orientations $\hat{\mathbf{n}}_i$, tend to align their orientation towards their velocity. They interact via central forces, arising for example from hard-disk collisions or elastic springs, resulting in nonreciprocal effective interactions. The equations of motion are given by [9, 10]

$$\frac{\mathrm{d}\mathbf{r}_i}{\mathrm{d}t} = v_0\hat{\mathbf{n}}_i + \sum_{j\neq i}\frac{\mathbf{F}_{ji}}{\gamma_\mathrm{t}} + \eta_i^\mathrm{t},$$
$$\frac{\mathrm{d}\hat{\mathbf{n}}_i}{\mathrm{d}t} = \frac{1}{\ell_\mathrm{a}}\left(\hat{\mathbf{n}}_i \times \dot{\mathbf{r}}_i\right)\times\hat{\mathbf{n}}_i + \hat{\mathbf{n}}_i \times \eta_i^\mathrm{r}. \tag{S.65}$$

Here, v_0 denotes the self-propulsion speed, γ_t is the translational friction coefficient, and $\mathbf{F}_{ji} = -\mathbf{F}_{ij}$ represents the reciprocal repulsive force that particle i feels from particle j. Such interaction force can be derived from a potential $U(|\mathbf{r}_i - \mathbf{r}_j|)$ between particles i and j. The second equation expresses that the orientation $\hat{\mathbf{n}}_i$ of a particle rotates towards its velocity $\dot{\mathbf{r}}_i$ over an alignment length ℓ_a. A schematic illustration is presented in Fig. S1c. Ignoring the noise terms η_i^t and η_i^r, we substitute the velocity in the second equation using the first equation, thereby obtaining the following expression for the equations of motion

$$\frac{\mathrm{d}\hat{\mathbf{n}}_i}{\mathrm{d}t} = \frac{1}{\ell_\mathrm{a}\gamma_\mathrm{t}}\sum_{j\neq i}\left(\hat{\mathbf{n}}_i\times\mathbf{F}_{ji}\right)\times\hat{\mathbf{n}}_i. \tag{S.66}$$

This expression shows that the self-aligning torque tends to align the particle orientation with the interaction force. Such a torque is intrinsically nonreciprocal because, in general, $\left[\hat{\mathbf{n}}_i\times\mathbf{F}_{ji}\right]\times\hat{\mathbf{n}}_i \neq -\left[\hat{\mathbf{n}}_j\times\mathbf{F}_{ij}\right]\times\hat{\mathbf{n}}_j$ as $\hat{\mathbf{n}}_i$, and $\hat{\mathbf{n}}_j$ are in general different.

We now introduce auxiliary particles with position and orientation denoted by $\mathbf{x}_i$ and $\hat{\mathbf{m}}_i$. The Hamiltonian embedding is given by

$$H = -\sum_i v_0\hat{\mathbf{n}}_i\cdot(\mathbf{r}_i+\mathbf{x}_i)$$
$$+\frac{1}{\gamma_\mathrm{t}}\sum_{i\neq j}\left\{U(|\mathbf{r}_i-\mathbf{r}_j|) - U(|\mathbf{x}_i-\mathbf{x}_j|) + \frac{1}{\ell_\mathrm{a}}\left[\hat{\mathbf{n}}_i\times\mathbf{F}_{ji}\right]\cdot(\hat{\mathbf{n}}_i+\hat{\mathbf{m}}_i) + \frac{1}{\ell_\mathrm{a}}\left[\hat{\mathbf{n}}_j\times\mathbf{F}_{ij}\right]\cdot(\hat{\mathbf{n}}_j+\hat{\mathbf{m}}_j)\right\}. \tag{S.67}$$

We can now derive the corresponding equations of motion from this Hamiltonian description. The translational motion of the particles obeys

$$\frac{\mathrm{d}\mathbf{r}_i}{\mathrm{d}t} = -\frac{\partial H}{\partial \mathbf{r}_i}$$
$$= v_0\hat{\mathbf{n}}_i + \sum_{j\neq i}\left\{-\frac{1}{\gamma_\mathrm{t}}\frac{\partial U(|\mathbf{r}_i-\mathbf{r}_j|)}{\partial\mathbf{r}_i} + \frac{1}{\ell_\mathrm{a}\gamma_\mathrm{t}}\frac{\partial\mathbf{F}_{ij}}{\partial\mathbf{r}_i}\left[(\hat{\mathbf{n}}_j+\hat{\mathbf{m}}_j)\times\hat{\mathbf{n}}_j\right] + \frac{1}{\ell_\mathrm{a}\gamma_\mathrm{t}}\frac{\partial\mathbf{F}_{ji}}{\partial\mathbf{r}_i}\left[(\hat{\mathbf{n}}_i+\hat{\mathbf{m}}_i)\times\hat{\mathbf{n}}_i\right]\right\}, \tag{S.68}$$

and the orientational motion of the particle follows

$$\frac{\mathrm{d}\hat{\mathbf{n}}_i}{\mathrm{d}t} = \frac{\partial H}{\partial\hat{\mathbf{n}}_i}\times\hat{\mathbf{n}}_i$$
$$= -v_0\left(\mathbf{r}_i+\mathbf{x}_i\right)\times\hat{\mathbf{n}}_i + \frac{1}{\ell_\mathrm{a}\gamma_\mathrm{t}}\sum_{j\neq i}\left\{\left[\hat{\mathbf{n}}_i\times\mathbf{F}_{ji}\right]\times\hat{\mathbf{n}}_i + \left[\mathbf{F}_{ji}\times(\hat{\mathbf{n}}_i+\hat{\mathbf{m}}_i)\right]\times\hat{\mathbf{n}}_i\right\}. \tag{S.69}$$

Finally, the constraints read as

$$\mathbf{r}_i(t)+\mathbf{x}_i(t) = 0,$$
$$\hat{\mathbf{n}}_i(t)+\hat{\mathbf{m}}_i(t) = 0. \tag{S.70}$$

Applying the constraints to the equations of motion yields

$$\frac{\mathrm{d}\mathbf{r}_i}{\mathrm{d}t} = v_0\hat{\mathbf{n}}_i + \frac{1}{\gamma_\mathrm{t}}\sum_{j\neq i}\mathbf{F}_{ij},$$
$$\frac{\mathrm{d}\hat{\mathbf{n}}_i}{\mathrm{d}t} = \frac{1}{\ell_\mathrm{a}\gamma_\mathrm{t}}\sum_{j\neq i}\left(\hat{\mathbf{n}}_i\times\mathbf{F}_{ij}\right)\times\hat{\mathbf{n}}_i, \tag{S.71}$$

which is equivalent to Eq. (S.65), except for the noise terms.

D. Programmed robotic metamaterials

Let us now look at mechanical metamaterials made of robots whose interactions are programmed to break reciprocity [12, 13]. In such materials, nonreciprocal interactions give rise to spatially asymmetric standing waves and unidirectionally amplified propagating waves, realizing the mechanical analogue of the non-Hermitian skin effect [14, 15]. Specifically, the robots are programmed to turn more (less) depending on how their left (right) neighbors turn, as schematized in Fig. S1d. The dynamics can be mapped onto the following nonreciprocal discrete wave equation, which arises from a mass-spring model in which the forces exerted by the springs on the left and right sides differ [12]:

$$\ddot{u}_i = -k\,(1+\varepsilon)\,(u_{i-1}-u_i) - k\,(1-\varepsilon)\,(u_{i+1}-u_i)\,. \tag{S.72}$$

Here u_i denotes the displacement of robot i; $k\,(1+\varepsilon)$ and $k\,(1-\varepsilon)$ represent the stiffnesses of coupling springs from left to right and from right to left, respectively, with the dimensionless quantity ε quantifying nonreciprocity. For convenience, we have set the mass to unity.

We now introduce the conjugate momentum p_i^u for u_i, along with the auxiliary degrees of freedom p_i^w and w_i together with the Poisson bracket relations, $\left\{u_i, p_j^u\right\} = \delta_{ij}$, and $\left\{w_i, p_j^w\right\} = \delta_{ij}$. A Hamiltonian can then be constructed as follows:

$$H = \sum_i \left[\frac{\left(p_i^u\right)^2}{2} - \frac{\left(p_i^w\right)^2}{2} + k\,(1+\varepsilon)\,(u_{i-1}-u_i)\,(u_i+w_i) + k\,(1-\varepsilon)\,(u_{i+1}-u_i)\,(u_i+w_i)\right]. \tag{S.73}$$

We observe that the kinetic energy of the auxiliary particles is negative. Such a negative kinetic energy term in the auxiliary system is necessary to enforce our constraints; a more detailed explanation will be provided in Sec. S3 B.

We can derive the corresponding equations of motion from the Hamiltonian description

$$\begin{aligned}
\dot{u}_i &= \{u_i, H\} = p_i^u,\\
\dot{w}_i &= \{w_i, H\} = -p_i^w,\\
\dot{p}_i^u &= \{p_i^u, H\} = -k\,(1+\varepsilon)\,(u_{i-1}-u_i) - k\,(1-\varepsilon)\,(u_{i+1}-u_i)\\
&\quad + k\,(1+\varepsilon)\,(u_i+w_i) + k\,(1-\varepsilon)\,(u_i+w_i) - k\,(1+\varepsilon)\,(u_{i+1}+w_{i+1}) - k\,(1-\varepsilon)\,(u_{i-1}+w_{i-1})\,,\\
\dot{p}_i^w &= \{p_i^w, H\} = -k\,(1+\varepsilon)\,(u_{i-1}-u_i) - k\,(1-\varepsilon)\,(u_{i+1}-u_i)\,.
\end{aligned} \tag{S.74}$$

The constraint here involves the momenta as well, and read as

$$\begin{aligned}
u_i(t) + w_i(t) &= 0,\\
p_i^u(t) - p_i^w(t) &= 0.
\end{aligned} \tag{S.75}$$

Applying the constraint to the equations of motion yields

$$\ddot{u}_i = -k\,(1+\varepsilon)\,(u_{i-1}-u_i) - k\,(1-\varepsilon)\,(u_{i+1}-u_i)\,. \tag{S.76}$$

The equation of motion of u_i is the same as Eq. (S.72).

E. Feedback-controlled active particles with vision-cone interactions

We next consider light-activated active particles whose motility is controlled by an external feedback loop, consisting of a particle detection algorithm and a scanning laser system [16, 17]. Through this feedback system, particles can be programmed to self-propel only when there are enough other particles in their vision cone [17], thus mimicking the behavior of animals such as midges. To decide whether a given particle i should self-propel or not, a perception metric is computed as

$$P_i = \sum_{j\in V_i^\alpha} \frac{1}{2\pi r_{ij}}, \tag{S.77}$$

where V_i^α denotes the vision cone of angle α around the orientation of particle i (Fig. S1e). Respectively, r_{ij} is the distance between particles i and j, so that the perception metric assigns less weight to more distant particles. If a given particle has a perception metric $P_i > P^*$, it is made to propel at speed v_0, which is achieved by shining light on it through the laser scanning system. Otherwise, the particle remains passive. Thus, particle motion can be modeled as

$$\begin{aligned}
\frac{\mathrm{d}\mathbf{r}_i}{\mathrm{d}t} &= v\,(P_i)\,\hat{\mathbf{n}}_i - \sum_{j\neq i}\frac{1}{\gamma_\mathrm{t}}\frac{\partial U(r_{ij})}{\partial \mathbf{r}_i} + \eta_i^\mathrm{t},\\
\frac{\mathrm{d}\hat{\mathbf{n}}_i}{\mathrm{d}t} &= \hat{\mathbf{n}}_i \times \eta_i^\mathrm{r},
\end{aligned} \tag{S.78}$$

where the self-propulsion speed v is given by

$$v(P_i) = \begin{cases} v_0; & P_i > P^* \\ 0; & P_i < P^*. \end{cases} \tag{S.79}$$

Respectively, γ_t is the translational friction coefficient, η_i^t and η_i^r are the translational and rotational noises, and $U(r_{ij})$ is an interaction potential to model excluded-volume interactions, which can be done through the repulsive Weeks-Chandler-Andersen (WCA) potential given by

$$U(r_{ij}) = \begin{cases} 4\epsilon\left[\left(\frac{\sigma}{r_{ij}}\right)^{12} - \left(\frac{\sigma}{r_{ij}}\right)^{6} + \frac{1}{4}\right], & \text{for } r_{ij} \le \sigma\sqrt[6]{2} \\ 0, & \text{for } r_{ij} > \sigma\sqrt[6]{2}, \end{cases} \tag{S.80}$$

where ϵ is the potential strength and σ is the particle diameter.

We now introduce auxiliary particles with position and orientation denoted by $\mathbf{x}_i$ and $\hat{\mathbf{m}}_i$, respectively, to construct the Hamiltonian embedding

$$H = -\sum_i v\left(P_i\right)\hat{\mathbf{n}}_i \cdot (\mathbf{r}_i + \mathbf{x}_i) + \frac{1}{\gamma_t}\sum_{i<j}\left[U(r_{ij}) - U(x_{ij})\right]. \tag{S.81}$$

We can derive the equations of motion for the particle positions and orientations from the Hamiltonian description as follows

$$\begin{aligned} \frac{\mathrm{d}\mathbf{r}_i}{\mathrm{d}t} &= -\frac{\partial H}{\partial \mathbf{r}_i} = v\left(P_i\right)\hat{\mathbf{n}}_i + \sum_j \frac{\partial v\left(P_j\right)}{\partial \mathbf{r}_i}\hat{\mathbf{n}}_j \cdot \left(\mathbf{r}_j + \mathbf{x}_j\right) - \sum_{j\neq i}\frac{1}{\gamma_t}\frac{\partial U(r_{ij})}{\partial \mathbf{r}_i}, \\ \frac{\mathrm{d}\hat{\mathbf{n}}_i}{\mathrm{d}t} &= \frac{\partial H}{\partial \hat{\mathbf{n}}_i} \times \hat{\mathbf{n}}_i = v\left(P_i\right)(\mathbf{r}_i + \mathbf{x}_i) \times \hat{\mathbf{n}}_i. \end{aligned} \tag{S.82}$$

Finally, the constraints read as

$$\begin{aligned} \mathbf{r}_i(t) + \mathbf{x}_i(t) &= 0, \\ \hat{\mathbf{n}}_i(t) + \hat{\mathbf{m}}_i(t) &= 0. \end{aligned} \tag{S.83}$$

Applying the constraint to the equations of motion yields

$$\begin{aligned} \frac{\mathrm{d}\mathbf{r}_i}{\mathrm{d}t} &= v\left(P_i\right)\hat{\mathbf{n}}_i - \frac{1}{\gamma_t}\sum_{j\neq i}\frac{\partial U(r_{ij})}{\partial \mathbf{r}_i}, \\ \frac{\mathrm{d}\hat{\mathbf{n}}_i}{\mathrm{d}t} &= 0, \end{aligned} \tag{S.84}$$

which is the same as Eq. (S.78), except for the noise terms.

F. Sedimenting particles with hydrodynamic interactions

Sedimenting particles provide a paradigmatic example of a system with velocity-dependent nonreciprocal interactions due to hydrodynamics. When particles settle through a viscous fluid under gravity, they generate flow fields that affect the motion of neighboring particles. In general, the flow generated by particle j impacts particle i differently than the flow generated by particle i impacts particle j (Fig. S1f). Therefore, the resulting interactions are intrinsically nonreciprocal. This nonreciprocity arises not from the particles themselves, but from the asymmetry of the hydrodynamic coupling through the surrounding fluid.

Consider two spherical particles sedimenting under gravity in a Stokes flow regime, where inertial effects are negligible. The equation of motion for particle i reads as

$$\gamma\dot{\mathbf{r}}_i = m_i\mathbf{g} + \sum_{j\neq i}\mathbf{F}_{j\to i}^{\mathrm{hydro}} + \eta_i, \tag{S.85}$$

where m_i is the particle mass, $\mathbf{g}$ is gravitational acceleration, γ is the Stokes drag coefficient (proportional to the fluid viscosity), $\mathbf{F}_{j\to i}^{\mathrm{hydro}}$ denotes the hydrodynamic force exerted on particle i by the flow generated by particle j, and η_i is the noise term.

The hydrodynamic interaction force can be expressed in terms of the Oseen tensor $\mathbf{G}$ (also called the Stokeslet):

$$\mathbf{F}_{j\to i}^{\mathrm{hydro}} = \gamma\mathbf{G}(\mathbf{r}_{ij}) \cdot m_j\mathbf{g}, \tag{S.86}$$

where $\mathbf{r}_{ij} = \mathbf{r}_i - \mathbf{r}_j$ is the inter-particle separation, and the Oseen tensor is given by

$$\mathbf{G}(\mathbf{r}) = \frac{1}{8\pi\eta r}\left(\mathbf{I} + \frac{\mathbf{r}\otimes\mathbf{r}}{r^2}\right), \tag{S.87}$$

with η the dynamic viscosity of the fluid, $\mathbf{I}$ the identity matrix, and $[\mathbf{r}\otimes\mathbf{r}]_{\alpha\beta} = r_\alpha r_\beta$. The symmetry of this tensor implies that $\mathbf{G}(\mathbf{r}_{ij}) = \mathbf{G}(\mathbf{r}_{ji})$, reflecting the directional dependence of hydrodynamic coupling on the separation vector. Therefore, $\mathbf{F}^{\text{hydro}}_{j\to i}$ and $\mathbf{F}^{\text{hydro}}_{i\to j}$ point in the same direction since the masses of particles are all positive. Hence, the interaction forces are not equal and opposite; the interactions are nonreciprocal. Crucially, the nonreciprocal interactions in this system are velocity-*independent*: they depend only on particle positions through the inter-particle distance.

We can now introduce auxiliary particles with position denoted by $\mathbf{x}_i$. The Hamiltonian embedding is given by

$$H = -\sum_i m_i \mathbf{g}\cdot\mathbf{r}_i - \sum_{\langle ij\rangle} \mathbf{F}^{\text{hydro}}_{j\to i}\cdot(\mathbf{x}_i + \mathbf{r}_i). \tag{S.88}$$

The corresponding equation of motion from this Hamiltonian description reads as

$$\frac{\mathrm{d}\mathbf{r}_i}{\mathrm{d}t} = -\frac{\partial H}{\partial \mathbf{r}_i} = m_i\mathbf{g} + \sum_{j\in\langle ij\rangle} \mathbf{F}^{\text{hydro}}_{j\to i} + \sum_{j\in\langle ij\rangle} \frac{\partial}{\partial \mathbf{r}_i}\mathbf{F}^{\text{hydro}}_{j\to i}\cdot(\mathbf{x}_i + \mathbf{r}_i). \tag{S.89}$$

Moreover, the constraint is

$$\mathbf{x}_i(t) + \mathbf{r}_i(t) = 0. \tag{S.90}$$

Applying the constraints to the equations of motion yields

$$\frac{\mathrm{d}\mathbf{r}_i}{\mathrm{d}t} = m_i\mathbf{g} + \sum_{j\in\langle ij\rangle} \mathbf{F}^{\text{hydro}}_{j\to i}, \tag{S.91}$$

which is equivalent to Eq. (S.85), up to the noise term.

S3. SYMPLECTIC STRUCTURE OF THE HAMILTONIAN EMBEDDING: IMPLEMENTING THE CONSTRAINT FOR INTERACTIONS WITH OVERDAMPED AND UNDAMPED NONRECIPROCAL DYNAMICS

In Sec. S1, we introduced Hamiltonian embeddings and outlined the appropriate constraints for several nonreciprocal systems, both for damped and undamped dynamics in Sec. S2. Here, we elaborate on these two cases separately, discussing in detail the implementation of the constraint and the corresponding symplectic structure. Although the discussion below is general, for concreteness we shall illustrate the procedure using the vision-cone XY spins as an example, for which the interaction $J_{ij}(\theta_i)$ takes the form

$$J_{ij}(\theta_i) = \begin{cases} J, & \text{for } \min\left\{2\pi - \left|\theta_i - \psi_{ij}\right|, \left|\theta_i - \psi_{ij}\right|\right\} \le \Psi \\ 0, & \text{else.} \end{cases} \tag{S.92}$$

Below, we start by introducing the form of the Lagrangian and then derive the corresponding Hamiltonian, from which the dissipative and inertial nonreciprocal equations of motion can be obtained, along with the associated Poisson bracket relations. Subsequently, we impose the appropriate constraint and demonstrate that it is preserved at all times.

A. Overdamped nonreciprocal systems

Consider a dissipative system with nonreciprocal interactions in the equations of motion (S.55). Since the equation of motion is first-order in θ, we consider a kinetic energy term linear in $\dot\theta$, expressed as $\mathcal{K} = \sum_i \varphi_i\dot\theta_i$. Such kinetic energy terms are commonly used to describe charged particles on a plane exposed to a perpendicular magnetic field [18]. In addition, we denote by $\mathcal{U}$ the symmetrized interactions, cf. Eq. (S.56). The explicit form of the Lagrangian is given by

$$\begin{aligned}\mathcal{L} &= \mathcal{K} - \mathcal{U} \\ &= \sum_i \varphi_i\dot\theta_i + \sum_{\langle ij\rangle}\left\{J_{ij}(\theta_i)\left[\cos(\theta_i - \theta_j) + \cos(\varphi_i - \theta_j)\right] + J_{ji}(\theta_j)\left[\cos(\theta_j - \theta_i) + \cos(\varphi_j - \theta_i)\right]\right\}.\end{aligned} \tag{S.93}$$

From this Lagrangian, we can derive the canonical momentum associated with θ_i as

$$p_i = \frac{\partial \mathcal{L}}{\partial \dot{\theta}_i} = \varphi_i, \tag{S.94}$$

which leads to the Poisson bracket relation $\{\theta_i, \varphi_j\} = \delta_{ij}$. This implies that the original spins θ_i and the auxiliary spins φ_i are conjugate degrees of freedom. Therefore, they define a phase space that is a torus (i.e., compact).

Using the definition for the canonical momenta, we can construct the corresponding Hamiltonian from the Lagrangian $\mathcal{L}$ by performing a Legendre transform

$$\begin{aligned}\mathcal{H} &= \sum_i p_i \dot{\theta}_i - \mathcal{L} = \mathcal{U} \\ &= -\sum_{\langle ij \rangle} \left\{ J_{ij}(\theta_i) \left[\cos(\theta_i - \theta_j) + \cos(\varphi_i - \theta_j) \right] + J_{ji}(\theta_j) \left[\cos(\theta_j - \theta_i) + \cos(\varphi_j - \theta_i) \right] \right\}. \end{aligned} \tag{S.95}$$

Thus, we recover the Hamiltonian introduced in the Main Text, cf. Eq. (**??**). Based on the above result, we can derive the dynamics of θ_i and φ_i

$$\begin{aligned}\dot{\theta}_i &= \{\theta_i, \mathcal{H}\} = \sum_{j \in \langle ij \rangle} J_{ij}(\theta_i) \sin(\varphi_i - \theta_j), \\ \dot{\varphi}_i &= \{\varphi_i, \mathcal{H}\} = \sum_{j \in \langle ij \rangle} \Big\{ -J_{ij}(\theta_i) \sin(\theta_i - \theta_j) \\ &\qquad + \partial_{\theta_i} J_{ij}(\theta_i) \left[\cos(\theta_i - \theta_j) + \cos(\varphi_i - \theta_j) \right] - J_{ji}(\theta_j) \left[\sin(\theta_i - \theta_j) + \sin(\theta_i - \varphi_j) \right] \Big\}. \end{aligned} \tag{S.96}$$

Now, let us define the constraint G_i in phase space as the difference between the degrees of freedom θ_i and φ_i,

$$G_i = \theta_i - \varphi_i. \tag{S.97}$$

Using Eq. (S.96), the equation of motion of the constraint is given by

$$\begin{aligned}\frac{\mathrm{d}G_i}{\mathrm{d}t} &= \{G_i, \mathcal{H}\} = \sum_j \partial_{\theta_j} G_i \partial_{\varphi_j} \mathcal{H} - \partial_{\varphi_j} G_i \partial_{\theta_j} \mathcal{H} = \dot{\theta}_i - \dot{\varphi}_i \\ &= \sum_{j \in \langle ij \rangle} J_{ij}(\theta_i) \sin(\varphi_i - \theta_j) - \sum_{j \in \langle ij \rangle} \Big\{ J_{ij}(\theta_i) \sin(\theta_i - \theta_j) \\ &\qquad + \partial_{\theta_i} J_{ij}(\theta_i) \left[\cos(\theta_i - \theta_j) + \cos(\varphi_i - \theta_j) \right] - J_{ji}(\theta_j) \left[\sin(\theta_i - \theta_j) + \sin(\theta_i - \varphi_j) \right] \Big\}. \end{aligned} \tag{S.98}$$

When the degrees of freedom lie on the constraint surface, i.e., $G_i = \pi$, the following relations hold

$$\begin{aligned}\cos(\theta_i - \theta_j) + \cos(\varphi_i - \theta_j) &= 0, \\ \sin(\theta_i - \theta_j) + \sin(\theta_i - \varphi_j) &= 0. \end{aligned} \tag{S.99}$$

This results in the value of Eq. (S.98) being zero. Therefore, the constraint is preserved by the dynamics. Consequently, similar to the discussion in Eq. (S.8), if initially the system is under the constraint, $G_i(t = 0) = \pi$, the constraint is preserved at all times: $G_i(t) = \pi$.

Under the constraint, the equations of motion are given by

$$\begin{aligned}\dot{\theta}_i &= \sum_{j \in \langle ij \rangle} J_{ij}(\theta_i) \sin(\varphi_i - \theta_j) = -\sum_{j \in \langle ij \rangle} J_{ij}(\theta_i) \sin(\theta_i - \theta_j), \\ \dot{\varphi}_i &= \sum_{j \in \langle ij \rangle} -J_{ij}(\theta_i) \sin(\theta_i - \theta_j) = -\sum_{j \in \langle ij \rangle} J_{ij}(\theta_i) \sin(\varphi_i - \varphi_j). \end{aligned} \tag{S.100}$$

Thus, the application of the constraint to the Hamiltonian embedding allows us to retrieve the overdamped nonreciprocal dynamics for the original degrees of freedom θ_i.

B. Undamped nonreciprocal systems

Inspired by the approach in the previous subsection, we introduce a general procedure to construct the Hamiltonian embedding for undamped systems with nonreciprocal interactions. We will provide the explicit form of the Hamiltonian for the vision-cone XY spins, and explain how the dynamics naturally emerge from the constraint.

We first introduce the Lagrangian. It is composed of two terms: the first one is the quadratic kinetic energy $\mathcal{K} = \sum_i(\frac{1}{2}\dot{\theta}_i^2 - \frac{1}{2}\dot{\varphi}_i^2)$ and the second one is the interaction energy $\mathcal{U}$ (see below for an explanation of the negative kinetic energy for φ). The explicit form of the Lagrangian for undamped nonreciprocal systems is given by

$$\begin{aligned}\mathcal{L} &= \mathcal{K} - \mathcal{U}\\ &= \sum_i\left(\frac{1}{2}\dot{\theta}_i^2 - \frac{1}{2}\dot{\varphi}_i^2\right) + \sum_{\langle ij\rangle}\left\{J_{ij}\left(\theta_i\right)\left[\cos\!\left(\theta_i - \theta_j\right) + \cos\!\left(\varphi_i - \theta_j\right)\right] + J_{ji}\left(\theta_j\right)\left[\cos\!\left(\theta_j - \theta_i\right) + \cos\!\left(\varphi_j - \theta_i\right)\right]\right\}. \end{aligned} \tag{S.101}$$

From this Lagrangian, we can derive the conjugate degree of freedom (i.e., the canonical momenta) associated with θ_i and φ_i:

$$L_i^\theta = \frac{\partial\mathcal{L}}{\partial\dot{\theta}_i} = \dot{\theta}_i, \qquad L_i^\varphi = \frac{\partial\mathcal{L}}{\partial\dot{\varphi}_i} = -\dot{\varphi}_i, \tag{S.102}$$

which leads to the Poisson bracket relations $\{\theta_i, L_i^\theta\} = \delta_{ij}$ and $\{\varphi_i, L_i^\varphi\} = \delta_{ij}$. Unlike the overdamped case, here we end up with two spins; for each, the phase space is a semi-compact cylinder, since the original coordinate angle is compact but angular momentum is not.

Using the canonical momenta, we can construct the corresponding Hamiltonian $\mathcal{H}$ from the Lagrangian $\mathcal{L}$ using a Legendre transform:

$$\begin{aligned}\mathcal{H} &= \sum_i\left(L_i^\theta\dot{\theta}_i + L_i^\varphi\dot{\varphi}_i\right) - \mathcal{L} = \mathcal{K} + \mathcal{U} \qquad \text{(S.103)}\\ &= \frac{1}{2}\sum_i\left[\left(L_i^\theta\right)^2 - (L_i^\varphi)^2\right] - \sum_{\langle ij\rangle}\left\{J_{ij}\left(\theta_i\right)\left[\cos\!\left(\theta_i - \theta_j\right) + \cos\!\left(\varphi_i - \theta_j\right)\right] + J_{ji}\left(\theta_j\right)\left[\cos\!\left(\theta_j - \theta_i\right) + \cos\!\left(\varphi_j - \theta_i\right)\right]\right\}.\end{aligned}$$

An unusual feature of the kinetic energy $\mathcal{K}$ is that it is negative-definite for the auxiliary degree of freedom φ_j. As elaborated on above, mathematically this is required to maintain the constraint $\theta_i(t) = \varphi_i(t) + \pi$ at all times.

Based on the above result, we derive the dynamics of θ_i, φ_i, L_i^θ, and L_i^φ as follows:

$$\begin{aligned}\dot{\theta}_i &= \{\theta_i, \mathcal{H}\} = L_i^\theta,\\ \dot{\varphi}_i &= \{\varphi_i, \mathcal{H}\} = -L_i^\varphi,\\ \dot{L}_i^\theta &= \left\{L_i^\theta, \mathcal{H}\right\} = -\sum_{j\in\langle ij\rangle}\Big\{J_{ij}(\theta_i)\sin\!\left(\theta_i - \theta_j\right)\\ &\qquad +\partial_{\theta_i}J_{ij}(\theta_i)\left[\cos\!\left(\theta_i - \varphi_j\right) + \cos\!\left(\theta_i - \theta_j\right)\right] + J_{ji}(\theta_j)\left[\sin\!\left(\theta_i - \theta_j\right) + \sin\!\left(\theta_i - \varphi_j\right)\right]\Big\},\\ \dot{L}_i^\varphi &= \left\{L_i^\varphi, \mathcal{H}\right\} = -\sum_{j\in\langle ij\rangle}J_{ij}(\theta_i)\sin\!\left(\varphi_i - \theta_j\right).\end{aligned} \tag{S.104}$$

Now let us introduce two sets of constraints $G_i^{(1)}$ and $G_i^{(2)}$. The first set represents the difference between the degrees of freedom θ_i and φ_i,

$$G_i^{(1)} = \theta_i - \varphi_i, \tag{S.105}$$

and the second concerns the sum of angular momenta

$$G_i^{(2)} = L_i^\theta + L_i^\varphi. \tag{S.106}$$

Using Eq. (S.104), the equations of motion of the constraints, $G_i^{(1)}$ and $G_i^{(2)}$, are given by

$$\frac{\mathrm{d}G_i^{(1)}}{\mathrm{d}t} = \left\{G_i^{(1)}, \mathcal{H}\right\} = L_i^\theta + L_i^\varphi = G_i^{(2)} \tag{S.107}$$

$$\begin{aligned}\frac{\mathrm{d}G_i^{(2)}}{\mathrm{d}t} &= \left\{G_i^{(2)}, \mathcal{H}\right\} = \dot{L}_i^\theta + \dot{L}_i^\varphi\\ &= -\sum_{j\in\langle ij\rangle}J_{ij}(\theta_i)\sin\!\left(\theta_i - \theta_j\right) - \sum_{j\in\langle ij\rangle}\Big\{J_{ij}(\theta_i)\sin\!\left(\varphi_i - \theta_j\right)\\ &\qquad +\partial_{\theta_i}J_{ij}(\theta_i)\left[\cos\!\left(\theta_i - \varphi_j\right) + \cos\!\left(\theta_i - \theta_j\right)\right] + J_{ji}(\theta_j)\left[\sin\!\left(\theta_i - \theta_j\right) + \sin\!\left(\theta_i - \varphi_j\right)\right]\Big\}.\end{aligned} \tag{S.108}$$

When the system is on the constraint surface in phase space, defined by $G_i^{(1)} = \pi$ and $G_i^{(2)} = 0$, the time derivatives of the constraints are all zero. In particular, the constraint $G_i^{(2)} = 0$, which represents the opposite direction of the angular momenta, will make Eq. (S.107) evaluate to zero:

$$\frac{\mathrm{d}G_i^{(1)}}{\mathrm{d}t} = 0. \tag{S.109}$$

Simultaneously, the constraint $G_i^{(1)}(0) = \pi$, which represents the opposite direction of the spins, leads to the following condition:

$$\begin{aligned} \cos\left(\theta_i - \theta_j\right) + \cos\left(\varphi_i - \theta_j\right) = 0, \\ \sin\left(\theta_i - \theta_j\right) + \sin\left(\varphi_i - \theta_j\right) = 0. \end{aligned} \tag{S.110}$$

This results in the value of Eq. (S.108) being zero:

$$\frac{\mathrm{d}G_i^{(2)}}{\mathrm{d}t} = 0. \tag{S.111}$$

Similar to the overdamped case above, these constraints hold at all times.

Under the constraints, the equations of motion are given by

$$\begin{aligned} \dot{\theta}_i &= \{\theta_i, \mathcal{H}\} = L_i^\theta, \\ \dot{\varphi}_i &= \{\varphi_i, \mathcal{H}\} = -L_i^\varphi, \\ \dot{L}_i^\theta &= \left\{L_i^\theta, \mathcal{H}\right\} = -\sum_{j\in\langle ij\rangle} J_{ij}(\theta_i)\sin\bigl(\theta_i - \theta_j\bigr) \\ \dot{L}_i^\varphi &= \left\{L_i^\varphi, \mathcal{H}\right\} = \sum_{j\in\langle ij\rangle} J_{ij}(\theta_i)\sin\bigl(\varphi_i - \varphi_j\bigr). \end{aligned} \tag{S.112}$$

We can also write them in the form of the second-order time derivative of θ_i and φ_i,

$$\begin{aligned} \ddot{\theta}_i &= -\sum_{j\in\langle ij\rangle} J_{ij}(\theta_i)\sin\bigl(\theta_i - \theta_j\bigr), \\ \ddot{\varphi}_i &= -\sum_{j\in\langle ij\rangle} J_{ij}(\theta_i)\sin\bigl(\varphi_i - \varphi_j\bigr). \end{aligned} \tag{S.113}$$

Thus, the application of the constraints to the Hamiltonian embedding allows us to retrieve the undamped nonreciprocal dynamics for the original degree of freedom θ_i.

The occurrence of negative kinetic energy for the auxiliary degrees of freedom can be explained as follows: Consider first the free system where the degrees of freedom are noninteracting, $\mathcal{U} = 0$, and both terms in the kinetic energy are positive; then, by conservation of angular momentum $L_j^\varphi + L_j^\theta = 0$, the θ and φ degrees of freedom will rotate opposite to one another, thus violating the constraint that pins them to move together (i.e., in the same direction). To enforce the latter without over-constraining the dynamics of the resulting joint motion, a negative kinetic energy is required. Returning to the Hamiltonian (S.103), more formally, as discussed above, we need to construct a constraint as $\theta_i(t) = \varphi_i(t) + \pi$. Taking two time derivatives, we have $\ddot{\theta}_i(t) = \ddot{\varphi}_i(t)$. If the interactions between the original and the auxiliary degrees of freedom are defined as in Eq. (S.56), the torques experienced by the original spin and the auxiliary spin will be oppositely directed under the imposed constraint, as given by $-\sum_{j\in\langle ij\rangle} J_{ij}\sin\bigl(\theta_i - \theta_j\bigr) = -\bigl[-\sum_{j\in\langle ij\rangle} J_{ij}\sin\bigl(\varphi_i - \theta_j\bigr)\bigr]$. To maintain the opposite sign in the EOM of the angular momenta, the kinetic energy for the auxiliary degree of freedom has a negative sign, $-\frac{1}{2}\dot{\varphi}_i^2$. Such negative kinetic energy terms for the auxiliary degree of freedom have been discussed in Ref. [19] where they arise as a result of reformulating the action principle as an initial- (rather than a boundary-) value problem. Similarly, in the programmed robotic metamaterials example from Sec. S2 D, we used the kinetic term $\frac{1}{2}\sum_i \left(p_i^u\right)^2 - \left(p_i^w\right)^2$ to construct the Hamiltonian. Such anisotropic kinetic energy terms have also been encountered before in the description of azimuthal curl forces [20].

As we showed at the beginning of Appendix S1, one can extend the arguments of this section to more general nonreciprocal systems whose equations of motion feature both inertial and dissipative terms.

S4. EQUIVALENCE BETWEEN NONRECIPROCAL LANGEVIN DYNAMICS AND GLAUBER MONTE-CARLO DYNAMICS

This section demonstrates that the steady-state and non-stationary density distributions resulting from the original nonreciprocal Langevin dynamics and from the constrained Glauber dynamics, based on the Hamiltonian that we propose, are identical. In

Sec. S4 A, we first derive a Fokker-Planck-like equation for the phase-space density distribution obtained from Langevin dynamics; then, in Sec. S4 B, we prove that it is equivalent to the corresponding distribution obtained using constrained Glauber dynamics.

Below, we consider for concreteness the nonreciprocal Hamiltonian for the vision-cone interactions (see Main Text for the details)

$$H = H_{SS} + H_{Sa} = -\sum_{\langle ij\rangle}\left\{\left[J_{ij}(\theta_i) + J_{ji}(\theta_j)\right]\cos\!\left(\theta_i - \theta_j\right) + J_{ij}(\theta_i)\cos\!\left(\theta_j - \varphi_i\right) + J_{ji}(\theta_j)\cos\!\left(\theta_i - \varphi_j\right)\right\}, \tag{S.114}$$

although the procedure is general and applies to other models (see Sec. S2).

Before presenting the detailed derivations, we briefly clarify the notions of detailed balance and global balance [21] for Markov dynamics. Let C denote a microstate (configuration), e.g., the set of spin angles $\{\theta_i, \varphi_i\}$ of the Hamiltonian embedding in Eq. (S.114), and let $W(C \to C')$ be the transition rate of the Markov dynamics from C to C'.

Detailed balance requires the probability current to vanish *pairwise*:

$$\rho(C)\, W(C \to C') = \rho(C')\, W(C' \to C) \qquad \forall\, C, C'. \tag{S.115}$$

This condition implies the absence of steady-state probability currents and is characteristic of equilibrium dynamics.

Global balance is weaker: it only requires that, for every microstate, the *total* incoming probability flux equals the *total* outgoing flux,

$$\sum_{C'} \rho(C')\, W(C' \to C) = \rho(C) \sum_{C'} W(C \to C'), \qquad \forall\, C, \tag{S.116}$$

thereby ensuring stationarity while allowing for nonzero circulating currents. Since nonreciprocal interactions generically drive the system out of equilibrium, detailed balance is not expected to hold; accordingly, in the following, we take global balance to be the appropriate steady-state condition. We will state the global-balance condition in its most general form and characterize the class of stationary distributions consistent with it in our setting. At the same time, in specific models a stronger condition (intermediate between global and detailed balance) may hold; for instance, in Eq. (S.116) the sum over C' may effectively be restricted to the subset of configurations that are dynamically connected to C. Also, note that detailed balance implies global balance, but not vice versa.

Finally, if global balance in Eq. (S.116) is violated, the density distribution is *non-stationary*. In that case, the time evolution of the density is governed by the master equation

$$\partial_t \rho(C) = \sum_{C'} \rho(C')\, W(C' \to C) - \rho(C) \sum_{C'} W(C \to C'), \qquad \forall\, C. \tag{S.117}$$

Although we do not use this relation directly in our proof, it can be recovered naturally from the derivations below.

A. Langevin steady-state distribution

Recall that imposing the constraint $\theta_i(t) - \varphi_i(t) = \pi$, and adding the same noise term for both the original and the auxiliary degrees of freedom results in the overdamped Langevin equations

$$\begin{aligned}\dot{\theta}_i &= \{\theta_i, H\} + \sqrt{2T}\eta_i(t) = \partial_{\varphi_i} H|_{\text{constr.}} + \sqrt{2T}\eta_i(t),\\ \dot{\varphi}_i &= \{\varphi_i, H\} + \sqrt{2T}\eta_i(t) = -\partial_{\theta_i} H|_{\text{constr.}} + \sqrt{2T}\eta_i(t),\end{aligned} \tag{S.118}$$

with conjugate degrees of freedom $\{\theta_i, \varphi_j\}$=δ_{ij}. To maintain consistency with the traditional form of Langevin dynamics and represent the equations of motion on the constraint surface, the notation $\partial_{\theta_i} H|_{\text{constr.}}$ is introduced to replace the Poisson bracket. Here $\eta_i(t)$ denotes white noise with $\langle \eta_i(t)\rangle = 0$ and $\langle \eta_i(t)\eta_j(t')\rangle = \delta_{ij}\delta(t - t')$, with T the temperature of the bath, where the average is taken over the phase-space density $\rho(\vec{\theta}(t), \vec{\varphi}(t))$. Note that the constraint implies that each pair (θ, φ_i) experiences the same noise realization.

Below, we first consider steady-state distributions (i.e., no explicit t-dependence in ρ, Sec. S4 A 1), and then generalize the result to non-stationary distributions in Sec. S4 A 2.

1. *Steady-state distribution*

To obtain the steady-state phase-space density distribution $\rho(\vec{\theta}(t), \vec{\varphi}(t))$ under the above dynamics, we follow the standard procedure [22, 23] and introduce a test function $f(\vec{\theta}(t), \vec{\varphi}(t))$ over phase space. The change of the test function within a small

time window Δt is to leading order

$$\begin{aligned}
& f\left(\vec{\theta}(t+\Delta t), \vec{\varphi}(t+\Delta t)\right) - f\left(\vec{\theta}(t), \vec{\varphi}(t)\right) \\
&= \sum_i \frac{\partial f}{\partial \theta_i}\dot{\theta}_i \Delta t + \frac{\partial f}{\partial \varphi_i}\dot{\varphi}_i \Delta t + \frac{\partial^2 f}{2\partial\theta_i\partial\theta_i}(\dot{\theta}_i\Delta t)^2 + \frac{\partial^2 f}{2\partial\varphi_i\partial\varphi_i}(\dot{\varphi}_i\Delta t)^2 + \frac{\partial^2 f}{\partial\varphi_i\partial\theta_i}\dot{\varphi}_i\dot{\theta}_i(\Delta t)^2 \\
&+ \sum_{i\neq j} \frac{\partial^2 f}{\partial\theta_i\partial\theta_j}\dot{\theta}_i\dot{\theta}_j(\Delta t)^2 + \frac{\partial^2 f}{\partial\varphi_i\partial\varphi_j}\dot{\varphi}_i\dot{\varphi}_j(\Delta t)^2 + \cdots \, .
\end{aligned} \tag{S.119}$$

For steady-state distributions, at long times the expectation value of the test function should be time-independent:

$$\langle f\,(t+\Delta t)\rangle = \langle f\,(t)\rangle, \tag{S.120}$$

which can be written infinitesimally as

$$\int \frac{\mathrm{d}f(\vec{\theta},\vec{\varphi})}{\mathrm{d}t}\rho(\vec{\theta},\vec{\varphi})\prod_j \mathrm{d}\theta_j \mathrm{d}\varphi_j = \lim_{\Delta t\to 0}\int \frac{f\left(\vec{\theta}(t+\Delta t), \vec{\varphi}(t+\Delta t)\right) - f\left(\vec{\theta}(t), \vec{\varphi}(t)\right)}{\Delta t}\rho(\vec{\theta},\vec{\varphi})\prod_j \mathrm{d}\theta_j \mathrm{d}\varphi_j = 0. \tag{S.121}$$

To proceed with the derivation, we write the noise terms in Eq. (S.118) as a Wiener process $W_i(t)$:

$$\begin{aligned}
\dot{\theta}_i \Delta t &= \partial_{\varphi_i} H|_{\text{constr.}}\Delta t + \sqrt{2T}\Delta W_i(t), \\
\dot{\varphi}_i \Delta t &= -\partial_{\theta_i} H|_{\text{constr.}}\Delta t + \sqrt{2T}\Delta W_i(t),
\end{aligned} \tag{S.122}$$

where $\langle \Delta W_i(t)\rangle = 0$ and $\langle \Delta W_i(t)\Delta W_j(t')\rangle = \Delta t\delta(t-t')\delta_{ij}$.

In the following, we substitute Eq. (S.119) in Eq. (S.121). Focusing on the θ_i terms, the first-order contribution can be evaluated as

$$\begin{aligned}
\int \frac{\partial f}{\partial\theta_i}\dot{\theta}_i\Delta t\; \rho\left(\vec{\theta},\vec{\varphi}\right)\mathrm{d}\theta_i\mathrm{d}\varphi_i &= \int \frac{\partial f}{\partial\theta_i}\left[\left.\frac{\partial H}{\partial\varphi_i}\right|_{\text{constr.}}\Delta t + \sqrt{2T}\Delta W_i(t)\right]\rho\left(\vec{\theta},\vec{\varphi}\right)\mathrm{d}\theta_i\mathrm{d}\varphi_i \\
&= \Delta t\int \frac{\partial f}{\partial\theta_i}\left.\frac{\partial H}{\partial\varphi_i}\right|_{\text{constr.}}\rho\left(\vec{\theta},\vec{\varphi}\right)\mathrm{d}\theta_i\mathrm{d}\varphi_i,
\end{aligned} \tag{S.123}$$

where we used the equation of motion and the property $\langle\Delta W_i(t)\rangle = 0$ in the last line. Similarly, the second order in Δt gives

$$\begin{aligned}
&\int \frac{\partial^2 f}{\partial\theta_i\partial\theta_j}\dot{\theta}_i\dot{\theta}_j\Delta t^2\rho\left(\vec{\theta},\vec{\varphi}\right)\mathrm{d}\theta_i\mathrm{d}\varphi_i = \\
&= \int \frac{\partial^2 f}{\partial\theta_i\partial\theta_j}\left[\left.\frac{\partial H}{\partial\varphi_i}\right|_{\text{constr.}}\Delta t + \sqrt{2T}\Delta W_i(t)\right]\left[\left.\frac{\partial H}{\partial\varphi_j}\right|_{\text{constr.}}\Delta t + \sqrt{2T}\Delta W_j(t)\right]\rho\left(\vec{\theta},\vec{\varphi}\right)\mathrm{d}\theta_i\mathrm{d}\varphi_i \\
&= \int 2T\frac{\partial^2 f}{\partial\theta_i\partial\theta_j}\Delta W_i(t)\Delta W_j(t)\rho\left(\vec{\theta},\vec{\varphi}\right)\mathrm{d}\theta_i\mathrm{d}\varphi_i + \sqrt{2T}\int \frac{\partial^2 f}{\partial\theta_i\partial\theta_j}\left.\frac{\partial H}{\partial\varphi_j}\right|_{\text{constr.}}\Delta W_i(t)\rho\left(\vec{\theta},\vec{\varphi}\right)\Delta t\mathrm{d}\theta_i\mathrm{d}\varphi_i \\
&+\sqrt{2T}\int \frac{\partial^2 f}{\partial\theta_i\partial\theta_j}\left.\frac{\partial H}{\partial\varphi_i}\right|_{\text{constr.}}\Delta W_j(t)\rho\left(\vec{\theta},\vec{\varphi}\right)\Delta t\mathrm{d}\theta_i\mathrm{d}\varphi_i + \Delta t^2\int \frac{\partial^2 f}{\partial\theta_i\partial\theta_j}\left(\left.\frac{\partial H}{\partial\varphi_i}\right|_{\text{constr.}}\left.\frac{\partial H}{\partial\varphi_j}\right|_{\text{constr.}}\right)\rho\left(\vec{\theta},\vec{\varphi}\right)\mathrm{d}\theta_i\mathrm{d}\varphi_i \\
&= \Delta t\delta_{ij}2T\int \frac{\partial^2 f}{\partial\theta_i\partial\theta_j}\rho\left(\vec{\theta},\vec{\varphi}\right)\mathrm{d}\theta_i\mathrm{d}\varphi_i + O\left[(\Delta t)^2\right],
\end{aligned} \tag{S.124}$$

where we neglect terms of order Δt^2 since they vanish in the infinitesimal limit $\Delta t \to 0$. The expressions for the other terms in Eq. (S.119) can be derived using the same reasoning.

Collecting all Δt contributions, and plugging them back to Eq. (S.121) leads to the following condition

$$\begin{aligned}
\sum_i \int \prod_{j\neq i}\mathrm{d}\theta_j\mathrm{d}\varphi_j \int \mathrm{d}\theta_i\mathrm{d}\varphi_i\Bigg[&\frac{\partial f}{\partial\theta_i}\left(\left.\frac{\partial H}{\partial\varphi_i}\right|_{\text{constr.}}\right) - \frac{\partial f}{\partial\varphi_i}\left(\left.\frac{\partial H}{\partial\theta_i}\right|_{\text{constr.}}\right) \\
&+T\left(\frac{\partial^2 f}{\partial\theta_i\partial\theta_i} + \frac{\partial^2 f}{\partial\varphi_i\partial\varphi_i} + 2\frac{\partial^2 f}{\partial\varphi_i\partial\theta_i}\right)\Bigg]\rho\left(\vec{\theta},\vec{\varphi}\right) = 0\,.
\end{aligned} \tag{S.125}$$

Noticing that the term proportional to T is a perfect square, we have

$$\sum_i \int \prod_{j\neq i} \mathrm{d}\theta_j \mathrm{d}\varphi_j \int \mathrm{d}\theta_i \mathrm{d}\varphi_i \left[\frac{\partial f}{\partial \theta_i} \left(\left.\frac{\partial H}{\partial \varphi_i}\right|_{\text{constr.}} \right) - \frac{\partial f}{\partial \varphi_i} \left(\left.\frac{\partial H}{\partial \theta_i}\right|_{\text{constr.}} \right) + T \left(\frac{\partial}{\partial \theta_i} + \frac{\partial}{\partial \varphi_i} \right)^2 f \right] \rho\left(\vec{\theta}, \vec{\varphi}\right) = 0. \tag{S.126}$$

Under the constraint $\theta_i - \varphi_i = \pi$, the dynamics of the φ_i spins mirrors that of the θ_i spins. Hence, we have $\partial_{\varphi_i} H|_{\text{constr.}} = -\partial_{\theta_i} H|_{\text{constr.}}$. Plugging this into Eq. (S.126) leads to

$$\sum_i \int \prod_{j\neq i} \mathrm{d}\theta_j \mathrm{d}\varphi_j \int \mathrm{d}\theta_i \mathrm{d}\varphi_i \left[-\left(\frac{\partial}{\partial \theta_i} + \frac{\partial}{\partial \varphi_i} \right) f \left.\frac{\partial H}{\partial \theta_i}\right|_{\text{constr.}} + T \left(\frac{\partial}{\partial \theta_i} + \frac{\partial}{\partial \varphi_i} \right)^2 f \right] \rho\left(\vec{\theta}, \vec{\varphi}\right) = 0. \tag{S.127}$$

If we integrate the T term by parts and, recalling there is no boundary term for a compact phase space, we obtain

$$\sum_i \int \prod_{j\neq i} \mathrm{d}\theta_j \mathrm{d}\varphi_j \int \mathrm{d}\theta_i \mathrm{d}\varphi_i f \left[\left(\frac{\partial}{\partial \theta_i} + \frac{\partial}{\partial \varphi_i} \right) \left(\left.\frac{\partial H}{\partial \theta_i}\right|_{\text{constr.}} \rho\left(\vec{\theta}, \vec{\varphi}\right) \right) + T \left(\frac{\partial}{\partial \theta_i} + \frac{\partial}{\partial \varphi_i} \right)^2 \rho\left(\vec{\theta}, \vec{\varphi}\right) \right] = 0. \tag{S.128}$$

Since this equation for the steady state is valid for all test functions f, we find that the phase-space density in the steady state satisfies

$$\sum_i \left(\frac{\partial}{\partial \theta_i} + \frac{\partial}{\partial \varphi_i} \right) \left[\left.\frac{\partial H}{\partial \theta_i}\right|_{\text{constr.}} \rho\left(\vec{\theta}, \vec{\varphi}\right) + T \left(\frac{\partial}{\partial \theta_i} + \frac{\partial}{\partial \varphi_i} \right) \rho\left(\vec{\theta}, \vec{\varphi}\right) \right] = 0. \tag{S.129}$$

So far the derivation was generic since we did not make use of the precise form of the equations of motion. We will show in Sec. S4 B that Eq. (S.129) governs also the constrained Glauber dynamics.

2. *Time-dependent non-stationary distribution*

Before we do that, let us complete the analysis and examine the case where the density distribution exhibits explicit time dependence, $\rho(\vec{\theta}(t), \vec{\varphi}(t), t)$. Such situations can occur away from equilibrium where oscillating states arise.

First, we allow the test function to include explicit time dependence. Building on the same analytical framework as above, the evolution of the expectation value of the test function takes the form

$$\frac{\mathrm{d}\langle f \rangle}{\mathrm{d}t} = \left\langle \frac{\partial f}{\partial t} \right\rangle + \sum_i \left(\left\langle \frac{\partial f}{\partial \theta_i} \left.\frac{\partial H}{\partial \varphi_i}\right|_{\text{constr.}} \right\rangle - \left\langle \frac{\partial f}{\partial \varphi_i} \left.\frac{\partial H}{\partial \theta_i}\right|_{\text{constr.}} \right\rangle + T \left\langle \left(\frac{\partial}{\partial \theta_i} + \frac{\partial}{\partial \varphi_i} \right)^2 f \right\rangle \right), \tag{S.130}$$

which can be written as

$$\frac{\mathrm{d}}{\mathrm{d}t} \int \prod_j \mathrm{d}\theta_j \mathrm{d}\varphi_j f \rho = \tag{S.131}$$

$$= \int \prod_j \mathrm{d}\theta_j \mathrm{d}\varphi_j \frac{\partial f}{\partial t} \rho + \sum_i \int \prod_{j\neq i} \mathrm{d}\theta_j \mathrm{d}\varphi_j \int \mathrm{d}\theta_i \mathrm{d}\varphi_i \left[\frac{\partial f}{\partial \theta_i} \left.\frac{\partial H}{\partial \varphi_i}\right|_{\text{constr.}} \rho - \frac{\partial f}{\partial \varphi_i} \left.\frac{\partial H}{\partial \theta_i}\right|_{\text{constr.}} \rho + T \int \left(\frac{\partial}{\partial \theta_i} + \frac{\partial}{\partial \varphi_i} \right)^2 f \rho \right].$$

On the other hand, directly from the left-hand side of the equation above, we have

$$\frac{\mathrm{d}}{\mathrm{d}t} \int \prod_j \mathrm{d}\theta_j \mathrm{d}\varphi_j f \rho = \int \prod_j \mathrm{d}\theta_j \mathrm{d}\varphi_j \left(\frac{\partial f}{\partial t} \rho + f \frac{\partial \rho}{\partial t} \right). \tag{S.132}$$

Setting Eq.(S.131) equal to Eq.(S.132) yields

$$\int \prod_j \mathrm{d}\theta_j \mathrm{d}\varphi_j f \frac{\partial \rho}{\partial t} = \sum_i \int \prod_{j\neq i} \mathrm{d}\theta_j \mathrm{d}\varphi_j \int \mathrm{d}\theta_i \mathrm{d}\varphi_i \left[\frac{\partial f}{\partial t} \rho + \frac{\partial f}{\partial \theta_i} \left.\frac{\partial H}{\partial \varphi_i}\right|_{\text{constr.}} \rho - \frac{\partial f}{\partial \varphi_i} \left.\frac{\partial H}{\partial \theta_i}\right|_{\text{constr.}} \rho + T \int \left(\frac{\partial}{\partial \theta_i} + \frac{\partial}{\partial \varphi_i} \right)^2 f \rho \right].$$

After integrating the right-hand side of this equation by parts and plugging $\partial_{\varphi_i} H|_{\text{constr.}} = -\partial_{\theta_i} H|_{\text{constr.}}$ into the above equation, we have

$$\int \prod_j \mathrm{d}\theta_j \mathrm{d}\varphi_j f \frac{\partial \rho}{\partial t} = \int \prod_j \mathrm{d}\theta_j \mathrm{d}\varphi_j f \sum_i \left[\left(\frac{\partial}{\partial \theta_i} + \frac{\partial}{\partial \varphi_i} \right) \left(\left.\frac{\partial H}{\partial \theta_i}\right|_{\text{constr.}} \rho \right) + T \left(\frac{\partial}{\partial \theta_i} + \frac{\partial}{\partial \varphi_i} \right)^2 \rho \right]. \tag{S.133}$$

Since this result has to hold for all test functions $f(\theta_i(t), \varphi_i(t), t)$, we find

$$\frac{\partial \rho}{\partial t} = \sum_i \left[\left(\frac{\partial}{\partial \theta_i} + \frac{\partial}{\partial \varphi_i} \right) \left(\frac{\partial H}{\partial \theta_i}\bigg|_{\text{constr.}} \rho \right) + T \left(\frac{\partial}{\partial \theta_i} + \frac{\partial}{\partial \varphi_i} \right)^2 \rho \right]. \tag{S.134}$$

This Fokker-Planck equation, consistent with Eq. (S.117), governs the dynamics of the phase-space distribution. As expected, for a stationary distribution, $\partial_t \rho = 0$, it reduces to Eq. (S.129).

3. Eliminating the auxiliary degree of freedom from Langevin dynamics

When studying the nonreciprocal Langevin dynamics, it is often convenient to keep only the original angles $\vec{\theta}$ and not track the auxiliary variables $\vec{\varphi}$ explicitly. In this reduced description, the dynamics is written directly in terms of the (generally nonreciprocal) drift force $F_i(\vec{\theta})$ acting on θ_i.

We start from the overdamped Langevin equation (Itô convention)

$$\theta_i(t + \Delta t) - \theta_i(t) = F_i(\vec{\theta})\, \Delta t + \sqrt{2T}\, \Delta W_i(t), \tag{S.135}$$

where ΔW_i are Wiener increments with $\langle \Delta W_i \rangle = 0$ and $\langle \Delta W_i\, \Delta W_j \rangle = \delta_{ij} \Delta t$. The corresponding configuration-space probability density $\rho(\theta_i, t)$ obeys the standard Fokker–Planck equation

$$\partial_t \rho(\vec{\theta}, t) = -\sum_i \partial_{\theta_i} \left[F_i(\vec{\theta})\, \rho(\vec{\theta}, t) \right] + T \sum_i \partial_{\theta_i}^2 \rho(\vec{\theta}, t). \tag{S.136}$$

In a stationary state, $\partial_t \rho = 0$, and we obtain

$$0 = \sum_i \partial_{\theta_i} \left[F_i(\vec{\theta})\, \rho(\vec{\theta}) + T\, \partial_{\theta_i} \rho(\vec{\theta}) \right]. \tag{S.137}$$

For the vision-cone XY model in Eq. (S.114), the drift force is

$$F_i(\vec{\theta}) = -\sum_{j \in \langle ij \rangle} J_{ij}(\theta_i)\, \sin\!\big(\theta_i - \theta_j\big). \tag{S.138}$$

This drift force is precisely the one obtained from the Hamiltonian embedding once the constraint $\varphi_i = \theta_i + \pi$ is imposed:

$$F_i(\vec{\theta}) = \partial_{\varphi_i} H\Big|_{\text{constr.}} = -\partial_{\theta_i} H\Big|_{\text{constr.}}. \tag{S.139}$$

Moreover, the higher-order derivatives of $F_i(\vec{\theta})$ with respect to θ_i can be expressed directly in terms of the constrained Hamiltonian:

$$\partial_{\theta_i}^n F_i(\vec{\theta}) = -\partial_{\theta_i} \left(\partial_{\theta_i} + \partial_{\varphi_i} \right)^n H\Big|_{\text{constr.}}, \qquad n = 1, 2, \ldots. \tag{S.140}$$

Thus, we can always describe the force F_i and its higher-order derivatives with respect to θ_i using the Hamiltonian embedding. The steady-state distribution and non-stationary dynamics of the density introduced in Eq. (S.129) and Eq. (S.134) is recovered by expressing all derivatives of $\rho(\vec{\theta})$ in Eq. (S.136) and Eq. (S.137), $\partial_{\theta_i}^n \rho(\vec{\theta})$, in terms of $(\partial_{\theta_i} + \partial_{\varphi_i})^n \rho(\vec{\theta}, \vec{\varphi})$. This shows that the Langevin dynamics obtained from Hamiltonian embedding on the constrained manifold and the conventional nonreciprocal Langevin description give the same evolution for the physical degrees of freedom.

B. Glauber dynamics description

We now present a Glauber algorithm that samples the steady state and the nonstationary distribution of the Langevin dynamics using the Hamiltonian embedding. In particular, we focus on defining transition rates that are consistent with the constraint.

When the Hamiltonian is evaluated on the constraint, it vanishes identically, $H(\vec{\theta}, \vec{\varphi})\big|_{\text{constr.}} = 0$. Thus, it is nontrivial to define the Glauber update rule based on energy differences alone. To address this, we consider a Markov process in which every state $\{\vec{\theta}, \vec{\varphi}\}$ satisfies the constraint $\theta_i = \varphi_i + \pi$ while the transition rates are defined based on the energy difference between two states that differ by a small increment δ in the angle of the original spin and the auxiliary spin, respectively.

We show that taking δ sufficiently small guarantees that the Glauber and Langevin steady states coincide. We emphasize that, in the strict limit $\delta \to 0$, the Hamiltonian vanishes identically under the constraint, making the update rule ill-defined. However, for any small but finite δ, the Glauber dynamics correctly reproduces the physics of the nonreciprocal Langevin equation.

We start from the probability current from state $\{\vec{\theta}, \vec{\varphi}\}$ to state $\{\theta_i + \delta, \{\vec{\theta}\}_{j\neq i}, \varphi_i + \delta, \{\vec{\varphi}\}_{j\neq i}\}$, which is given by

$$\begin{aligned}\mathcal{J}(\theta_i, \varphi_i \to \theta_i + \delta, \varphi_i + \delta) = \rho(\vec{\theta}, \vec{\varphi}) w(\theta_i, \varphi_i \to \theta_i + \delta, \varphi_i + \delta) \\ - \rho(\theta_i + \delta, \{\vec{\theta}\}_{j\neq i}, \varphi_i + \delta, \{\vec{\varphi}\}_{j\neq i}) w(\theta_i + \delta, \varphi_i + \delta \to \theta_i, \varphi_i).\end{aligned} \tag{S.141}$$

Here the increment δ is restricted to the interval $[-\Delta, \Delta]$. The Glauber transition rates read as

$$w\left(\theta_i \to \theta_i', \varphi_i \to \varphi_i'\right) = \frac{1}{2}\left(1 - \tanh\left(\frac{H(\theta', \varphi) - H(\theta, \varphi')}{4T}\right)\right), \tag{S.142}$$

where the constraint is built-in. In other words, we compare the change in energy resulting from the update of each variable θ, φ, separately. In particular, we change the angle of the original spin as $\theta \to \theta' = \theta + \delta$. The resulting energy difference between the two states is given by $H(\theta_i', \varphi_i) - H(\theta_i, \varphi_i)$. Next, we compute the energy difference when the corresponding auxiliary spin is changed under the constraint, i.e., $\varphi \to \varphi' = \varphi + \delta$, with the energy difference $H(\theta_i, \varphi_i') - H(\theta_i, \varphi_i)$. This gives a total change of $H(\theta', \varphi) - H(\theta, \varphi')$.

Notice the additional factor of $1/2$ in the argument of the tanh-function, which accounts for the update of both degrees of freedom; it serves (i) to derive the correct equation for the phase-space distribution and time-dependent dynamics, cf. Eq. (S.148) and Eq. (S.155), respectively, and (ii) to reproduce the correct rates in the reciprocal limit (cf. Sec. S4 B 3 below).

The detailed balance condition in Eq. (S.115) requires that the probability current vanishes on every channel, i.e., $\mathcal{J}(\theta_i, \varphi_i \to \theta_i + \delta, \varphi_i + \delta) = 0$ in Eq. (S.141) for every $\delta \in [-\Delta, \Delta]$. However, this condition is generally not satisfied for nonreciprocal systems, which break time-reversal symmetry. Therefore, in Sec S4 B 1, we derive the steady-state distribution of the Glauber dynamics based on the global balance and then consider the time-dependent distribution in Sec. S4 B 2 when the global balance condition is not satisfied.

1. Steady-state distribution from global balance

The steady-state distribution is derived from the balance of total probability flux into and out of a given configuration, which constitutes the so-called global balance [21]

$$\sum_i \int_{-\pi}^{\pi} \mathrm{d}\delta \left[\mathcal{J}(\theta_i, \varphi_i \to \theta_i + \delta, \varphi_i + \delta) - \mathcal{J}(\theta_i + \delta, \varphi_i + \delta \to \theta_i, \varphi_i)\right]\Big|_{\text{constr.}} = 0. \tag{S.143}$$

In detail, it reads as

$$\begin{aligned}\sum_i \int_{-\pi}^{\pi} \mathrm{d}\delta \big[\rho(\theta_i + \delta, \{\vec{\theta}\}_{j\neq i}, \varphi_i + \delta, \{\vec{\varphi}\}_{j\neq i}) W(\theta_i + \delta, \varphi_i + \delta \to \theta_i, \varphi_i) \\ - \rho(\vec{\theta}, \vec{\varphi}) W(\theta_i, \varphi_i \to \theta_i + \delta, \varphi_i + \delta)\big]\Big|_{\text{constr.}} = 0.\end{aligned} \tag{S.144}$$

Here, we use $W(\theta_i + \delta, \varphi_i + \delta \to \theta_i, \varphi_i) = w(\theta_i + \delta, \varphi_i + \delta \to \theta_i, \varphi_i)\, \mathbf{1}_{[-\Delta,\Delta]}$, where $\mathbf{1}$ is the indicator function which restricts the range of the shift δ to the interval $[-\Delta, \Delta]$. In the Glauber scheme, the proposed angle increment δ is drawn independently at each update step. Consequently, to enforce global balance, one must sum over all lattice sites i and integrate over all proposed shifts δ in Eq. (S.144). This guarantees that we have considered all probability flux channels.

For small $\delta \in [-\Delta, \Delta]$ we can approximate Eq. (S.144) as

$$\begin{aligned}\sum_i \int_{-\Delta}^{\Delta} \Bigg\{ \left[\rho(\theta_i, \varphi_i) + \delta \times \left(\partial_{\theta_i} + \partial_{\varphi_i}\right) \rho(\vec{\theta}, \vec{\varphi}) + \frac{1}{2}\delta^2 \times \left(\partial_{\theta_i} + \partial_{\varphi_i}\right)^2 \rho(\vec{\theta}, \vec{\varphi})\right] \\ \times \frac{1}{2}\left(1 - \tanh \frac{-2\left(\partial_{\theta_i} H - \partial_{\varphi_i} H\right) \times \delta - \left(\partial_{\theta_i}^2 H - \partial_{\varphi_i}^2 H\right) \times \delta^2}{8T}\right) \\ - \rho(\vec{\theta}, \vec{\varphi}) \frac{1}{2}\left(1 + \tanh \frac{-2\left(\partial_{\theta_i} H - \partial_{\varphi_i} H\right) \times \delta - \left(\partial_{\theta_i}^2 H - \partial_{\varphi_i}^2 H\right) \times \delta^2}{8T}\right) \Bigg\}\Bigg|_{\text{constr.}} \mathrm{d}\delta = 0.\end{aligned} \tag{S.145}$$

Next, we perform a Taylor expansion in δ of the above equation. The first-order contribution vanishes immediately due to the symmetry of the integrand under $\delta \to -\delta$:

$$\sum_i \frac{1}{2}\Big\{T\left(\partial_{\theta_i}+\partial_{\varphi_i}\right)\rho(\vec{\theta},\vec{\varphi})+\frac{1}{2}\rho(\vec{\theta},\vec{\varphi})\left(\partial_{\theta_i}H-\partial_{\varphi_i}H\right)\Big\}\Big|_{\text{constr.}}\int_{-\Delta}^{\Delta}\delta\,\mathrm{d}\delta=0. \tag{S.146}$$

Hence, the steady-state distribution always satisfies global balance to order $O(\delta)$.

The second-order contribution in δ does not vanish. Multiplying by T, we have

$$\sum_i \frac{1}{4}\Big\{\left(\partial_{\theta_i}+\partial_{\varphi_i}\right)\Big[T\left(\partial_{\theta_i}+\partial_{\varphi_i}\right)\rho(\vec{\theta},\vec{\varphi})+\frac{1}{2}\rho(\vec{\theta},\vec{\varphi})\left(\partial_{\theta_i}H-\partial_{\varphi_i}H\right)\Big]\Big\}\Big|_{\text{constr.}}\int_{-\Delta}^{\Delta}\delta^2\mathrm{d}\delta=0. \tag{S.147}$$

Rearranging and cancelling the constants, we arrive at the following equation for the phase-space distribution function ρ

$$\sum_i\left(\partial_{\theta_i}+\partial_{\varphi_i}\right)\Big[T\left(\partial_{\theta_i}+\partial_{\varphi_i}\right)\rho(\vec{\theta},\vec{\varphi})+\rho(\vec{\theta},\vec{\varphi})\partial_{\theta_i}H\Big]=0. \tag{S.148}$$

We find that Eq. (S.148) is equivalent to Eq. (S.129), after using the relation $-\partial_{\varphi_i}H=\partial_{\theta_i}H$ which is valid under the constraint.

2. *Non-stationary distributions from Monte-Carlo Glauber dynamics*

As we have seen, the constrained Glauber dynamics can generate nonequilibrium steady-state distributions through the constraints imposed at each update step. Hence, it is natural to ask whether one can also use Glauber dynamics to obtain the non-stationary states of the original Langevin dynamics. This will enable the application of the Hamiltonian embedding to study non-stationary states of nonreciprocal systems, which can feature configuration-space entropy currents and time-translation symmetry breaking [24]. In particular, we now show that the Glauber dynamics defined by our Hamiltonian embedding reproduces the Fokker–Planck equation derived for the Langevin dynamics in Eq. (S.134)

We denote the density at iteration step n by $\rho(\vec{\theta},\vec{\varphi},n)$, and recall the corresponding transition rate w, defined in Eq. (S.142). In each update step, the probability of selecting site j is uniformly given by $1/L^2$ with L the linear system size; the shift angle δ is drawn uniformly from the interval $[-\Delta,\Delta]$, with probability density $1/(2\Delta)$. Sampling under the constraint is reminiscent of directed loop algorithms, which are used to explore the extensive discrete set of ground states in spin models supporting an emergent gauge theory [25], and more efficient algorithms may exist.

After a single Glauber update $n\to n+1$, the change in the density is given by

$$\begin{aligned}\rho(\vec{\theta},\vec{\varphi},n+1)-\rho(\vec{\theta},\vec{\varphi},n)=\frac{1}{2\Delta L^2}\sum_i\int_{-\Delta}^{\Delta}\mathrm{d}\delta\Big[&\rho(\theta_i+\delta,\{\vec{\theta}\}_{i\neq j},\varphi_i+\delta,\{\vec{\varphi}\}_{i\neq j},n)\;w(\theta_i+\delta\to\theta_i,\varphi_i+\delta\to\varphi_i)\\&-\rho(\theta_i,\{\vec{\theta}\}_{i\neq j},\varphi_i,\{\vec{\varphi}\}_{i\neq j},n)\;w(\theta_i\to\theta_i+\delta,\varphi_i\to\varphi_i+\delta)\Big].\end{aligned} \tag{S.149}$$

The right-hand side represents the difference between the total inflow and outflow of density current for the Glauber update. As before, in the limit of small Δ, it can be approximated by expanding both the transition rates and the density up to second order in δ:

$$\begin{aligned}\rho(\vec{\theta},\vec{\varphi},n+1)-\rho(\vec{\theta},\vec{\varphi},n)=\frac{1}{2\Delta L^2}\sum_i\times&\\\int_{-\Delta}^{\Delta}\mathrm{d}\delta\Bigg\{&\Big[\rho(\vec{\theta},\vec{\varphi},n)+\delta\times\left(\partial_{\theta_i}+\partial_{\varphi_i}\right)\rho(\vec{\theta},\vec{\varphi},n)+\frac{1}{2}\delta^2\times\left(\partial_{\theta_i}+\partial_{\varphi_i}\right)^2\rho(\vec{\theta},\vec{\varphi},n)\Big]\\&\times\frac{1}{2}\left(1-\tanh\frac{-2\left(\partial_{\theta_i}H-\partial_{\varphi_i}H\right)\times\delta-\left(\partial^2_{\theta_i}H-\partial^2_{\varphi_i}H\right)\times\delta^2}{8T}\right)\\&-\rho(\vec{\theta},\vec{\varphi},n)\frac{1}{2}\left(1+\tanh\frac{-2\left(\partial_{\theta_i}H-\partial_{\varphi_i}H\right)\times\delta-\left(\partial^2_{\theta_i}H-\partial^2_{\varphi_i}H\right)\times\delta^2}{8T}\right)\Bigg\}\Bigg|_{\text{constr.}}.\end{aligned} \tag{S.150}$$

As expected, for stationary distributions where $\rho(\vec{\theta}, \vec{\varphi}, n)$ is time independent, the equation reduces to Eq. (S.145) discussed in Sec. S6 D, except for the overall prefactor $1/(2\Delta L^2)$. Similar to the previous section, we perform a Taylor expansion of the above equation and retain terms up to second order in δ. This yields

$$\begin{aligned}\rho(\vec{\theta}, \vec{\varphi}, n+1) - \rho(\vec{\theta}, \vec{\varphi}, n) = \sum_i \frac{1}{2\Delta L^2} \int_{-\Delta}^{\Delta} \mathrm{d}\delta \Bigg\{ \delta \Big[\frac{1}{2}(\partial_{\theta_i} + \partial_{\varphi_i})\rho(\vec{\theta}, \vec{\varphi}, n) + \frac{1}{4T}\rho(\vec{\theta}, \vec{\varphi}, n)(\partial_{\theta_i} - \partial_{\varphi_i})H \Big] \\ + \delta^2 \frac{1}{4T}(\partial_{\theta_i} + \partial_{\varphi_i}) \Big[\frac{1}{2}\rho(\vec{\theta}, \vec{\varphi}, n)(\partial_{\theta_i} - \partial_{\varphi_i})H + T(\partial_{\theta_i} + \partial_{\varphi_i})\rho(\vec{\theta}, \vec{\varphi}, n) \Big] \Bigg\} \Bigg|_{\text{constr.}} . \end{aligned} \tag{S.151}$$

Upon integration, the first-order term in δ vanishes owing to the symmetry of the integrand. Hence, performing the integration over δ for the second-order term, we obtain

$$\rho(\vec{\theta}, \vec{\varphi}, n+1) - \rho(\vec{\theta}, \vec{\varphi}, n) = \sum_i \frac{\Delta^2}{12L^2T}(\partial_{\theta_i} + \partial_{\varphi_i}) \Big[\rho(\vec{\theta}, \vec{\varphi}, n)\partial_{\theta_i} H\big|_{\text{constr.}} + T(\partial_{\theta_i} + \partial_{\varphi_i})\rho(\vec{\theta}, \vec{\varphi}, n) \Big] + O(\Delta^4), \tag{S.152}$$

where we used the equality $\partial_{\theta_i} H\big|_{\text{constr.}} = -\partial_{\varphi_i} H\big|_{\text{constr.}}$ valid on the constraint manifold. By the same symmetry arguments, the leading order correction is of the order $O(\Delta^4)$.

In order to compare the Glauber simulations to real-time Langevin dynamics in practice, we now introduce the notion of Monte-Carlo time, t_{MC}, for the constrained Glauber dynamics. To avoid confusion, the time variable t used outside this section is identified as the Langevin time and is here relabeled as t_{Lan}; this is the proper physical time from the equations of motion of the original nonreciprocal system. We associate one unit of (dimensionless) Monte-Carlo time with updating each spin once (on average), and hence define

$$t_{\mathrm{MC}} = \frac{n}{L^2}. \tag{S.153}$$

Accordingly, we identify the discrete difference with

$$\rho(\vec{\theta}, \vec{\varphi}, n+1) - \rho(\vec{\theta}, \vec{\varphi}, n) = \partial_{t_{\mathrm{MC}}}\rho(\theta_i, \varphi_i, t_{\mathrm{MC}})\, \mathrm{d}t_{\mathrm{MC}}, \tag{S.154}$$

with $\mathrm{d}t_{\mathrm{MC}} = 1/L^2$. This allows us to formally define a continuum limit for the Monte-Carlo time.

Following the derivation above, the equation of motion for the density distribution then reads as

$$\frac{\partial\rho(\vec{\theta}, \vec{\varphi}, t_{\mathrm{MC}})}{\partial t_{\mathrm{MC}}} = \frac{\Delta^2}{12T}\sum_i (\partial_{\theta_i} + \partial_{\varphi_i}) \Big[\rho\partial_{\theta_i} H\big|_{\text{constr.}} + T(\partial_{\theta_i} + \partial_{\varphi_i})\rho \Big] + O(\Delta^4). \tag{S.155}$$

This equation is equivalent to the Fokker-Planck equation derived for the Langevin dynamics in Eq. (S.134), up to the prefactor $\Delta^2/(12T)$. In units where the friction coefficient is set to unity, it follows from the equations of motion in Eq. (S.55) that the interaction strength J, and hence energy, has the same unit as frequency (inverse time). Since the only model-independent energy scale in the Glauber rate w is the temperature T (which also has units of energy), we find that the time unit is set by the inverse bath temperature T. The dimensionless factor $\Delta^2/12$ is a property of the Glauber update rule, and is most likely not universal.

These results demonstrate that the constrained Glauber dynamics reproduce the same non-stationary evolution as the Langevin dynamics as $\Delta \to 0$, provided the Monte-Carlo time t_{MC} is related to the physical Langevin time t_{Lan} through a proportionality constant, namely

$$\frac{\Delta^2}{12T} t_{\mathrm{MC}} = t_{\mathrm{Lan}}. \tag{S.156}$$

Physically, this relation implies that the lower the bath temperature T, the fewer Monte-Carlo updates are necessary to reach the same unit of physical Langevin time at fixed Δ. Moreover, since the constrained Glauber update rule reproduces the nonreciprocal physics in the limit $\Delta \ll 1$ and because the next-order correction in Eq. (S.155) is Δ^4, there is a natural timescale set by $1/\Delta^2$ (at a fixed temperature T), beyond which Glauber dynamics is expected to disagree with Langevin dynamics; we emphasize that this scale can be controllably extended to an arbitrarily long Langevin time by decreasing Δ which, however, requires increasing the number of Glauber iterations n.

We provide numerical evidence in support of Eq. (S.156) in Fig. S8 and Sec. S7.

3. Eliminating the auxiliary degree of freedom from the Glauber transition rates

Whereas the nonreciprocal equations of motion of interest depend only on the original degrees of freedom θ, the Glauber update rules derived in the preceding subsections appear to depend on both the original and the auxiliary degrees of freedom: $w\left(\theta_i \to \theta_i', \varphi_i \to \varphi_i'\right)$; this is because the Glauber transition rate w was derived using the Hamiltonian embedding. As a result, one might be tempted to argue that the constrained Glauber Monte-Carlo simulations are computationally more expensive than the corresponding original Langevin dynamics.

Here, we show that this is not the case: one can eliminate the auxiliary field φ completely from the expression for the constrained Glauber transition rate: $w = w(\theta_i \to \theta_i')$. To get an intuition for why this is the case, notice that, in constrained Glauber dynamics, observables depend only on the original spins θ, while the values of the auxiliary spins φ are fixed by the constraint. As a result, the Monte-Carlo fluctuations of φ are slaved to those of θ at each Glauber step.

For clarity, we apply the procedure to the vision-cone XY model using the Hamiltonian in Eq. (S.114). First, we initialize the simulation in a (θ_i, φ_i)-configuration that obeys the constraint. Hence, at the start of each Glauber update step, the constraint $\varphi_i = \theta_i + \pi$ holds, so the auxiliary angle can be expressed in terms of θ_i. We then update $\theta_i \to \theta_i' = \theta_i + \delta$ and $\varphi_i \to \varphi_i' = \varphi_i + \delta$, which preserves the constraint manifold: $\varphi_i' = \theta_i' + \pi$. The key observation is that φ_i' may be replaced by $\theta_i' + \pi$ when evaluating the energy differences which define the Glauber transition rate.

To see this in practice, we will revisit the expression for the transition rate from Eq. (S.142). The energy difference in the argument of the hyperbolic tangent can be expressed as

$$\begin{aligned}\Delta E &= \frac{1}{2}\big(H(\theta',\varphi) - H(\theta,\varphi')\big)\Big|^{\varphi=\theta+\pi}_{\varphi'=\theta'+\pi} \\ &= \frac{1}{2}\big(H(\theta',\varphi) - H(\theta,\varphi) + H(\theta,\varphi) - H(\theta,\varphi')\big)\Big|^{\varphi=\theta+\pi}_{\varphi'=\theta'+\pi} \\ &= \frac{1}{2}\left(\Delta E_1\Big|_{\varphi=\theta+\pi} - \Delta E_2\Big|^{\varphi=\theta+\pi}_{\varphi'=\theta'+\pi}\right),\end{aligned} \tag{S.157}$$

where, crucially, the constraint is applied only after the differences are evaluated.

Now consider the term ΔE_1, and note that the energy change associated with updating the original spin θ_i, while keeping φ_i fixed, is given by

$$\begin{aligned}\Delta E_1 &= H(\theta',\varphi) - H(\theta,\varphi) \\ &= -\sum_{j\in\langle ij\rangle}\Big\{[J_{ij}(\theta_i') + J_{ji}(\theta_j)]\cos\big(\theta_i' - \theta_j\big) - [J_{ij}(\theta_i) + J_{ji}(\theta_j)]\cos\big(\theta_i - \theta_j\big)\Big\} \\ &\quad - \sum_{j\in\langle ij\rangle}\Big\{[J_{ij}(\theta_i') - J_{ij}(\theta_i)]\cos\big(\theta_j - \varphi_i\big) + J_{ji}(\theta_j)[\cos\big(\theta_i' - \varphi_j\big) - \cos\big(\theta_i - \varphi_j\big)]\Big\}.\end{aligned} \tag{S.158}$$

Since the Glauber update depends only on the energy difference, the auxiliary angles can be eliminated using the constraint $\varphi = \theta + \pi$, without changing the transition rate. Substituting $\varphi = \theta + \pi$ gives

$$\begin{aligned}\Delta E_1\Big|_{\varphi=\theta+\pi} &= H(\theta',\theta+\pi) - H(\theta,\theta+\pi) \\ &= -\sum_{j\in\langle ij\rangle}\Big\{[J_{ij}(\theta_i') + J_{ji}(\theta_j)]\cos\big(\theta_i' - \theta_j\big) - [J_{ij}(\theta_i) + J_{ji}(\theta_j)]\cos\big(\theta_i - \theta_j\big)\Big\} \\ &\quad + \sum_{j\in\langle ij\rangle}[J_{ij}(\theta_i') - J_{ij}(\theta_i)]\cos\big(\theta_j - \theta_i\big) + \sum_{j\in\langle ij\rangle}J_{ji}(\theta_j)[\cos\big(\theta_i' - \theta_j\big) - \cos\big(\theta_i - \theta_j\big)] \\ &= -\sum_{j\in\langle ij\rangle}J_{ij}(\theta_i')[\cos\big(\theta_i' - \theta_j\big) - \cos\big(\theta_i - \theta_j\big)].\end{aligned} \tag{S.159}$$

Likewise, the energy difference associated with updating the auxiliary spin φ_i while keeping θ_i fixed reads as

$$\Delta E_2 = H(\theta,\varphi') - H(\theta,\varphi) = -\sum_{j\in\langle ij\rangle}J_{ij}(\theta_i)[\cos\big(\theta_j - \varphi_i'\big) - \cos\big(\theta_j - \varphi_i\big)]. \tag{S.160}$$

Applying the constraint by substituting $\varphi' = \theta' + \pi$ and $\varphi = \theta + \pi$ yields

$$\Delta E_2\Big|^{\varphi=\theta+\pi}_{\varphi'=\theta'+\pi} = H(\theta,\theta'+\pi) - H(\theta,\theta+\pi) = \sum_{j\in\langle ij\rangle}J_{ij}(\theta_i)[\cos\big(\theta_i' - \theta_j\big) - \cos\big(\theta_i - \theta_j\big)]. \tag{S.161}$$

Therefore, the total energy difference entering the transition rate in Eq. (S.142) simplifies to

$$\Delta E = \frac{1}{2}\left(\Delta E_1\big|_{\varphi=\theta+\pi} - \Delta E_2\big|^{\varphi=\theta+\pi}_{\varphi'=\theta'+\pi}\right)$$
$$= -\frac{1}{2}\sum_{j\in\langle ij\rangle}[J_{ij}(\theta_i') + J_{ij}(\theta_i)][\cos(\theta_i' - \theta_j) - \cos(\theta_i - \theta_j)]. \quad \text{(S.162)}$$

Consequently, the joint-update constrained Glauber dynamics can be implemented using only the original variables θ_i, and the corresponding transition rate is

$$w(\theta_i \to \theta_i') = \frac{1}{2}\left(1 - \tanh\frac{\Delta E}{2T}\right), \quad \text{(S.163)}$$

with ΔE given by Eq. (S.162).

Notably, in the reciprocal limit $\Psi=\pi$, the interaction becomes orientation-independent: $J_{ij}(\theta_i') = J_{ij}(\theta_i) = J_0$. Then $\Delta E = -J_0\sum_{j\in\langle ij\rangle}\left[\cos(\theta_i' - \theta_j) - \cos(\theta_i - \theta_j)\right]$, which matches exactly the energy difference obtained from the conventional XY Hamiltonian $H_{\mathrm{XY}} = -J_0\sum_{\langle ij\rangle}\cos(\theta_i - \theta_j)$ (and hence justifies the factor of $1/2$ in the definition of ΔE above a posteriori). Thus, although imposing the constraint breaks detailed balance in general, the latter is restored in the reciprocal limit, as expected.

As a consequence of eliminating the auxiliary degrees of freedom φ, the total number of XY-spins to be simulated in the constrained Glauber algorithm is equivalent to that used in the original Langevin dynamics. Note that we proved this elimination procedure only for the constrained Glauber dynamics considered in this work; further research is required to determine whether, and under which conditions, it extends to other energy-difference-based algorithms.

Finally, let us emphasize that the role of the Hamiltonian embedding here is to provide the correct Glauber transition rate (which we have proved to give rise to the correct Fokker-Planck equation in the limit of $\delta \to 0$, cf. Sec. S6 D).

S5. DETERMINING THE CRITICAL TEMPERATURE FOR THE VISION-CONE MODEL

To determine the critical temperature within the statistical uncertainty, $T_c \pm \Delta T_c$, for a given algorithm, we first conduct N independent simulations (runs) of the dynamics for fixed values T of the reservoir temperature. For each run i, we determine the transition temperature $T_{c,i}$ from the peak of the specific heat. The mean and standard deviation over the N independent runs are subsequently computed from

$$T_c = \frac{1}{N}\sum_{i=1}^{N} T_{c,i}, \qquad \Delta T_c = \frac{1}{N}\sqrt{\sum_{i=1}^{N}\left(T_{c,i} - T_c\right)^2}. \quad \text{(S.164)}$$

S6. SUPPLEMENTARY RESULTS FOR THE MONTE-CARLO SIMULATIONS

This section presents supplementary results for the Monte-Carlo simulations presented in the Main Text. In Sec. S6 A, we detail the implementation of the Glauber algorithm under the proposed constraints, derived in Sec. S6 D. In Sec. S6 C, we present numerical results for the individual-update Glauber dynamics that we propose, for Glauber dynamics with selfish energy as proposed in previous works [26–28], and for Langevin dynamics across various system sizes. Additionally, we examine the outcomes of Glauber dynamics with the Hamiltonian dropping the auxiliary spin (Eq. (S.169)), and highlight the significant differences from the behavior observed in the constrained Glauber dynamics. Finally, in Sec. S6 E, we explore a different vision-cone interaction strength proposed in Ref. [29] and compare the constrained Glauber dynamics with selfish energy dynamics [29] and Langevin dynamics: We find numerically identical behavior obtained from the constrained Glauber dynamics and the Langevin dynamics, whereas the selfish energy approach results in a different behavior.

A. Constrained Glauber algorithm

In this subsection, we introduce the details of the constrained Glauber algorithm from Sec. S4 B that we use in the Main Text. The Hamiltonian is introduced in Eq. (S.114). We use $\Psi^{(n)}$ to represent the system configuration $\Psi^{(n)} = (\theta_1^{(n)}, \theta_2^{(n)}, \ldots\theta_i^{(n)}, \ldots\theta_N^{(n)}, \varphi_1^{(n)}, \varphi_2^{(n)}, \ldots\varphi_i^{(n)}, \ldots\varphi_N^{(n)})$, where n denotes the iteration step of the Monte-Carlo simulation. The algorithm proceeds as follows:

1. **Initial Configuration:** Set initial values for the original and auxiliary spins in a uniform configuration, with $\theta_i^{(0)} = 0$ and $\varphi_i^{(0)} = \pi$. This initial configuration satisfies the embedding constraint.

2. **Original Spin Update:** Propose a new configuration by selecting an *original* spin uniformly at random and rotating it to a new angle $\theta_i^{(0)} \to \theta_i^{(0)} + \delta$, where δ is drawn from a uniform distribution between $[-\Delta, \Delta]$. As anticipated in Sec. S4 B, we find that the smaller Δ, the better the agreement with the Langevin steady state. The energy of the new configuration reads as $H(\theta_i^{(0)} + \delta, \varphi_i^{(0)})$ where H is the embedding Hamiltonian.

3. **Auxiliary Spin Update:** We also update the the auxiliary spin φ_i by the same angle δ to ensure that the constraint is preserved at each step, i.e., $\varphi_i^{(0)} \to \varphi_i^{(0)} + \delta$. The energy of this configuration is $H(\theta_i^{(0)}, \varphi_i^{(0)} + \delta)$.

4. **Energy Change Calculation:** The energy difference between the initial and the proposed configurations is given by $\Delta E = \frac{1}{2}[H(\theta_i^{(0)} + \delta, \varphi_i^{(0)}) - H(\theta_i^{(0)}, \varphi_i^{(0)} + \delta)]$. For the XY spins with vision-cone interactions, it is given by

$$\begin{aligned}\Delta E = &-\frac{1}{2} \sum_{j\in\langle ij\rangle} \Big\{ J_{ij}(\theta_i^{(0)}+\delta) \left[\cos\!\left(\theta_i^{(0)}+\delta-\theta_j^{(0)}\right) + \cos\!\left(\varphi_i^{(0)}-\theta_j^{(0)}\right)\right] \\ &\qquad + J_{ji}(\theta_j^{(0)}) \left[\cos\!\left(\theta_j^{(0)}-\theta_i^{(0)}-\delta\right) + \cos\!\left(\varphi_j^{(0)}-\theta_i^{(0)}-\delta\right)\right] \Big\} \\ &+\frac{1}{2} \sum_{j\in\langle ij\rangle} \Big\{ J_{ij}(\theta_i^{(0)}) \left[\cos\!\left(\theta_i^{(0)}-\theta_j^{(0)}\right) + \cos\!\left(\varphi_i^{(0)}+\delta-\theta_j^{(0)}\right)\right] + J_{ji}(\theta_j^{(0)}) \left[\cos\!\left(\theta_j^{(0)}-\theta_i^{(0)}\right) + \cos\!\left(\varphi_j^{(0)}-\theta_i^{(0)}\right)\right] \Big\}.\end{aligned} \tag{S.165}$$

5. **Transition Probability:** Calculate the transition probability w using the Glauber transition rates at temperature T:

$$w = \frac{1}{2}\left(1 - \tanh\frac{\Delta E}{2T}\right). \tag{S.166}$$

6. **Acceptance/Rejection Step:** To accept or reject the proposed update, draw a random number r from a uniform distribution between 0 and 1. If it is less than the transition probability, i.e., $r < w$, accept the new configuration by updating the spins on the selected site as $\theta_i^{(1)} = \theta_i^{(0)} + \delta$, and $\varphi_i^{(1)} = \varphi_i^{(0)} + \delta$. Otherwise, reject the update and leave the spin unchanged. Note that all other spins remain unchanged $\theta_j^{(1)} = \theta_j^{(0)}$, for all $j \neq i$.

7. **Repeat:** Iterate the procedure for a large number of steps. In our numerical simulations, we perform $200L^4$ iterations where L is the linear dimension.

B. Constrained Glauber algorithm after eliminating the auxiliary spins

In Sec. S4 B 3, we have shown that the auxiliary spins can be eliminated from the expression for the transition rate, and hence we can simulate the constrained Glauber dynamics using only the original spins θ_i. The system configuration is now $\Psi^{(n)} = (\theta_1^{(n)}, \theta_2, \ldots \theta_N^{(n)})$.

The algorithm proceeds as follows:

1. **Initial Configuration:** Set initial values for the original spins in a uniform configuration, with $\theta_i^{(0)} = 0$. This initial configuration satisfies the constraint.

2. **Spin Update:** Propose a new configuration by selecting a spin uniformly at random and rotating it to a new angle $\theta_i^{(0)} \to \theta_i^{(0)} + \delta$, where δ is drawn from a uniform distribution between $[-\Delta, \Delta]$.

3. **Energy Change Calculation:** The energy difference between the initial and the proposed configurations is given by Eq. (S.162), which reads as

$$\Delta E = -\frac{1}{2} \sum_{j\in\langle ij\rangle} \left[J_{ij}(\theta_i^{(0)}+\delta) + J_{ij}(\theta_i^{(0)})\right] \left[\cos\!\left(\theta_i^{(0)}+\delta-\theta_j^{(0)}\right) - \cos\!\left(\theta_i^{(0)}-\theta_j^{(0)}\right)\right]. \tag{S.167}$$

4. **Transition Probability:** Calculate the transition probability w using the Glauber transition rates at temperature T:

$$w = \frac{1}{2}\left(1 - \tanh\frac{\Delta E}{2T}\right). \tag{S.168}$$

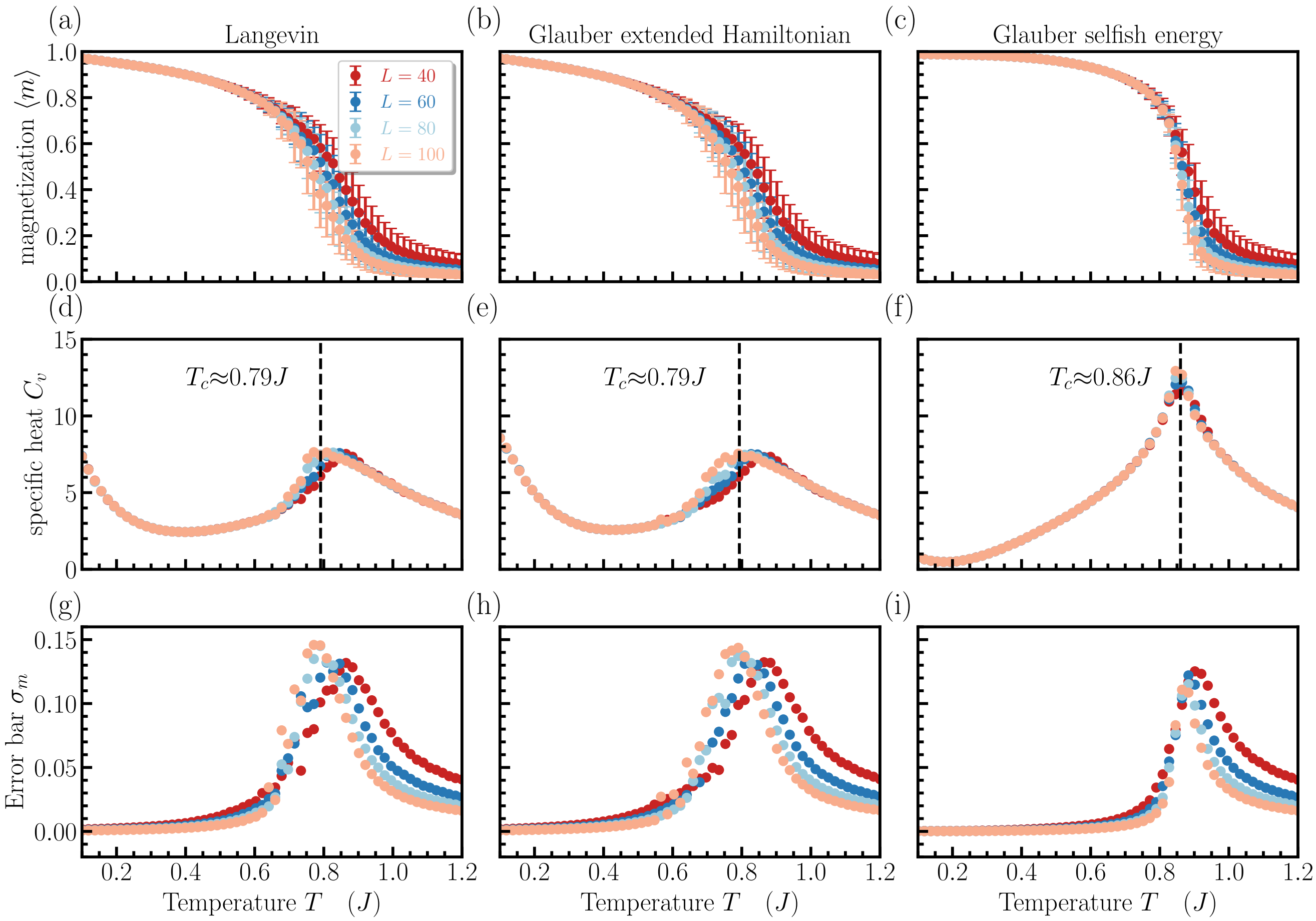

FIG. S2. Average magnetization $\langle m \rangle$ (a,b,c), specific heat C_v (d,e,f), and standard deviation of magnetization σ_m (error bars) (g,h,i) obtained from numerical simulations of Langevin dynamics, constrained Glauber dynamics, and selfish-energy Glauber dynamics as functions of temperature T. Away from the transition, the magnetization fluctuations are self-averaging, so the standard deviation decreases with increasing linear size L for all three dynamics. Close to the critical point, the peak of error bars increases with increasing L for both Langevin and constrained Glauber dynamics, whereas the selfish-energy Glauber dynamics shows decreasing error bars upon increasing L (at least up to $L = 100$). The results indicate that the selfish-energy Glauber dynamics samples a different stationary distribution rather than reproducing the critical behavior of the Langevin dynamics. We consider the vision cone angle $\Psi = 0.85\pi$. We initially start from the ordered state with angle $\theta_i = 0$. A total of $n_{\text{eq}} = 10^4$ data points are uniformly sampled at nonequilibrium steady regimes from $\mathcal{N} = 100$ independent trajectories, each initialized with a different random seed, at every temperature. The total number of samples used to compute averages and standard error bars is therefore $n = n_{\text{eq}} \times \mathcal{N} = 10^6$.

5. **Acceptance/Rejection Step:** To accept or reject the proposed new configuration, draw a random number r from a uniform distribution between 0 and 1. If it is less than the transition probability, i.e., $r < w$, accept the new configuration by updating the selected spin as $\theta_i^{(1)} = \theta_i^{(0)} + \delta$. Otherwise, reject the update and leave the spin unchanged. Note that all other spins remain unchanged $\theta_j^{(1)} = \theta_j^{(0)}$, for all $j \neq i$.

6. **Repeat:** Iterate the procedure for a large number of steps. In our numerical simulations, we perform $200L^4$ iterations where L is the linear dimension.

C. Finite-size effects

In this subsection, we investigate the effects of system size as shown in Fig. S2. The average magnetization and specific heat from both Langevin dynamics and the constrained Glauber dynamics that we propose here exhibit similar behavior across ordered and disordered phases at different system sizes, differing significantly from the results of selfish-energy Glauber dynamics proposed in previous works [26–28].

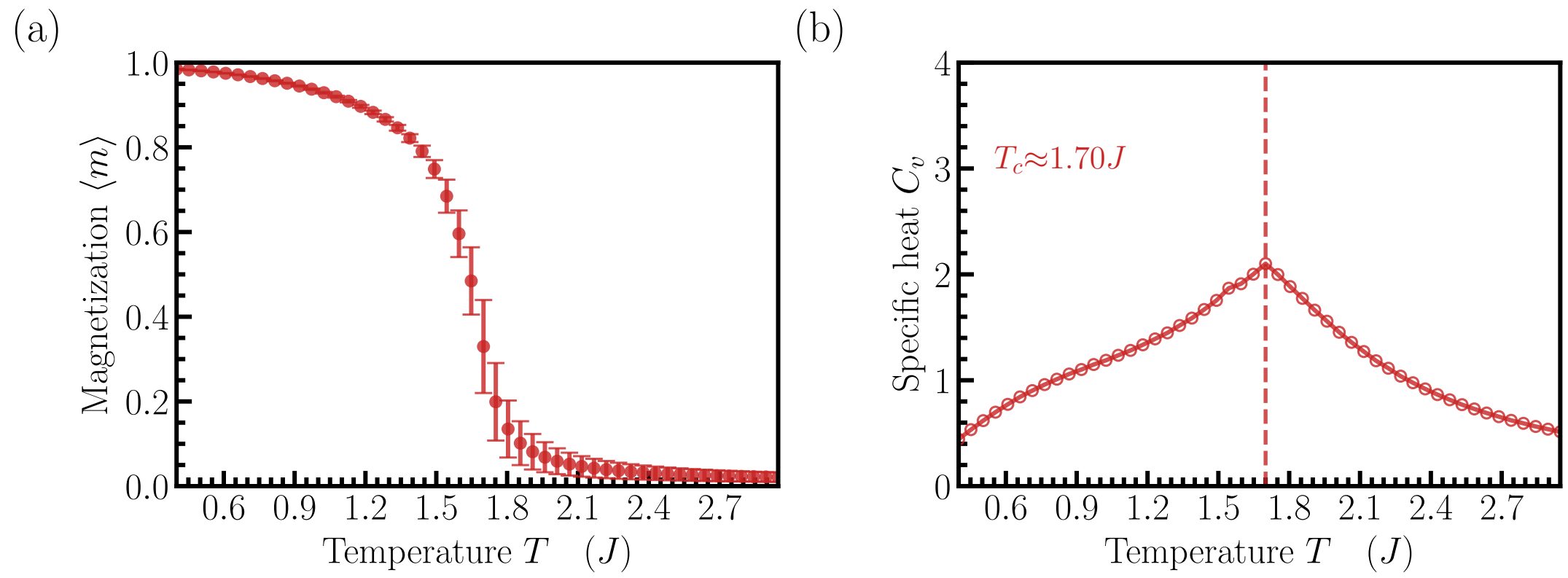

FIG. S3. Average magnetization $\langle m\rangle$, standard deviation of magnetization σ_m (error bars) (a) and specific heat C_v (b) obtained from Monte-Carlo simulations of Glauber dynamics without auxiliary spins as functions of temperature T. The critical temperature is $T_c = 1.70J$, which differs significantly from the value $T_c = 0.79J$ obtained through the constrained Glauber dynamics. We initially start from the ordered state with angle $\theta_i = 0.25\pi$. The square lattice has linear dimension $L = 100$, and the vision cone angle is $\Psi = 0.85\pi$. We perform $150L^4$ Monte-Carlo iterations. For statistical analysis, we uniformly sample $n_{\rm eq} = 10^4$ data points from the last $50L^4$ iterations of $\mathcal{N} = 100$ independent trajectories, each initialized with a distinct random seed at every temperature value. The total number of samples used for computing averages and error bars is $n = n_{\rm eq} \times \mathcal{N} = 10^6$.

To better characterize the fluctuations in the magnetization, we also examine the standard deviation $\sigma_m = \sqrt{\langle m^2\rangle - \langle m\rangle^2}$ across different system sizes (shown as error bars in the panels of the first row in Fig. S2). Both Langevin and constrained Glauber dynamics exhibit similar scaling behavior, which differs markedly from that of the selfish-energy Glauber dynamics. Away from the critical temperature, the error bars decrease with increasing system size L in all three methods, as expected due to self-averaging in large systems. However, near the critical temperature, Langevin and constrained Glauber dynamics show increasing error bars with system size, reflecting critical fluctuations that grow in the thermodynamic limit. By contrast, the selfish-energy Glauber dynamics exhibits the opposite trend, with decreasing error bars near the transition, suggesting a different critical behavior. This discrepancy further supports the usefulness of our constrained Hamiltonian embedding approach in capturing the correct nonequilibrium physics of nonreciprocal systems.

D. Glauber dynamics without auxiliary spins

To show the key role of the auxiliary spins in our Hamiltonian embedding construction, we also consider the Glauber dynamics for the Hamiltonian without auxiliary DOF

$$H_{SS} = -\sum_{\langle ij\rangle}\left[J_{ij}(\theta_i) + J_{ji}(\theta_j)\right]\cos(\theta_i - \theta_j). \tag{S.169}$$

Here, $J_{ij}(\theta_i)$ is the vision-cone interaction introduced in the Main Text.

The average magnetization and the specific heat are plotted in Fig. S3. The critical temperature is found at $T_c = 1.72J$, which differs significantly from the value of $T_c = 0.79J$ obtained from either the constrained Glauber dynamics or the original Langevin dynamics. This result exemplifies the crucial role played by the auxiliary degrees of freedom and the constraint in the Hamiltonian construction proposed in this work. Finally, consistent with the results of Ref. [30], the critical temperature for the Hamiltonian in Eq. (S.169) is nearly twice that of the value $T_c = 0.86J$ found for the selfish-energy Glauber dynamics.

E. Soft-vision-cone interactions: Comparison between selfish energy and constrained Glauber dynamics

To demonstrate that the agreement between the constrained Glauber dynamics and the Langevin dynamics is not a coincidence, we now adopt the vision-cone interaction proposed in Ref. [29] and apply it to the XY spins on the square lattice. Specifically, we consider the interaction strength

$$J_{ij}(\theta_i) = \exp\left\{\left[\kappa\cos\left(\theta_i - \psi_{ij}\right)\right]\right\}, \tag{S.170}$$

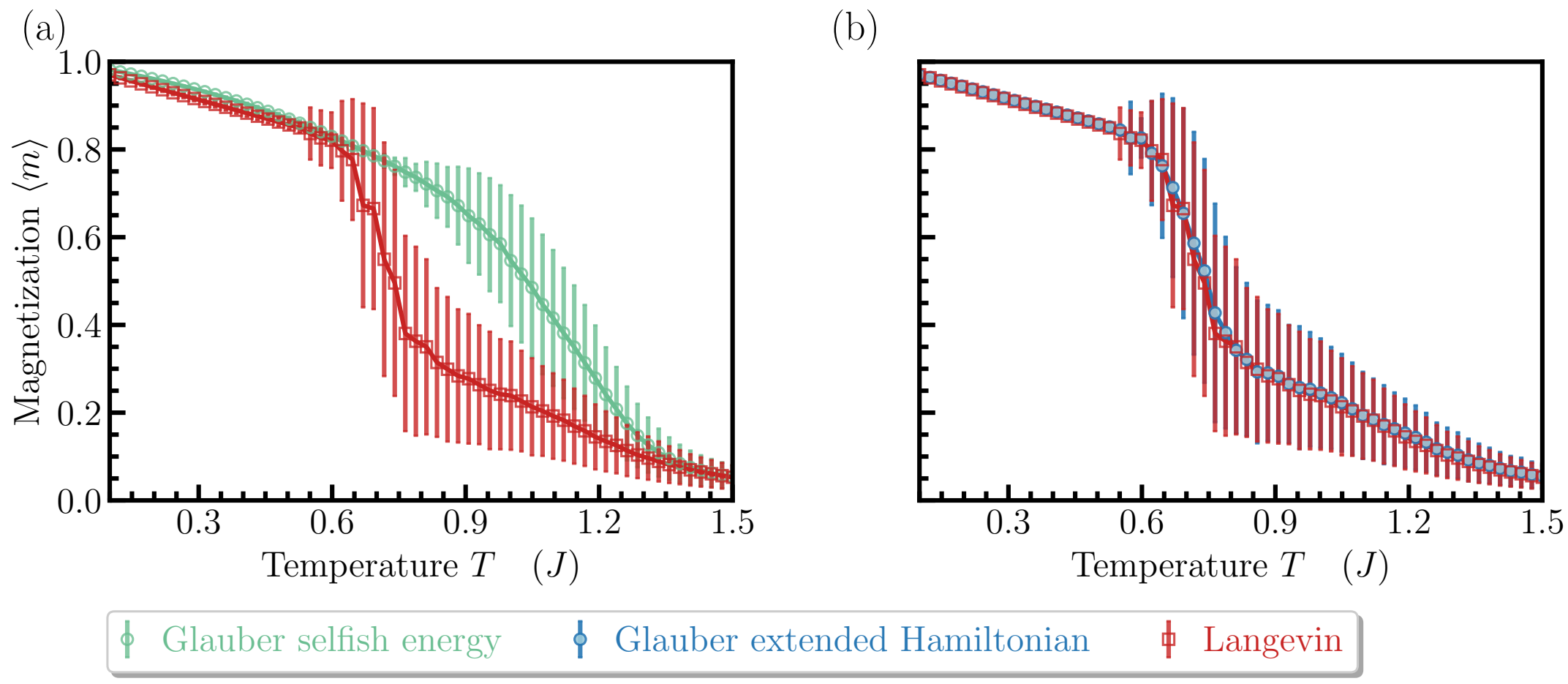

FIG. S4. Average magnetization $\langle m \rangle$ with standard deviation σ_m (error bars) in the steady state on a square lattice of linear dimension $L = 100$, as a function of temperature T for constrained Glauber (blue dots), selfish-energy Glauber (green dots), and Langevin (red squares) dynamics. The results show that in the long-time steady state, the constrained Glauber dynamics accurately reproduces the behavior of the Langevin dynamics and captures the same critical temperature, $T_c/J \approx 0.74$ (a), whereas the selfish-energy Glauber dynamics predicts a significantly different critical temperature, $T_c/J \approx 1.07$ (b). We use the vision-cone interaction strength in Eq. (S.170) with $\kappa = 1$, the semi-width $\Delta = 0.2$ for constrained Glauber dynamics, and a time step of $\delta t = 0.01$ for Langevin dynamics. We perform $200L^4$ iterations ($100L^2$ time steps) for constrained Glauber (Langevin) dynamics and $70L^4$ iterations for selfish-energy Glauber dynamics. We uniformly sample $n_{\text{eq}} = 10^4$ data points from the last $50L^4$ iterations for selfish-energy and Glauber dynamics while from $50L^2$ for Langvin dynamics. We consider $\mathcal{N} = 100$ independent trajectories, each initialized with a distinct random seed, at every temperature. The total number of samples used to compute averages and error bars is therefore $n = n_{\text{eq}} \times \mathcal{N} = 10^6$.

where ψ_{ij} is defined in the main text. The explicit form of the selfish energy [26–28] for this interaction reads as

$$E_i = -\sum_{j\in\langle ij\rangle} \exp\{[\kappa \cos(\theta_i - \psi_{ij})]\} \cos(\theta_i - \theta_j). \tag{S.171}$$

The form of the Hamiltonian embedding remains identical to Eq. (S.114) with the interaction replaced by Eq. (S.170). The Langevin equations of motion derived from the Hamiltonian under the constraint $\theta_i(t) - \varphi_i(t) = \pi$ are given by

$$\begin{aligned} \dot{\theta}_i &= -\sum_{j\in\langle ij\rangle} \exp\{[\kappa \cos(\theta_i - \psi_{ij})]\} \sin(\theta_i - \theta_j) + \sqrt{2T}\eta_i(t), \\ \dot{\varphi}_i &= \sum_{j\in\langle ij\rangle} \exp\{[\kappa \cos(\theta_i - \psi_{ij})]\} \sin(\varphi_i - \varphi_j) + \sqrt{2T}\eta_i(t). \end{aligned} \tag{S.172}$$

The average steady-state magnetization for the three alternative approaches at system size $L = 100$ is shown in Fig. S4, for $\kappa = 1$. For the constrained Glauber dynamics, we use a semi-width $\Delta = 0.2$ and $200 \times L^4$ iterations; a time step $\delta t = 0.01$ is used for Langevin dynamics.

As shown in Fig. S4, the average steady-state magnetization from selfish-energy Glauber dynamics (green dots) is consistently higher than that obtained from constrained Glauber (blue dots) and Langevin (red squares) dynamics. Importantly, the transition temperature from the ordered to the disordered state in the selfish-energy Glauber dynamics differs significantly from that of the other two dynamics for the chosen parameters. However, we observe that in both the ordered and disordered phases, and across the critical region, the constrained Glauber dynamics accurately reproduce the average magnetization obtained from Langevin dynamics.

S7. CHASE-AND-RUN DYNAMICS FOR NONRECIPROCAL XY SPINS

In Sec. S4, we provided a general derivation of the equivalence of Langevin and constrained Glauber dynamics based on the Hamiltonian embedding defined in the main text, in capturing both nonequilibrium steady states and nonstationary states of nonreciprocal systems. Whereas we presented numerical evidence for this equivalence in the case of a nonequilibrium steady state for vision-cone XY spins in the main text, we now turn our attention to a model whose long-time dynamics exhibit persistent oscillations.

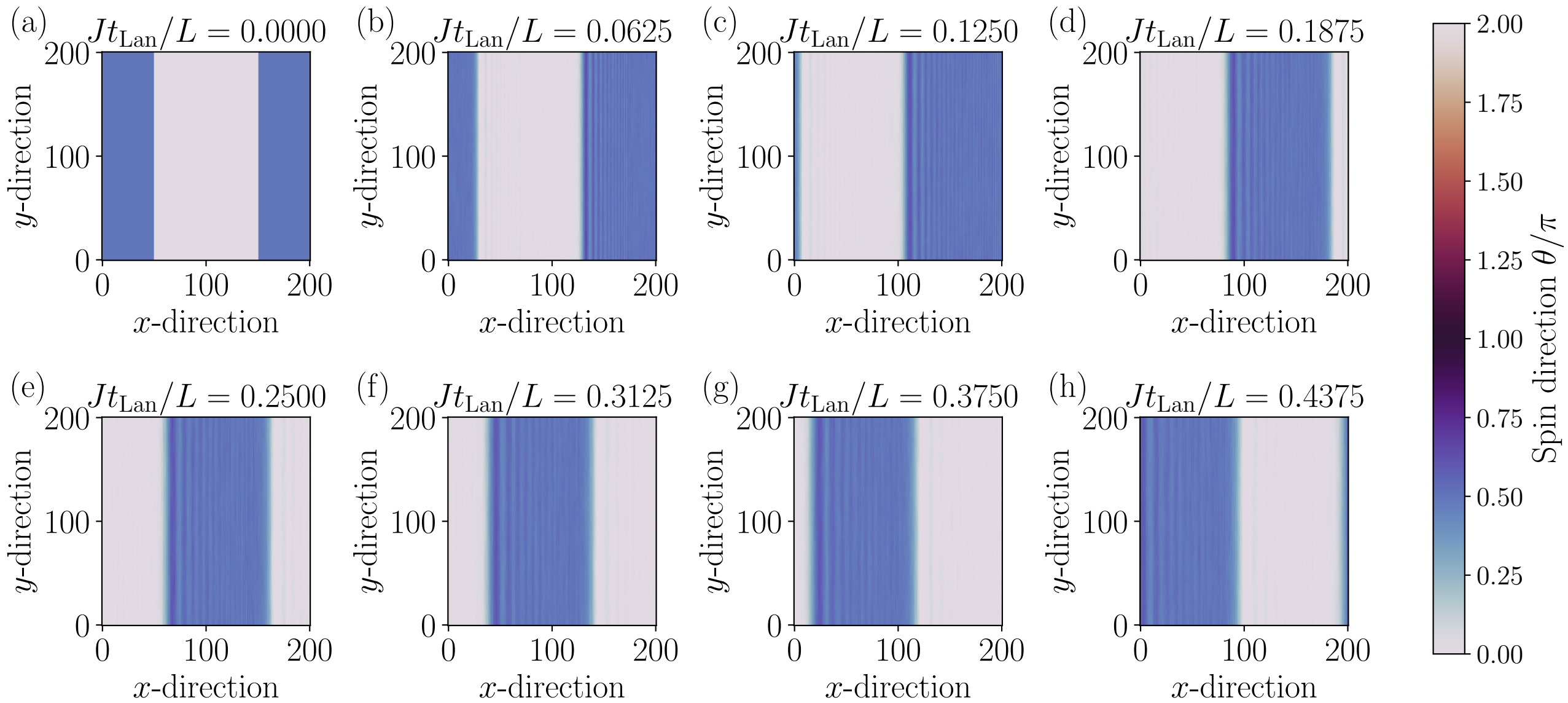

FIG. S5. Snapshots of the spin configuration of nonreciprocal XY spins on a square lattice exhibiting chase-and-run dynamics, generated by solving the Langevin equation, Eq. (S.173). Colors encode the spin angle $\theta_{x,y}$. Starting from a stripe initial condition, the pattern briefly relaxes into a stable shape and then propagates leftward at approximately a constant speed. Parameters are set as $J_{\rightarrow} = J$, $J_{\leftarrow}/J = -0.99$, $J_{\text{rec}}/J = 10$, $T/J = 0.08$; the linear dimension is $L = 200$. The Langevin time-step is $\delta t J = 0.01$.

In this section, we consider nonreciprocal XY spins $\theta_{x,y}$ on a square $L \times L$ lattice which feature persistent chase-and-run dynamics. The corresponding Langevin equation of motion reads as

$$\dot{\theta}_{x,y} = -J_{\rightarrow}\sin\big(\theta_{x,y} - \theta_{x+1,y}\big) - J_{\leftarrow}\sin\big(\theta_{x,y} - \theta_{x-1,y}\big) - J_{\text{rec}}\big[\sin\big(\theta_{x,y} - \theta_{x,y+1}\big) + \sin\big(\theta_{x,y} - \theta_{x,y-1}\big)\big] + \sqrt{2T}\eta_{x,y}(t). \quad \text{(S.173)}$$

Explicitly, the model has constant reciprocal interactions in the y-direction of strength J_{rec}, and constant *nonreciprocal* interactions in the x-direction of strength $J_{\rightarrow}$ (with the spin to the right) and $J_{\leftarrow}$ (with the spin to the left). Whereas the reciprocal interactions along the y-direction can be derived from the potential energy $-J_{\text{rec}}\cos\big(\theta_{x,y} - \theta_{x,y+1}\big)$, there is no potential energy that captures the nonreciprocal interactions along the x-direction for $J_{\rightarrow} \neq J_{\leftarrow}$ since the nonreciprocal force is nonconservative. The spins are coupled to an external reservoir at temperature T and, as before, we set the damping factor to unity. We consider periodic boundary conditions.

To understand the underlying physics, consider first only two spins at two neighboring positions 1 and 2 in the x-direction interacting via strengths $J_{\rightarrow}$ and $J_{\leftarrow}$, respectively, and ignore the other spins. When their coupling strengths satisfy the antisymmetric relation $J_{\rightarrow} = -J_{\leftarrow} = J$, where $J > 0$, the two spins exhibit chase-and-run phase dynamics: spin 1 tends to align with 2 while spin 2 tends to anti-align with spin 1. As a consequence, they will both rotate at a constant speed, maintaining a constant angle difference. These time-dependent chiral dynamics do not have an equilibrium analogue. In the full 2D nonreciprocal model described above, this chiral state can be stable at finite temperature, as a consequence of a subtle interplay between noise and many-body effects [31]. In the following, we regard J as the unit of energy.

We consider an initial state defined by

$$\theta_{x,y} = \begin{cases} 0, & \text{for } x \in (L/4, 3L/4) \\ \pi/2, & \text{else,} \end{cases} \quad \text{(S.174)}$$

It represents a stripe pattern along the y-axis, where spins in the central region point in the positive x-direction, while those in the outer region (connected by the periodic boundary condition) point in the positive y-direction. Snapshots of the initial dynamics until time $Jt_{\text{Lan}} \approx 0.44L$ are presented in Fig. S5 [we label the time variable by t_{Lan} to explicitly denote that this is the time associated with the solution of the Langevin equation]. This configuration exhibits chase-and-run dynamics characteristic of the model. We find that, during the transient dynamics, the configuration reshapes slightly and the pattern then moves to the left at a constant speed v. In Sec. S7 A, we show that the speed remains constant as the linear dimension L is increased, while the period T scales linearly with L since $T = L/v$; therefore, we use the rescaled time Jt_{Lan}/L to eliminate the trivial scaling effect in the dynamical snapshots.

To characterize this oscillatory dynamics, we consider an ensemble of N trajectories, each initialized in the same initial state.

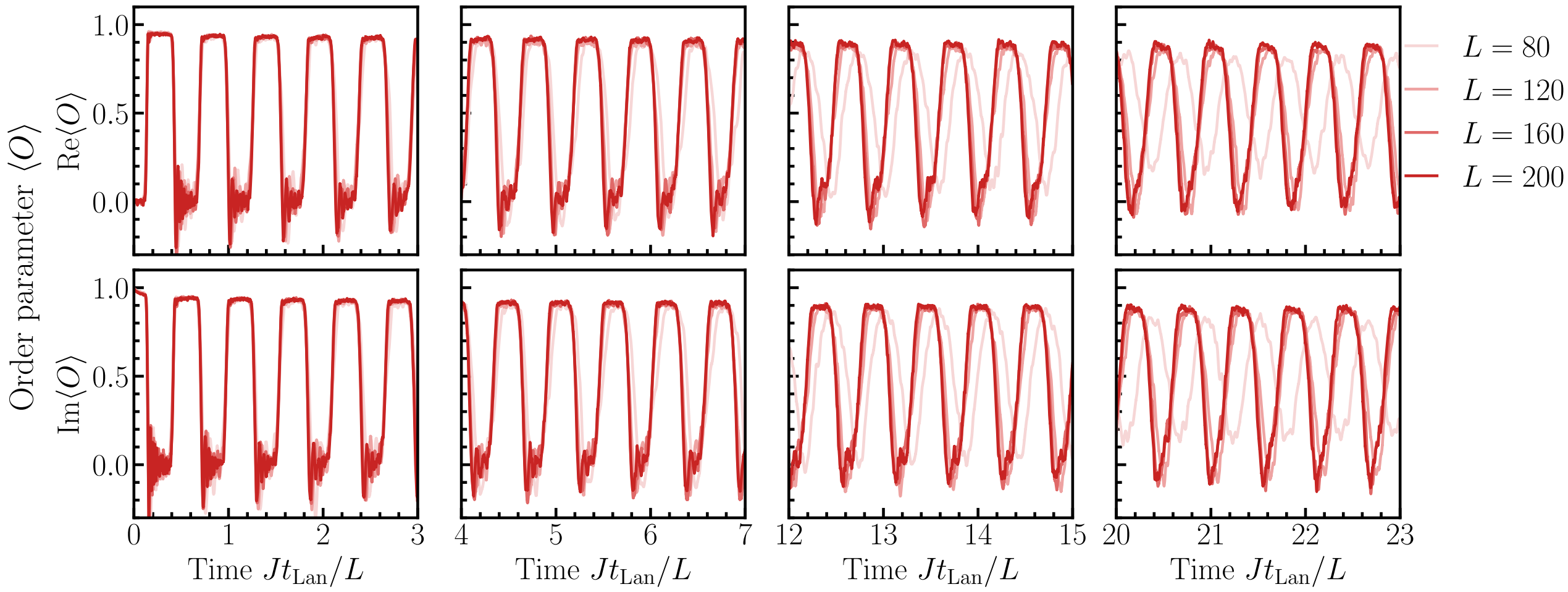

FIG. S6. System size scaling of the oscillatory pattern obtained from Langevin dynamics. The real and imaginary parts of the order parameter $\langle O(t) \rangle$ for the nonequilibrium chiral state are plotted against time for various system sizes L. The color intensity increases as the system size grows from $L = 80$ to $L = 200$ (see legend). The oscillation period exhibits a linear scaling with the system size L: when the time is rescaled by the system size, $Jt_{\rm Lan}/L$, the oscillation curves nearly overlap for different system sizes. The order-parameter amplitude decay decreases as the system size increases, indicating stable oscillatory dynamics in the thermodynamic limit. The parameters are $J_{\rightarrow}/J = 1$, $J_{\leftarrow}/J = -0.99$, $J_{\rm rec}/J = 10$, $T/J = 0.08$; the number of trajectories in the ensemble is $\mathcal{N} = 100$. We use Heun's method with time step $J\delta t_{\rm Lan} = 0.01$ [see text].

We introduce the order parameter [31]

$$\langle O(t) \rangle = \frac{1}{\mathcal{N}} \sum_{\ell=1}^{\mathcal{N}} \frac{1}{L} \sum_{y=1}^{L} e^{i\theta_{1,y}^{(\ell)}(t)}, \tag{S.175}$$

where ℓ labels the trajectory index and $x = 1$ indexes spins in the first column of the square lattice. This order parameter characterizes the average orientation of the spins in the first column. The real and imaginary parts of $\langle O(t) \rangle$ exhibit temporal oscillations that reflect the collective rotation of the stripe pattern.

During the evolution, the pattern of spins propagates collectively at a constant velocity, thereby breaking continuous time-translation symmetry. Thus, the system never relaxes to a steady state, and the state exhibits perpetual oscillatory motion. Hence, it represents a perfect testbed for the Glauber Monte-Carlo dynamics defined with the help of our Hamiltonian embedding.

In the following, we first investigate the scaling effect of this oscillatory dynamics with respect to the system size L. We also compare the Langevin dynamics to constrained Glauber dynamics. To avoid confusion in the comparison, we use $t_{\rm Lan}$ to represent the time in Langevin dynamics and $t_{\rm MC}$ to denote the Monte-Carlo time in the constrained Glauber dynamics. The analytical derivation of the equivalence between the two methods is presented in Sec. S4.

A. Langevin dynamics

To enhance the numerical accuracy of the Langevin simulations, we adopt Heun's method [32]. This method provides second-order accuracy in the time step, i.e., $O(\delta t^2)$, which significantly reduces numerical errors compared to the Euler method. The integration algorithm reads as

$$\theta_{x,y}(t+\delta t) = \theta_{x,y}(t) + \frac{1}{2}[F_{x,y}(\{\theta_{x,y}(t)\}) + F_{x,y}(\{\tilde{\theta}_{x,y}(t+\delta t)\})]\delta t + \sqrt{2T\delta t}\,\eta_{x,y}(t), \tag{S.176}$$

$$\tilde{\theta}_{x,y}(t+\delta t) = \theta_{x,y}(t) + F_{x,y}(\{\theta_{x,y}(t)\})\delta t + \sqrt{2T\delta t}\,\eta_{x,y}(t), \tag{S.177}$$

where $F_{x,y}(\{\theta_{x,y}(t)\})$ denotes the force acting on spin $\theta_{x,y}$ at time t, as defined in Eq. (S.173).

The update procedure proceeds as follows: at each step t, we first calculate the force $F_{x,y}(\{\theta_{x,y}(t)\})$ for all spins. We then propose the provisional state $\tilde{\theta}_{x,y}(t+\delta t)$ using Eq. (S.177) above. Next, we calculate the force $F_i(\{\tilde{\theta}_{x,y}(t+\delta t)\})$ based on this provisional state. Finally, we update the spin configuration $\theta_{x,y}(t+\delta t)$ according to Eq. (S.176). The stochastic force $\eta_{x,y}(t)$ is the same in Eq. (S.176) and Eq. (S.177) in one update step.

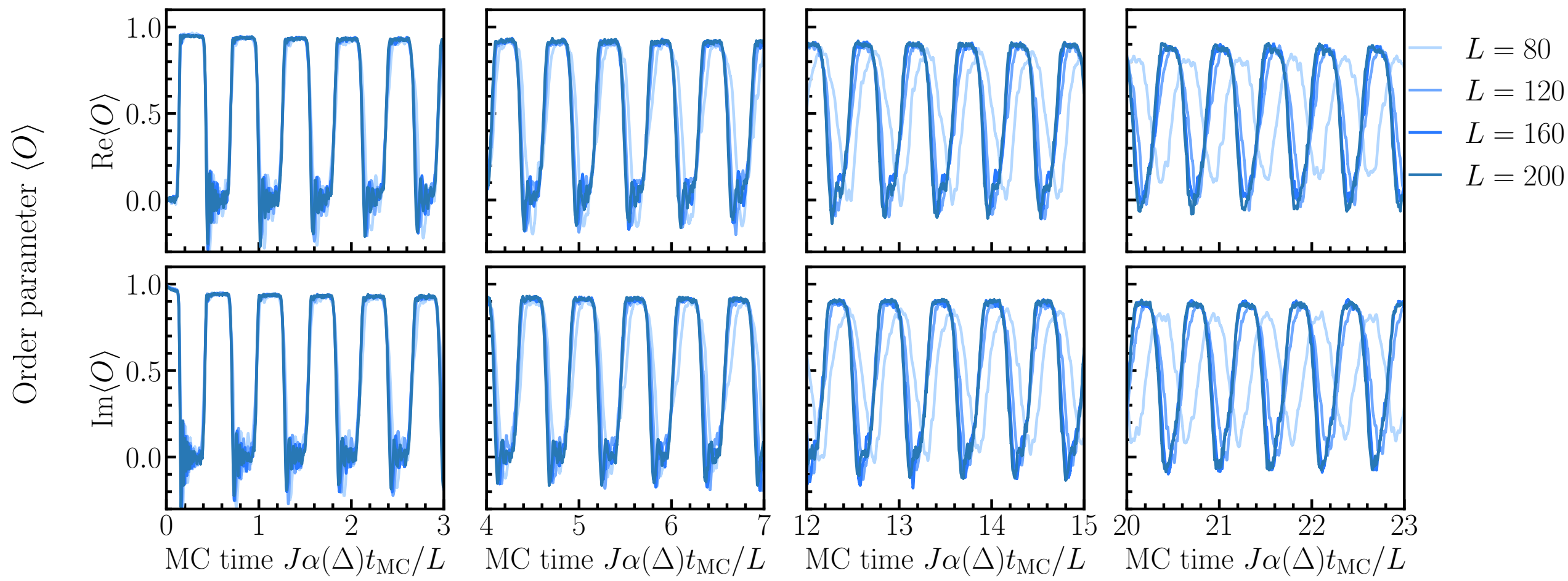

FIG. S7. System size scaling of the oscillatory pattern obtained from constrained Glauber dynamics. The real and imaginary parts of the order parameter $\langle O(t)\rangle$ are plotted as functions of the rescaled Monte-Carlo time $J\alpha(\Delta)t_{\mathrm{MC}}/L$ for various system sizes L. The scaling behavior of constrained Glauber dynamics is in excellent agreement with the Langevin dynamics, see Fig. S9. The time rescaling factor $\alpha(\Delta) \approx 2.37\times 10^{-3}/J$ is determined by matching the first oscillation period of $\langle O(t)\rangle$ from the constrained Glauber dynamics to the same period in the Langevin dynamics for $L = 200$. [cf. Fig. S6]. The parameters are $J_{\rightarrow}/J = 1$ $J_{\leftarrow}/J = -0.99$, $J_{\mathrm{rec}}/J = 10$, $T/J = 0.08$, $\Delta = 0.05$, and the number of trajectories is $\mathcal{N} = 100$.

Let us now examine the scaling behavior of the Langevin dynamics with respect to system size. Figure S6 shows the real and imaginary parts of $\langle O(t)\rangle$ (red curves). We show the initial dynamics, the transients, and the late-time dynamics, respectively. The L-rescaled time axis confirms that the oscillation period scales linearly with the system size L, indicating that the propagation velocity of the stripe pattern remains constant.

To analyze the scaling behavior of the oscillation amplitude more clearly, we plot the results for different system sizes. For the smallest system size $L = 80$ (lightest color), the oscillation amplitude decays noticeably over time. However, as the system size increases, the amplitude stabilizes when plotted against the rescaled time Jt_{Lan}/L, demonstrating that the oscillatory chiral state is stabilized in the thermodynamic limit, underscoring the importance of interactions. Note that the oscillation period also stabilizes as the system size increases.

It is important to emphasize that the stability of the oscillations imposes strict requirements on numerical accuracy. When employing the Euler method for integrating the stochastic differential equations with a time step of $J\delta t_{\mathrm{Lan}} = 0.01$, we observe a decay in the oscillation amplitude, even for system sizes as large as $L = 300$. Eventually, this numerical imprecision would lead to a complete loss of the oscillatory dynamics [not shown]. This effect is, however, avoided here by using the second-order Heun's method.

B. Constrained Glauber dynamics

The Hamiltonian embedding for the nonreciprocal XY spins from Eq. (S.173) reads as

$$
\begin{aligned}
H_{\mathrm{tot}} &= H_{SS} + H_{Sa} - H_{aa},\\
H_{SS} &= -\sum_{x,y} (J_{\rightarrow} + J_{\leftarrow})\left[\cos\left(\theta_{x,y} - \theta_{x+1,y}\right) + \cos\left(\theta_{x,y} - \theta_{x-1,y}\right)\right] + J_{\mathrm{rec}}\left[\cos\left(\theta_{x,y} - \theta_{x,y+1}\right) + \cos\left(\theta_{x,y} - \theta_{x,y-1}\right)\right],\\
H_{Sa} &= -\sum_{x,y} J_{\leftarrow}\cos\left(\theta_{x,y} - \varphi_{x+1,y}\right) + J_{\rightarrow}\cos\left(\theta_{x,y} - \varphi_{x-1,y}\right),\\
H_{aa} &= -\sum_{x,y} J_{\mathrm{rec}}\left[\cos\left(\varphi_{x,y} - \varphi_{x,y+1}\right) + \cos\left(\varphi_{x,y} - \varphi_{x,y-1}\right)\right].
\end{aligned}
\tag{S.178}
$$

We implement constrained Glauber dynamics in which the auxiliary spins $\varphi_{x,y}$ are eliminated by imposing the constraint $\theta_{x,y} - \varphi_{x,y} = \pi$, see Sec. S4 B 3. Consequently, the Glauber algorithm evolves only the original spins $\theta_{x,y}$ and follows the procedure described in Sec. S6 B. The corresponding energy difference ΔE is given in Eq. (**??**).

For the numerical simulation, we use the same initial state as the Langevin dynamics. We also consider the ensembles with the same number of trajectories. For the uniform distribution we sample the Glauber updates from, we choose the semi-width

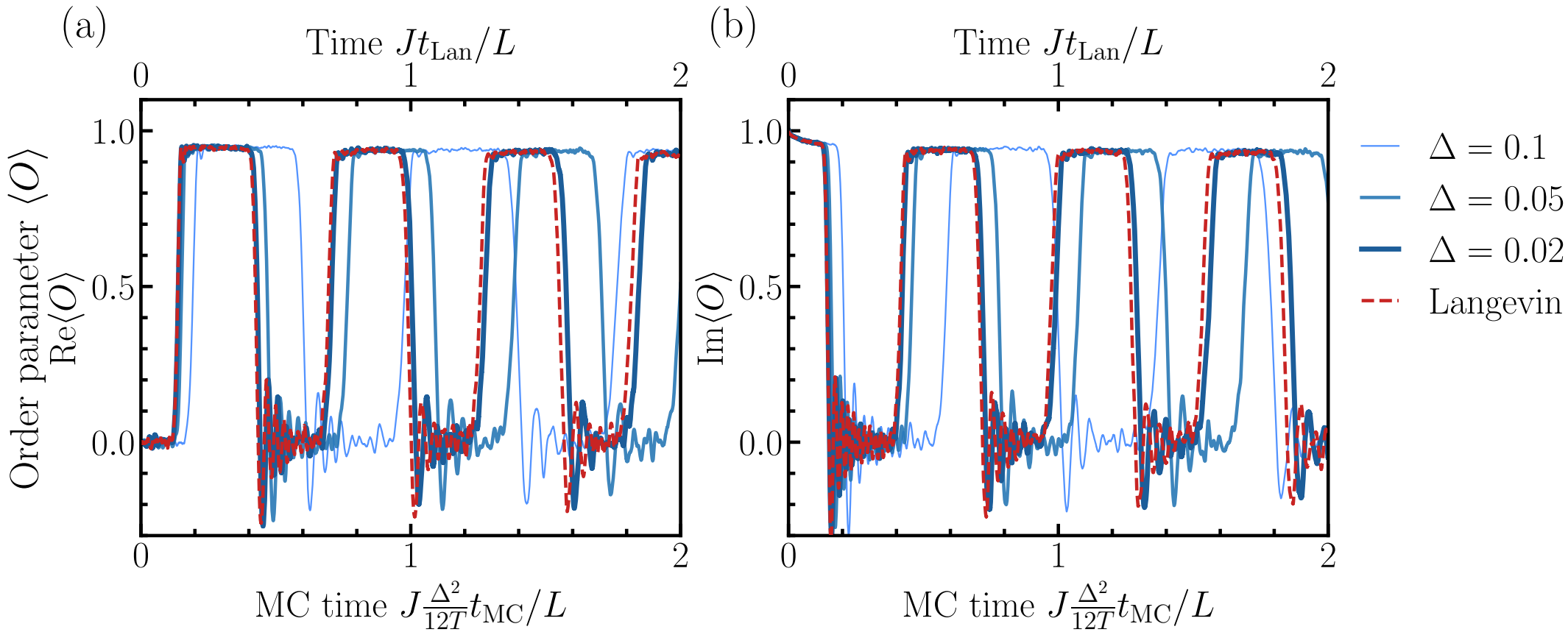

FIG. S8. Comparison between physical Langevin time and rescaled Monte-Carlo time for different semi-widths Δ [see legend]. We use the analytical rescaling factor $\alpha(\Delta) = \Delta^2/(12T)$ to match the Langevin (top x-axis) and Monte-Carlo (bottom x-axis) times [see text]. The difference between the constrained Glauber and the Langevin order-parameter curves decreases in the first three oscillations as the semi-width Δ decreases. The parameters are $J_{\rightarrow} = J$, $J_{\leftarrow}/J = -0.99$, $J_{\rm rec}/J = 10$, $T/J = 0.08$, and the number of trajectories is $\mathcal{N} = 100$. The linear system dimension is $L = 200$.

$\Delta = 0.05$ to reduce the difference between the constrained Glauber dynamics and Langevin dynamics: we have proven in Sec. S4 that the difference between the two vanishes in the limit $\Delta \to 0$.

1. *Monte-Carlo time vs. physical time*

As discussed in Sec. S4 B 2, we can define a (dimensionless) Monte-Carlo time as $t_{\rm MC} = n/L^2$, where n is the iteration step. This MC time is then proportional to the Langevin time (which is the original proper time of the dynamics) through $t_{\rm Lan} = \alpha(\Delta)t_{\rm MC}$. Leading-order perturbation theory in Δ predicts $\alpha(\Delta) = \Delta^2/(12T) \approx 2.60 \times 10^{-3}/J$, cf. Eq. (S.156).

In practice, we find that a slightly better agreement between Langevin and Monte-Carlo simulations is obtained by matching the oscillation period of the order parameter $\langle O(t)\rangle$ from the constrained Glauber dynamics to that of Langevin dynamics for the linear dimension $L = 200$, leading to $\alpha(\Delta) \approx 2.37 \times 10^{-3}/J$. The main discrepancy between the numerically fitted $\alpha(\Delta)$ and the analytical prediction arises from the limited validity of the small-Δ expansion, which assumes $\Delta \ll T/J$. Since in our simulations the temperature strength, $T/J = 0.08$, is comparable to the semi-width $\Delta = 0.05$, truncating the analysis in Sec. S4 B 2 at second order becomes inaccurate. In practice, this leads primarily to a mismatch between the oscillation period extracted numerically and that predicted by the theory.

Figure S8 compares the short-time oscillations obtained from Heun-integrated (physical) Langevin dynamics and constrained Glauber dynamics, using the analytical rescaling factor $\alpha(\Delta) = \Delta^2/(12T)$. As Δ decreases, the order parameter curves agree increasingly well over the first few periods, in line with the above analysis. Note that, formally taking $\Delta \to 0$ requires an infinite number of Monte-Carlo steps to reach finite physical times, making the simulations costly; developing more efficient Hamiltonian-embedding schemes is an interesting direction for future work.

2. *Order-parameter dynamics*

The dynamics of the real part and imaginary part of order parameter $\langle O(t)\rangle$ from the constrained Glauber dynamics are shown in Fig. S7 using blue curves. We find that the oscillation amplitude stabilizes as the system size increases, consistent with the results of Langevin dynamics. The oscillation period also exhibits linear scaling with the system size, in agreement with Langevin dynamics.

We also compare numerically the oscillatory dynamics produced by the Langevin and the constrained Glauber approaches. For a direct comparison, we plot the order parameter $\langle O(t)\rangle$ side by side in Fig. S9. This confirms the validity of the analytical derivation from Sec. S4 B 2.

Finally, we note that constrained Glauber dynamics can reproduce the correct long-time dynamics at time steps (e.g., $J\delta t_{\rm Lan} = 0.01$) where Euler-integration of the corresponding Langevin dynamics exhibits inaccurate amplitude damping of the order parameter oscillations. This indicates that numerical artifacts caused by integrating the Langevin dynamics can lead to potential inaccuracies when determining the phase diagram. By contrast, we find that periodic oscillations remain undamped in the

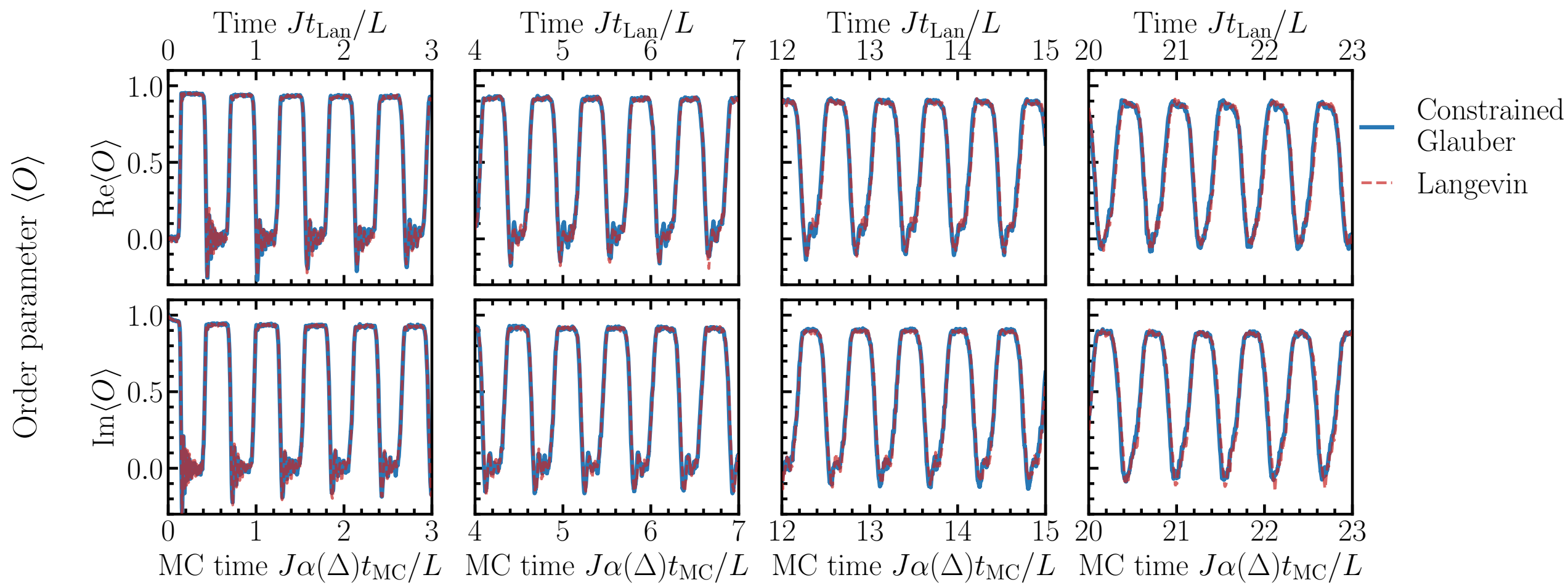

FIG. S9. Comparison of time evolution obtained from Langevin and constrained Glauber dynamics. The real and imaginary parts of the order parameter $\langle O(t)\rangle$ from constrained Glauber dynamics (blue curves) are plotted as functions of the rescaled Monte-Carlo time $J\alpha(\Delta)t_{\rm MC}/L$ [bottom x-axis], while those from Langevin dynamics (blue and green curves) are plotted as functions of Langevin time $Jt_{\rm Lan}/L$ [top x-axis]. The order-parameter curves coincide to an excellent precision, in agreement with the theory analysis in Sec. S4 B 2. The parameters are $J_{\rightarrow} = J$, $J_{\leftarrow}/J = -0.99$, $J_{\rm rec}/J = 10$, $T/J = 0.08$, and the number of trajectories is $\mathcal{N} = 100$. The linear system dimension is $L = 200$. We use $\Delta = 0.05$ for the constrained Glauber dynamics, which is sufficiently small to ensure good agreement with Langevin dynamics.

constrained Glauber dynamics even at relatively coarse angle updates (e.g., $\Delta = 0.3$) [not shown], highlighting one potential advantage of the Hamiltonian embedding method. Thus, for capturing nonequilibrium properties of some nonreciprocal systems, constrained Glauber dynamics can be more robust and efficient than direct Langevin integration.

S8. ENTROPY PRODUCTION IN THE HAMILTONIAN EMBEDDING

In this section, we discuss entropy production from the perspective of the Hamiltonian embedding. Let us denote by $w(\sigma \to \sigma')$ the transition rate from state σ to state σ' and by $p(\sigma)$ the probability of being in state σ. Then, the total entropy production rate $\dot{\Sigma}$ is defined as

$$\begin{aligned}
\dot{\Sigma} &= \dot{S}_{\rm sys} + \dot{S}_{\text{sys-env}}, \\
\dot{S}_{\rm sys} &= \frac{1}{2}\sum_{\sigma,\sigma'} \mathcal{J}(\sigma\to\sigma') \log\frac{p(\sigma)}{p(\sigma')} = \sum_{\sigma,\sigma'} \mathcal{J}(\sigma\to\sigma') \log p(\sigma) = -\sum_{\sigma,\sigma'} \dot{p}(\sigma)\log p(\sigma), \\
\dot{S}_{\text{sys-env}} &= \frac{1}{2}\sum_{\sigma,\sigma'} \mathcal{J}(\sigma\to\sigma') \log\frac{w(\sigma\to\sigma')}{w(\sigma'\to\sigma)},
\end{aligned} \tag{S.179}$$

with the probability current $\mathcal{J}(\sigma \to \sigma') = p(\sigma)w(\sigma \to \sigma') - p(\sigma')w(\sigma' \to \sigma)$. In a nonequilibrium steady state, $\dot{p}(\sigma) = -\sum_{\sigma'} \mathcal{J}(\sigma \to \sigma') = 0$, and hence $\dot{S}_{\rm sys} = 0$ (even though the configuration space currents $\mathcal{J}(\sigma \to \sigma')$ need not vanish). Thus, we only need to consider the entropy flow between the system and its environment $\dot{S}_{\text{sys-env}}$.

For XY variables, using the Glauber expression for the rates, we have $\log(w(\theta \to \theta')/w(\theta' \to \theta)) = \beta\Delta E(\theta, \theta')$, and hence

$$\dot{S}_{\text{sys-env}} = \frac{\beta}{2}\int_0^{2\pi} \mathrm{d}\theta\mathrm{d}\theta'\ \mathcal{J}(\theta\to\theta')\ \Delta E(\theta,\theta'). \tag{S.180}$$

In the nonreciprocal case, ΔE cannot be derived from the gradient of a Hamiltonian — a direct manifestation of the violation of detailed balance. Instead, it can be calculated from the embedding Hamiltonian

$$\Delta E(\theta,\theta') = \frac{1}{2}\left\{H(\theta',\varphi) - H(\theta,\varphi')\right\}\Big|_{\rm constr.}\ . \tag{S.181}$$

We can then write the entropy flow between the system and its environment as

$$\dot{S}_{\text{sys-env}} = \frac{\beta}{4}\int_0^{2\pi} \mathrm{d}\theta\mathrm{d}\theta'\ \mathcal{J}(\theta\to\theta')\ \left\{H(\theta',\varphi) - H(\theta,\varphi')\right\}\Big|_{\rm constr.}, \tag{S.182}$$

where, as stated, one first evaluates the expression in the curly bracket and only then applies the constraint to eliminate the φ and φ' degrees of freedom.

We now write down the same expression for the system-environment entropy flow of a "conventional" Hamiltonian system on an enlarged phase space (θ, φ) (and without a constraint):

$$\dot{S}_{\text{sys-env}} = \frac{\beta}{2} \int_0^{2\pi} \mathrm{d}\theta \mathrm{d}\theta' \mathrm{d}\varphi \mathrm{d}\varphi' \; \mathcal{J}(\theta, \varphi \to \theta', \varphi') \left\{ H(\theta', \varphi') - H(\theta, \varphi) \right\}. \tag{S.183}$$

Compared to Eq. (S.182), in Eq. (S.183) there are three essential and one minor differences:

(i) The energy difference in Eq. (S.183) differs from that in Eq. (S.182) (cf. the position of the primed variables).

(ii) Eq. (S.182) contains in addition the constraint which reduces the number of degrees of freedom (rather than having integrals over all $\theta, \theta', \varphi, \varphi'$ as in Eq. (S.183)). This is an effective change in the phase-space measure; it ensures that the entropy flow is measured by the correct measure on the configuration space of the original θ-degree of freedom.

(iii) The probability current in Eq. (S.183) differs from that in Eq. (S.182); in particular, when one considers a "conventional" Hamiltonian system without a constraint and coupled to a thermal bath, detailed balance stipulates that $\mathcal{J}(\theta, \varphi \to \theta', \varphi') = 0$. This need not be the case for a system with a constraint: $\mathcal{J}(\theta \to \theta') \neq 0$ (and, in fact, it is not, for nonreciprocal systems).

(iv) There is an additional factor of $1/2$ in Eq. (S.182); it ensures that the reciprocal limit is reproduced correctly (see SM, Sec. S4 B 3 where we derive the expression in Eq. (S.181)).

Ultimately, the entropy production is caused by the non-vanishing probability currents $\mathcal{J}(\theta \to \theta')$ in the original degrees of freedom on the constraint manifold. Therefore, it is the application of the constraint that breaks time-reversal symmetry for the original degrees of freedom starting from the Hamiltonian dynamics of the extended, unconstrained system.

---

[1] H. Bateman, On dissipative systems and related variational principles, Phys. Rev. **38**, 815 (1931).
[2] A. Poncet and D. Bartolo, When soft crystals defy newton's third law: Nonreciprocal mechanics and dislocation motility, Physical Review Letters **128**, 048002 (2022).
[3] S. Guillet, A. Poncet, M. Le Blay, W. T. Irvine, V. Vitelli, and D. Bartolo, Melting of nonreciprocal solids: How dislocations propel and fission in flowing crystals, Proceedings of the National Academy of Sciences **122**, e2412993122 (2025).
[4] J. Yan, M. Han, J. Zhang, C. Xu, E. Luijten, and S. Granick, Reconfiguring active particles by electrostatic imbalance, Nature Mater **15**, 1095 (2016).
[5] J. Zhang, R. Alert, J. Yan, N. S. Wingreen, and S. Granick, Active phase separation by turning towards regions of higher density, Nat. Phys. **17**, 961 (2021).
[6] S. Das, M. Ciarchi, Z. Zhou, J. Yan, J. Zhang, and R. Alert, Flocking by turning away, Phys. Rev. X **14**, 031008 (2024).
[7] B. Szabo, G. Szöllösi, B. Gönci, Z. Jurányi, D. Selmeczi, and T. Vicsek, Phase transition in the collective migration of tissue cells: experiment and model, Phys. Rev. E **74**, 061908 (2006).
[8] J. Deseigne, O. Dauchot, and H. Chaté, Collective motion of vibrated polar disks, Phys. Rev. Lett. **105**, 098001 (2010).
[9] P. Baconnier, D. Shohat, C. H. López, C. Coulais, V. Démery, G. Düring, and O. Dauchot, Selective and collective actuation in active solids, Nat. Phys. **18**, 1234 (2022).
[10] P. Baconnier, O. Dauchot, V. Démery, G. Düring, S. Henkes, C. Huepe, and A. Shee, Self-aligning polar active matter, Rev. Mod. Phys. **97**, 015007 (2025).
[11] S. Henkes, Y. Fily, and M. C. Marchetti, Active jamming: Self-propelled soft particles at high density, Phys. Rev. E **84**, 040301 (2011).
[12] M. Brandenbourger, X. Locsin, E. Lerner, and C. Coulais, Non-reciprocal robotic metamaterials, Nat. Commun. **10**, 4608 (2019).
[13] A. Ghatak, M. Brandenbourger, J. Van Wezel, and C. Coulais, Observation of non-hermitian topology and its bulk–edge correspondence in an active mechanical metamaterial, Proc. Natl. Acad. Sci. **117**, 29561 (2020).
[14] N. Okuma, K. Kawabata, K. Shiozaki, and M. Sato, Topological origin of non-hermitian skin effects, Phys. Rev. Lett. **124**, 086801 (2020).
[15] S. Yao and Z. Wang, Edge states and topological invariants of non-hermitian systems, Phys. Rev. Lett. **121**, 086803 (2018).
[16] T. Bäuerle, A. Fischer, T. Speck, and C. Bechinger, Self-organization of active particles by quorum sensing rules, Nat. Commun. **9**, 3232 (2018).
[17] F. A. Lavergne, H. Wendehenne, T. Bäuerle, and C. Bechinger, Group formation and cohesion of active particles with visual perception–dependent motility, Science **364**, 70 (2019).
[18] D. Tong, Lectures on the quantum hall effect, arXiv preprint arXiv:1606.06687 (2016).
[19] C. R. Galley, Classical mechanics of nonconservative systems, Phys. Rev. Lett. **110**, 174301 (2013).
[20] M. Berry and P. Shukla, Hamiltonian curl forces, Proc. R. Soc. A **471**, 20150002 (2015).
[21] D. Landau and K. Binder, *A guide to Monte Carlo simulations in statistical physics* (Cambridge university press, 2021).
[22] H. Risken, *The Fokker-Planck Equation: Methods of Solution and Applications*, second edition, 3rd printing ed., Springer Series in Synergetics No. 18 (Springer, Berlin Heidelberg, 1996).

[23] K. Sekimoto, Langevin equation and thermodynamics, Prog. Theor. Phys. Suppl. **130**, 17 (1998).

[24] S. A. Loos and S. H. Klapp, Irreversibility, heat and information flows induced by non-reciprocal interactions, New Journal of Physics **22**, 123051 (2020).

[25] O. F. Syljuåsen and A. W. Sandvik, Quantum monte carlo with directed loops, Phys. Rev. E **66**, 046701 (2002).

[26] Y. Avni, M. Fruchart, D. Martin, D. Seara, and V. Vitelli, Nonreciprocal ising model, Phys. Rev. Lett. **134**, 117103 (2025).

[27] S. A. Loos, S. H. Klapp, and T. Martynec, Long-range order and directional defect propagation in the nonreciprocal xy model with vision cone interactions, Phys. Rev. Lett. **130**, 198301 (2023).

[28] K. Blom, U. Thiele, and A. Godec, Local order controls the onset of oscillations in the nonreciprocal ising model, Phys. Rev. E **111**, 024207 (2025).

[29] Y. Rouzaire, D. J. G. Pearce, I. Pagonabarraga, and D. Levis, Nonreciprocal interactions reshape topological defect annihilation, Phys. Rev. Lett. **134**, 167101 (2025).

[30] G. Bandini, D. Venturelli, S. A. Loos, A. Jelic, and A. Gambassi, The xy model with vision cone: non-reciprocal vs. reciprocal interactions, arXiv preprint arXiv:2412.19297 (2024).

[31] M. Fruchart, R. Hanai, P. B. Littlewood, and V. Vitelli, Non-reciprocal phase transitions, Nature **592**, 363 (2021).

[32] W. Rüemelin, Numerical treatment of stochastic differential equations, SIAM Journal on Numerical Analysis **19**, 604 (1982).
