# Supplementary material for: Hamiltonian description of non-reciprocal interactions
Source: Nat Phys. 2026 Jun 12;22(8):1350–9. doi: 10.1038/s41567-026-03317-0 (PMC13461368; doi:10.1038/s41567-026-03317-0)
Supplement: Supplementary file 2 [file 41567_2026_3317_MOESM2_ESM.pdf]

nature portfolio

Peer Review File

Hamiltonian description of nonreciprocal interactions

Corresponding Author: Dr Marin Bukov

Version 0:

Reviewer comments:

Reviewer #1

(Remarks to the Author)
In their article "General Hamiltonian description of nonreciprocal interactions", Shi et al. present a novel theoretical method that allows to describe systems with nonreciprocal interactions by viewing them as part of a hypothetical extended system that then has a Hamiltonian structure. This method is discussed both on a theoretical level and with a plethora of case studies.

The results are very impressive and very well presented. I believe they will be impactful enought to justify publication in "Nature Physics". On the one hand, as the authors demonstrate at length, it provides novel ways of analyzing the collective dynamics of systems with nonreciprocal interactions, a topic currently of quite some interest. On the other hand, as the authors point out, it also allows one to engineer novel systems with nonreciprocal interactions by imposing constraints within Hamiltonian dynamics.

Here are my comments:

(a) The distinction between sedimenting particles and particles with non-reciprocal interactions in Fig. 1 is a bit misleading insofar as sedimenting particles are in fact a paradigmatic example for a system that has nonreciprocal interactions. In this case, they arise from hydrodynamic interactions and, importantly, are velocity-dependent.
It would be worth discussing this case explicitly - it's perhapst one of the most commonly occuring forms of nonreciprocal interactions, and I'm not sure it's covered by what the authors do. Specifically, it's assumed in the proof in the SI that the forces are position-dependent and can be derived from an interaction potential (even though that may not be the same for F_ij and F_ji), that's a pretty restrictive assumption that should not be sold as "most general case".

(b) A key aspect of the presented method is the initial constraint (4) that is preserved by the time evolution and ensures the desired behavior of the system. This reminds me a bit of the ADM formalism in general relativity, also here an initial condition has to be imposed that is then preserved during the time evolution and that then gives a Hamiltonian time evolution. Is there a relation?

(c) The result is in several ways counterintuitive, which is part of what makes it interesting, but also implies the need for particularly careful justification. For instance, a key difference between Hamiltonian and active (or non-reciprocal) systems is that the former do, if not externally driven, approach a stationary probability measure where the probability for a certain configuration is proportional to exp(-\beta H), which in particular implies that there are no configuration space currents in steady state. Such currents are in general present in systems with nonreciprocal interactions. If now systems with nonreciprocal interactions can be considered part of a larger Hamiltonian system, I would expect that this entire Hamiltonian system has no configuration space currents, but why is it then that a subsystem of that system can have them?

(d) Relatedly, systems with nonreciprocal interactions generally have a non-zero entropy production in steady state which Hamiltonian systems (if not externally driven) do not have. Thus, I would expect that, e.g., the system consisting of original plus auxiliary layer has zero entropy production in steady state. Why is it then that a subsystem (original layer only) has nonzero entropy production? I see that the authors briefly discuss the contrast between symplectic and dissipative dynamics and explain it via the fact that the dissipative dynamics contains a subset of measure zero - that's fine, but I'm not sure it captures the entire problem. Specifically, nonzero entropy production is about breaking time-reversal symmetry, and the problem to be solved would be how a subsystem breaks time-reversal symmetry if the entire system has it.

(e) The first sentence of the abstract sounds as if the authors develop a new active matter modeling technique. In general there can be active matter systems that do not have nonreciprocal interactions and systems with nonreciprocal interactions that are not active (see point a), so it's worth distinguishing these systems.

(f) From a purely computational point of view, in which cases is the described procedure advantageous compared to standard ones and in which cases is it not? Introducing auxiliary degrees of freedom also means that effectively you need to simulate a larger system, which increases the computational effort.

(g) The authors point out that their Hamiltonian "does not describe the energy of the original nonreciprocal system, but merely provides a generator for its dynamics". Does it provide the energy of the total (fictitious) system?

Michael te Vrugt
Johannes Gutenberg-Universität Mainz

Reviewer #3

(Remarks to the Author)
The manuscript of Shi et al. addresses an important theoretical question in the field of nonreciprocal condensed matter. In nonreciprocal systems, the interacting degrees of freedom are coupled asymmetrically, in stark contrast to conventional Hamiltonian systems where the resulting interaction forces fulfil Newton's third law and are, thus, by definition, reciprocal. Nonreciprocal systems have recently gained tremendous interest in various fields, from macroscopic biological systems such as bird flocks and predator-prey systems, programmed robots, to chemically activated colloids and non-hermitian quantum-optical systems. A large body of previous research has focused on the collective behavior of nonreciprocal systems, particularly the appearance of spontaneous time-symmetry breaking and exceptional points. Other activities include, e.g., the investigation of the impact of nonreciprocal interacting on assembly dynamics, as well as the stochastic thermodynamics of nonreciprocal systems (that are, per se, out of equilibrium). However, a crucial question is how to actually handle nonreciprocal interactions when it comes to numerical simulations, particularly Monte Carlo simulation that rely on a conventional (i.e., Hamiltonian) energy function. More generally, one may ask how to set up a general statistical-mechanical framework of nonreciprocal systems given the fact that there is no Hamiltonian.

Here the authors propose an elegant way to overcome these problems by an embedding strategy. To this end they construct a Hamiltonian that involves, in addition to the original variables, a set auxiliary variables whose dynamics is fixed by certain constraints. All couplings in this new Hamiltonian are reciprocal. The constraints are chosen such that the new Hamiltonian produces the original nonreciprocal equations of motion. A major advantage of this technique is that the reciprocal Hamiltonian allows to use standard Monte Carlo algorithms that are well established in statistical physics, such as the Glauber algorithm. Indeed, the authors show (numerically and based on plausible arguments) that, with the new Hamiltonian, an MC simulation involving Glauber rates with the new Hamiltonian yields a phase transition of a nonreciprocal XY model at the same critical temperature as that obtained from Langevin simulations of the original equations of motion. This is indeed an important and novel result, other algorithms based on "selfish" energies yield different critical temperatures. The authors also show that the method can be applied to various other important models of (classical) nonequilibrium nonreciprocal physics, including active matter and colloids under feedback control. Finally, they investigate the uses of periodic driving fields (inspired by Floquet engineering of quantum systems) to tune the nonreciprocal interactions and to generate a dimensional crossover at large frequencies.

I have read the paper with great interest and I think that it represents a significant advancement from a methodological point of view. While the usage of auxiliary variables to treat problems with nonconservative forces is not new, the present extension of this idea to nonreciprocal systems is, and the authors have presented nice and convincing examples how to use this idea in a variety of systems.

However, I am not convinced that the work should be published in Nature Physics. First, it seems that the framework does not produce any new insights from a physical point of view, it rather seems technical. I also do not see that it provides us with a new way of interpreting the physical behavior seen in such nonreciprocal systems.
Second, there seem to be some severe restrictions. Indeed, the examples provided in the manuscript seem to refer to stationary states. Can the method also be used or extended to investigate systems with transient dynamics or even spontaneous time-symmetry breaking, that is one of the major features of nonreciprocal systems? Further, while the authors frequently refer to (hard) condensed matter physics, it is not clear to me whether and how the framework could be extended to quantum systems ?

There are also a number of more minor points that should be addressed.

- the section on "Floquet engineering" is interesting, but to me it falls a bit "from the sky". For example, what is the motivation for writing the periodic drive within the coupling term? What is meant by "dynamical localization" in the present case? What exactly is the connection to the physics of nonreciprocal systems? Maybe the effective Hamiltonian (10) is just needed as a starting point for the Floquet-Magnus expansion??

- as the authors state in the conclusion, embedding methods are also used in other contexts, and they name a few.
Another example worthwhile to be mentioned is Markovian embedding that is routinely used to describe and simulate non-Markovian systems with memory kernels
(See, e.g.,
https://journals.aps.org/pre/abstract/10.1103/PhysRevE.81.011136,https://pubs.rsc.org/en/content/articlelanding/2018/sm/c8sm01817k,
https://iopscience.iop.org/article/10.1088/1742-5468/abdead).

- the authors state that their method could also be helpful in the context of formulating a thermodynamic description of nonreciprocal systems. I would like to mention that several recent works in this direction already exist, see, in particular,
https://journals.aps.org/prresearch/abstract/10.1103/PhysRevResearch.5.L022033,
https://iopscience.iop.org/article/10.1088/1367-2630/abcc1e.

Version 1:

Reviewer comments:

Reviewer #1

(Remarks to the Author)

The authors have substantially revised the manuscript and added a significant number of discussions and calculations that provide useful physical insights. The results constitute, in my view, an important progress in the literature on nonreciprocal interactions. I recommend the article for publication.

Open Access This Peer Review File is licensed under a Creative Commons Attribution 4.0 International License, which permits use, sharing, adaptation, distribution and reproduction in any medium or format, as long as you give appropriate credit to the original author(s) and the source, provide a link to the Creative Commons license, and indicate if changes were made.
In cases where reviewers are anonymous, credit should be given to 'Anonymous Referee' and the source.
The images or other third party material in this Peer Review File are included in the article’s Creative Commons license, unless indicated otherwise in a credit line to the material. If material is not included in the article’s Creative Commons license and your intended use is not permitted by statutory regulation or exceeds the permitted use, you will need to obtain permission directly from the copyright holder.
To view a copy of this license, visit https://creativecommons.org/licenses/by/4.0/

1 Reply to the first reviewer – NPHYS-2025-08-03697/Shi

Reviewer's comment:

In their article "General Hamiltonian description of nonreciprocal interactions", Shi et al. present a novel theoretical method that allows to describe systems with nonreciprocal interactions by viewing them as part of a hypothetical extended system that then has a Hamiltonian structure. This method is discussed both on a theoretical level and with a plethora of case studies.

The results are very impressive and very well presented. I believe they will be impactful enought to justify publication in "Nature Physics". On the one hand, as the authors demonstrate at length, it provides novel ways of analyzing the collective dynamics of systems with nonreciprocal interactions, a topic currently of quite some interest. On the other hand, as the authors point out, it also allows one to engineer novel systems with nonreciprocal interactions by imposing constraints within Hamiltonian dynamics.

Authors' Response:

We thank the Referee for the positive evaluation!

Reviewer's comment:

(a) The distinction between sedimenting particles and particles with non-reciprocal interactions in Fig. 1 is a bit misleading insofar as sedimenting particles are in fact a paradigmatic example for a system that has nonreciprocal interactions. In this case, they arise from hydrodynamic interactions and, importantly, are velocity-dependent. It would be worth discussing this case explicitly - it's perhapst one of the most commonly occuring forms of nonreciprocal interactions, and I'm not sure it's covered by what the authors do. Specifically, it's assumed in the proof in the SI that the forces are position-dependent and can be derived from an interaction potential (even though that may not be the same for F_{ij} and F_{ji}), that's a pretty restrictive assumption that should not be sold as "most general case".

Authors' Response:

We thank the reviewer for bringing up this point. Our schematic of a sedimenting particle in Fig. 1 was meant to represent a non-Hamiltonian system where the driving force emerges from a potential. We did not introduce interactions at that point, since we sketched a single particle. However, we agree that the choice might be confusing given that sedimenting particles are a classic example of non-reciprocal interactions. We have made changes to Fig. 1 to avoid this issue. As an example of a non-Hamiltonian potential system, we now display two interacting particles in contact with a frictional substrate. Moreover, we now explicitly mention the case of non-reciprocal interactions in sedimenting particles both in the abstract and in the introduction.

In addition to mentioning it in the Main Text, we also added a section in the SI (cf. Sec. B6) to show how sedimenting particles can be treated within our framework, at least in a simple case of point particles that have reached their terminal velocity. As the referee can see, despite the presence of velocity-dependent forces, this model falls within the framework we presented in the original manuscript (this has to do with the structure of the equations of motion rather than the underlying physics).

Prompted by the comment by the referee, we have also addressed the generality of our theory w.r.t. generic velocity-dependent interactions, such as Lorentz-like interaction forces. In the new Sec A2 in the SM, we now explicitly construct an embedding for velocity-dependent nonreciprocal interactions. Thus, the embedding structure applies in general.

Finally, we have also amended our title to drop the word "General". We believe that this helps to avoid the impression that our Hamiltonian framework is completely general (there may still exist other types of nonreciprocal interactions which we have not considered – e.g., acceleration-dependent forces – and which are not yet captured by the current constructions; we leave these to future work).

Referee's comment:

(b) A key aspect of the presented method is the initial constraint (4) that is preserved by the time evolution and ensures the desired behavior of the system. This reminds me a bit of the ADM formalism in general relativity, also here an initial condition has to be imposed that is then preserved during the time evolution and that then gives a Hamiltonian time evolution. Is there a relation?

Authors' Response:

The Referee's intuition is spot-on: there is a strong similarity between ADM and our construction, which arises from an underlying gauge structure, defined precisely by an initial constraint being preserved by the dynamics. Besides ADM, a very similar structure occurs also in Chern-Simons theory, which describes the quantum Hall effect.

In a nutshell, one can show that the local (on-site) constraint in our embedding is first-class (according to Dirac's classification), which elevates the corresponding Lagrange multiplier to a gauge parameter. The

corresponding gauge transformation shows that the gauge group is SO(2). For Heisenberg spins (rather than XY-spins) the gauge group can even be non-Abelian.

While we have made some progress in understanding the mathematical structure of our embedding as a gauge theory, our understanding of the physical meaning and its consequences for nonreciprocal interactions is not mature enough for us to comment on this interesting feature in the manuscript. We will, therefore, continue to analyze this exciting aspect in a follow-up study.

Referee's comment:

(c) The result is in several ways counterintuitive, which is part of what makes it interesting, but also implies the need for particularly careful justification. For instance, a key difference between Hamiltonian and active (or non-reciprocal) systems is that the former do, if not externally driven, approach a stationary probability measure where the probability for a certain configuration is proportional to $\exp(-\beta H)$, which in particular implies that there are no configuration space currents in steady state. Such currents are in general present in systems with nonreciprocal interactions. If now systems with nonreciprocal interactions can be considered part of a larger Hamiltonian system, I would expect that this entire Hamiltonian system has no configuration space currents, but why is it then that a subsystem of that system can have them?

Authors' Response:

We thank the Referee for this insightful question. To answer it, note first that the constraint manifold and the rest of the embedding phase space are not coupled by the dynamics: if we start on the constraint manifold, trajectories do not leave it (this is the preservation of the constraint); vice versa, if we start outside the constraint manifold, trajectories never cross it. Therefore:

- on the constraint manifold, the dynamics are nonreciprocal and the initial distribution may evolve into a long-time state with configuration-space currents.
- outside the constraint manifold, the dynamics are reciprocal; if, in addition, they are also ergodic, any initial distribution will evolve into a thermal distribution outside the constraint manifold.

If we now consider the long-time behavior of a generic distribution on the total embedding space, it may support a part on the constraint manifold that develops currents, and a part outside the constraint manifold that will thermalize. However, since the constraint manifold is a set of measure zero within the total embedding phase space, its physics does not contribute to expectation values of observables supported on the total phase space. However, when observables are explicitly restricted to the constraint manifold, the distribution exhibits the expected nonreciprocal behavior (which may include configuration space currents, or continuous time-translation symmetry breaking), because the way we sample the entire embedding phase space is not "thermodynamic".

We included a new paragraph in Sec III of the manuscript to explain this in greater detail.

Referee's comment:

(d) Relatedly, systems with nonreciprocal interactions generally have a non-zero entropy production in steady state which Hamiltonian systems (if not externally driven) do not have. Thus, I would expect that, e.g., the system consisting of original plus auxiliary layer has zero entropy production in steady state. Why is it then that a subsystem (original layer only) has nonzero entropy production? I see that the authors briefly discuss the contrast between symplectic and dissipative dynamics and explain it via the fact that the dissipative dynamics contains a subset of measure zero - that's fine, but I'm not sure it captures the entire problem. Specifically, nonzero entropy production is about breaking time-reversal symmetry, and the problem to be solved would be how a subsystem breaks time-reversal symmetry if the entire system has it.

Authors' Response:

Let us denote by $w(\sigma \to \sigma')$ the transition rate from state σ to state σ' and by $p(\sigma)$ the probability of being in state σ, the total entropy production rate reads as

$$\begin{aligned}
\dot{\Sigma} &= \dot{S}_{\text{sys}} + \dot{S}_{\text{sys-env}}, \\
\dot{S}_{\text{sys}} &= \frac{1}{2}\sum_{\sigma,\sigma'} j(\sigma \to \sigma') \log \frac{p(\sigma)}{p(\sigma')} = \sum_{\sigma,\sigma'} j(\sigma \to \sigma') \log p(\sigma) = -\sum_{\sigma,\sigma'} \dot{p}(\sigma) \log p(\sigma), \\
\dot{S}_{\text{sys-env}} &= \frac{1}{2}\sum_{\sigma,\sigma'} j(\sigma \to \sigma') \log \frac{w(\sigma \to \sigma')}{w(\sigma' \to \sigma)},
\end{aligned} \tag{1}$$

with the probability current $j(\sigma \to \sigma') = p(\sigma)w(\sigma \to \sigma') - p(\sigma')w(\sigma' \to \sigma)$. In a nonequilibrium steady state, $\dot{p}(\sigma) = -\sum_{\sigma'} j(\sigma \to \sigma') = 0$, and hence $\dot{S}_{\text{sys}} = 0$ (even though the configuration space currents $j(\sigma \to \sigma')$ need not vanish). Thus, we only need to consider the entropy flow between the system and its environment $\dot{S}_{\text{sys-env}}$.

For XY variables, using the Glauber expression for the rates, we have $\log(w(\theta \to \theta')/w(\theta' \to \theta)) = \beta\Delta E(\theta, \theta')$, and hence

$$\dot{S}_{\text{sys-env}} = \frac{\beta}{2}\int_0^{2\pi} \mathrm{d}\theta\mathrm{d}\theta'\ j(\theta \to \theta')\ \Delta E(\theta, \theta'). \tag{2}$$

In the nonreciprocal case, ΔE cannot be derived from the gradient of a Hamiltonian – a direct manifestation of the violation of detailed balance. Instead, as we now emphasize in the revised version of the manuscript, it can be calculated from the embedding Hamiltonian

$$\Delta E(\theta, \theta') = \frac{1}{2}\{H(\theta', \varphi) - H(\theta, \varphi')\}\big|_{\text{constr.}}\ . \tag{3}$$

We can then write the entropy flow between the system and its environment as

$$\dot{S}_{\text{sys-env}} = \frac{\beta}{4}\int_0^{2\pi} \mathrm{d}\theta\mathrm{d}\theta'\ j(\theta \to \theta')\ \{H(\theta', \varphi) - H(\theta, \varphi')\}\big|_{\text{constr.}}, \tag{4}$$

where, as stated, one first evaluates the expression in the curly bracket and only then applies the constraint to eliminate the φ and φ' degrees of freedom.

We now write down the same expression for the system-environment entropy flow of a "conventional" Hamiltonian system on an enlarged phase space (θ, φ) (and without a constraint):

$$\dot{S}_{\text{sys-env}} = \frac{\beta}{2}\int_0^{2\pi} \mathrm{d}\theta\mathrm{d}\theta'\mathrm{d}\varphi\mathrm{d}\varphi'\ j(\theta, \varphi \to \theta', \varphi')\ \{H(\theta', \varphi') - H(\theta, \varphi)\}. \tag{5}$$

Compared to Eq. (4), in Eq. (5) there are three essential and one minor differences:

(i) The energy difference in Eq. (5) differs from that in Eq. (4) (cf. the position of the primed variables).

(ii) Eq. (4) contains in addition the constraint which reduces the number of degrees of freedom (rather than having integrals over all $\theta, \theta', \varphi, \varphi'$ as in Eq. (5)). This is an effective change in the phase-space measure; it ensures that the entropy flow is measured by the correct measure on the configuration space of the original θ-degree of freedom.

(iii) The probability current in Eq. (5) differs from that in Eq. (4); in particular, when one considers a "conventional" Hamiltonian system without a constraint and coupled to a thermal bath, detailed balance stipulates that $j(\theta, \varphi \to \theta', \varphi') = 0$. This need not be the case for a system with a constraint: $j(\theta \to \theta') \neq 0$ (and, in fact, it is not, for nonreciprocal systems).

(iv) There is an additional factor of 1/2 in Eq. (4); it ensures that the reciprocal limit is reproduced correctly (see SM, Sec D2c where we derive the expression in Eq. (3)).

Ultimately, the entropy production is caused by the non-vanishing probability currents $j(\theta \to \theta')$ in the original degrees of freedom on the constraint manifold. Therefore, it is the application of the constraint that breaks time-reversal symmetry for the original degrees of freedom starting from the Hamiltonian dynamics of the extended, unconstrained system. We believe this answers the question raised by the Referee. We added a new Sec H in the SI with the above information.

To better illustrate the time-translation symmetry-breaking behavior of nonreciprocal systems numerically, we have added a new Sec. IV B to the main text, and Sec. G in the SM. In there, we discuss a new model of XY-spins with nonreciprocal interactions, for which the long-time configuration space distribution breaks continuous time-translation symmetry and exhibits a persistent configuration space current. The comparison shows an excellent agreement between the original Langevin dynamics and the corresponding Hamiltonian embedding Glauber dynamics. These results therefore confirm that our framework can capture sustained configuration-space currents typical of non-reciprocal systems as a result of a constraint applied on an otherwise Hamiltonian system.

Referee's comment:

(e) The first sentence of the abstract sounds as if the authors develop a new active matter modeling technique. In general there can be active matter systems that do not have nonreciprocal interactions and systems with nonreciprocal interactions that are not active (see point a), so it's worth distinguishing these systems.

Authors' Response:

We agree with the reviewer. To make our motivation more general, we now make reference to (passive) sedimenting particles instead of active colloids in the first sentence of the abstract. Then, we believe that

the introduction already approaches the subject of non-reciprocal interactions from a general standpoint, not focusing solely on active systems.

Referee's comment:

(f) From a purely computational point of view, in which cases is the described procedure advantageous compared to standard ones and in which cases is it not? Introducing auxiliary degrees of freedom also means that effectively you need to simulate a larger system, which increases the computational effort.

Authors' Response:

The referee is correct to point out that the embedding phase space has twice the number of degrees of freedom (dof), compared to the original system, which appears to render simulations involving the embedding degrees of freedom more expensive.

However, in the case of the Glauber dynamics we propose, this is actually not the case. One can think of the auxiliary dof as a slave variable and eliminate it entirely from the Glauber transition rate dynamics for the original dof. In this way, the corresponding Glauber dynamics has the same number of dof as the original system. Here, the embedding is essential to correctly identify the proper Glauber update rule. To explain how this works, we have added a new subsection in the SM, Sec. D2. We have also re-written Sec. IV of the main text to prevent this confusion, and we added a corresponding new Methods section.

That said, other techniques that build on the embedding may require keeping track of twice the number of degrees of freedom. In this case, we point out that adding the auxiliary degrees of freedom (which effectively doubles the system size) does not change the numerical complexity of any algorithm; it affects just the prefactor of the corresponding scaling law. We therefore conclude that the auxiliary layer will not be prohibitive for large-scale numerical simulations. This is now also mentioned both in the Outlook and in the SM.

Referee's comment:

(g) The authors point out that their Hamiltonian "does not describe the energy of the original nonreciprocal system, but merely provides a generator for its dynamics". Does it provide the energy of the total (fictitious) system?

Authors' Response:

Yes, the embedding Hamiltonian H provides an energy for the total (fictitious) system of auxiliary and original degrees of freedom; moreover, as expected, it is conserved under the total dynamics. However, to the best of our knowledge, we are unaware of any relevance of the dynamics generated by the *unconstrained* H to the nonreciprocal system of interest.

2 Reply to the third reviewer – NPHYS-2025-08-03697/Shi

Reviewer's comment:

The manuscript of Shi et al. addresses an important theoretical question in the field of nonreciprocal condensed matter. In nonreciprocal systems, the interacting degrees of freedom are coupled asymmetrically, in stark contrast to conventional Hamiltonian systems where the resulting interaction forces fulfil Newton's third law and are, thus, by definition, reciprocal. Nonreciprocal systems have recently gained tremendous interest in various fields, from macroscopic biological systems such as bird flocks and predator-prey systems, programmed robots, to chemically activated colloids and non-hermitian quantum-optical systems. A large body of previous research has focused on the collective behavior of nonreciprocal systems, particularly the appearance of spontaneous time-symmetry breaking and exceptional points. Other activities include, e.g., the investigation of the impact of nonreciprocal interactions on assembly dynamics, as well as the stochastic thermodynamics of nonreciprocal systems (that are, per se, out of equilibrium). However, a crucial question is how to actually handle nonreciprocal interactions when it comes to numerical simulations, particularly Monte Carlo simulations that rely on a conventional (i.e., Hamiltonian) energy function. More generally, one may ask how to set up a general statistical-mechanical framework of nonreciprocal systems given the fact that there is no Hamiltonian.

Here the authors propose an elegant way to overcome these problems by an embedding strategy. To this end they construct a Hamiltonian that involves, in addition to the original variables, a set of auxiliary variables whose dynamics is fixed by certain constraints. All couplings in this new Hamiltonian are reciprocal. The constraints are chosen such that the new Hamiltonian produces the original nonreciprocal equations of motion. A major advantage of this technique is that the reciprocal Hamiltonian allows the use of standard Monte Carlo algorithms that are well established in statistical physics, such as the Glauber algorithm. Indeed, the authors show (numerically and based on plausible arguments) that, with the new Hamiltonian, an MC simulation involving Glauber rates with the new Hamiltonian yields a phase transition of a nonreciprocal XY model at

the same critical temperature as that obtained from Langevin simulations of the original equations of motion. This is indeed an important and novel result, other algorithms based on "selfish" energies yield different critical temperatures. The authors also show that the method can be applied to various other important models of (classical) nonequilibrium nonreciprocal physics, including active matter and colloids under feedback control. Finally, they investigate the uses of periodic driving fields (inspired by Floquet engineering of quantum systems) to tune the nonreciprocal interactions and to generate a dimensional crossover at large frequencies.

I have read the paper with great interest and I think that it represents a significant advancement from a methodological point of view. While the usage of auxiliary variables to treat problems with nonconservative forces is not new, the present extension of this idea to nonreciprocal systems is, and the authors have presented nice and convincing examples how to use this idea in a variety of systems.

Authors' Response:

We are grateful to the Referee for the positive evaluation!

Reviewer's comment:

However, I am not convinced that the work should be published in Nature Physics. First, it seems that the framework does not produce any new insights from a physical point of view, it rather seems technical. I also do not see that it provides us with a new way of interpreting the physical behavior seen in such nonreciprocal systems.

Authors' Response: We appreciate the reviewer's point of view. To avoid confusion, we invite the referee to read this part of the answer only after they have read the responses below to the other points they have raised.

We believe that our key advance is not just technical but also yields new physical insights: Our results uncover an underlying symplectic structure for non-reciprocal systems. This unexpected finding provides a new way of thinking about this class of systems — it is a conceptual advance.

As an aside, we note that very concretely, the discussion in response to Referee A's question on entropy production provides an example of physical insights on the role of constraints in Hamiltonian dynamics.

The general physical insight that nonreciprocal systems enjoy a symplectic structure has important implications. For example, the symplectic structure enables the use of canonical transformations, which help identify fast and slow degrees of freedom in nonreciprocal systems. Integrating out the fast degrees of freedom allows one to construct effective theories. Therefore, this insight can pave the way towards developing a statistical mechanics for nonreciprocal systems – something that would be very difficult without an energy function.

In addition, directly resulting from the insight that non-reciprocal systems have a Hamiltonian structure, we present two main technical advances:

1. Our results allow us to perform Monte-Carlo simulations of non-reciprocal systems (also in the case of nonstationary states, see below). This advance settles an important open issue in the field. It will enable researchers to perform Monte-Carlo simulations that capture the original Langevin dynamics, in contrast to previous ad-hoc approaches such as the selfish energy.

2. We use our embedding to apply Hamiltonian engineering techniques. This second advance will enable researchers to control interactions in non-reciprocal systems, which opens the door for experiments to use periodic drives to explore new regimes not accessible otherwise. We give an example of this idea in our manuscript by showing how to implement a dimensional crossover, using a periodic driving to go from a 2D square lattice to a 1D chain of spins, which destroys the order-disorder phase transition in the system.

Beyond these advances, we believe that the real impact of our work is that it opens the door to further discoveries in the field. For example, the ability to perform Monte-Carlo simulations will enable a more detailed investigation of the nature of the order-disorder transition in the vision-cone XY model, which has been strongly debated in the recent literature. Even beyond that, our Hamiltonian could allow classical non-reciprocal systems to be systematically quantized, thus paving the way towards a theory of quantum active matter. These are just two examples of the new research avenues that our work opens. In the revised manuscript, we have highlighted these points in the Discussion to emphasize the potential impact of our work.

On top of this, and perhaps on a more philosophical level, the distinction between a new physical insight and a technical advance is not as clear-cut as it might seem. The former tends to follow, and to feed back on, the latter in indirect and largely unpredictable ways, and a sufficiently deep technical advance almost inevitably involves physical insight. After all, the arguably original manuscript on the Monte Carlo method by Metropolis and Ulam in 1949 was largely devoid of new physical insights in the narrow sense, and its first application only arrived several years later.

We of course do not at all want to equate our manuscripts with the epochal papers from 1949 and 1953, but we nonetheless believe that we have (i) generated a genuine *conceptual* (rather than purely technical) advance,

(ii) implemented this advance on the technical level for the study of a problem family under active consideration, (iii) based on this, identified new avenues to control the cooperative behaviour of matter in a very concrete way and (iv) charted a clear way ahead for further discoveries, as well as hinted at more ambitious long-term goals of such a research programme.

We therefore believe that, in summary, our manuscript deserves the broad dissemination provided by *Nature Physics* precisely because it provides progress on many different levels, demonstrating not just technically how to embed non-reciprocal systems into a Hamiltonian structure, but also opening several research directions in this active and rapidly developing field.

Reviewer's comment:

Second, there seem to be some severe restrictions. Indeed, the examples provided in the manuscript seem to refer to stationary states. Can the method also be used or extended to investigate systems with transient dynamics or even spontaneous time-symmetry breaking, that is one of the major features of nonreciprocal systems?

Authors' Response:

Yes, the Hamiltonian embedding framework also reproduces the correct behavior of nonreciprocal systems whose long-time behavior breaks continuous time-translation symmetry. We added a new Sec IV B to the main text and a new Sec. G in the SM, where we demonstrate this explicitly, discussing both transient dynamics and time-symmetry breaking in a related XY-spin model. We also provide a video with a back-to-back comparison of the Langevin and Glauber dynamics. In addition, we added a new Methods section and a subsection to Sec. D2 in the SM, where we provide a general proof and identify the physical time variable in the Glauber dynamics.

Therefore, our Hamiltonian framework of nonreciprocal systems is generic and does not suffer from the restriction mentioned by the Referee. What is more, we find that our Glauber dynamics is numerically more stable at long times than Langevin dynamics based on off-the-shelf Euler solvers, cf. Fig. S1 below. Full agreement with the constrained Glauber dynamics is then achieved by using Heun's method for the Langevin dynamics. We are grateful to the Reviewer for urging us to add this important improvement to the manuscript. This generality is yet another reason why we expect our results to be useful for a wide range of nonreciprocal systems, and why we believe our idea deserves the broad dissemination provided by *Nature Physics*.

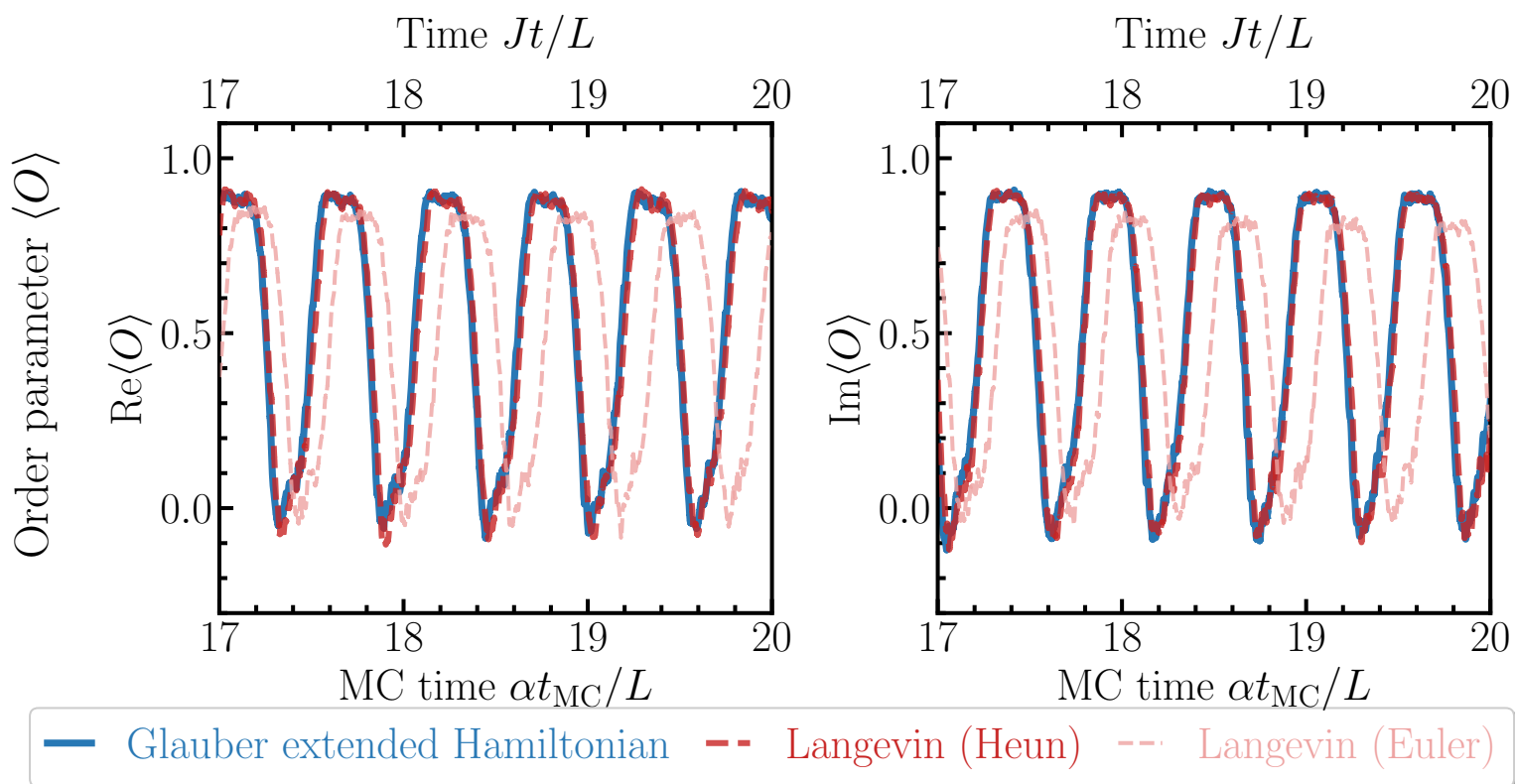

Figure S1: Time evolution of the complex-valued order parameter O for the run-and-chase dynamics of XY spins, averaged over an ensemble of trajectories: real part (left panel) and imaginary part (right panel). A long-time comparison between Langevin dynamics and constrained Glauber dynamics based on the Hamiltonian embedding shows that the latter captures the behavior of nonstationary states to an excellent precision [see revised manuscript and SM]. We find that Langevin dynamics using the Euler-update solver (orange dashed line) exhibits damping in the amplitude of oscillations as a numerical artifact; switching to the more stable Heun-update solver (red dashed line) stabilizes the amplitude. By contrast, we find that the Glauber Monte Carlo dynamics based on the embedding Hamiltonian is numerically stable at long times. The simulation parameters are as in Fig. 4 of the main text.

Further, while the authors frequently refer to (hard) condensed matter physics, it is not clear to me whether and how the framework could be extended to quantum systems ?

Authors' Response:

In the present manuscript, we develop the Hamiltonian framework for classical systems. Due to its symplectic structure (the original θ and auxiliary φ variables are canonically conjugate: $\{\theta_j, \varphi_i\} = \delta_{ij}$), one can imagine leveraging it to *canonically quantize* classical nonreciprocal systems. Note that the existence of conjugate variables is a prerequisite to define the Heisenberg uncertainty relation; hence, canonical quantization of nonreciprocal systems, which lack an intrinsic Poisson bracket structure, cannot be carried out a priori. It is here that we expect the Hamiltonian embedding to provide new physics insights. However, canonical quantization with a hard constraint is a difficult problem, which we are currently working on (with a close relation to quantizing gauge theories, see response to point (b) of Reviewer 1 above).

When it comes to quantum nonreciprocal systems, note first that they do not admit a description within the Schroedinger formalism, which is inherently Hamiltonian. The general consensus in the field seems to be that quantum nonreciprocity is described by nonhermitian Hamiltonians. However, we believe that this classification is too loose since there exist single-particle nonhermitian systems (the analogue of a quantum active particle), whereas the notion of nonreciprocity applies to pairwise interactions and does not have a single-particle limit. Therefore, at the moment, it is unclear to us what the distinctive features of quantum nonreciprocity really are. On a separate note, modern quantum simulators, in contrast to nonreciprocal systems, are inherently Hamiltonian: by providing a Hamiltonian formulation, it could be possible to emulate the quantum physics of nonreciprocal systems in quantum simulators using auxiliary degrees of freedom (but this requires solving the canonical quantization problem described above first). It is precisely these kinds of questions that we hope the canonical quantization, enabled by our framework, will help us answer.

We revised the usage of the word *quantum* in the manuscript to explicitly make it clear that we mean canonical quantization.

Reviewer's comment:

- the section on "Floquet engineering" is interesting, but to me it falls a bit "from the sky".

Authors' Response:

We thank the reviewer for raising this point. In the revised manuscript, we have revised this section to better introduce how Floquet engineering is an interesting application that makes direct use of the Hamiltonian embedding. We have also better explained the potential of this technique to control non-reciprocal interactions in experiments. Our arguments are as follows:

One of the features of our Hamiltonian embedding is its symplectic (phase-space) structure. Since this is not made use of by the Glauber MC dynamics, we decided to provide a second application that makes explicit use of it.

In general, a symplectic structure allows us to use canonical transformations to analyze the physics of the underlying system. For instance, this can be used to identify strongly and weakly interacting degrees of freedom, conserved quantities, etc. This is also intimately related to the existence of a Hamiltonian as a generator of dynamics.

To provide a useful example of what the embedding enables us to do, we consider a hypothetical experiment where it is not possible to independently control the horizontal and vertical interaction strengths of the nonreciprocal system of interest (Fig. 5a). This is common, since interactions are typically hard-coded by the microscopic details of physical systems. Clearly, such a control would enhance the set of phenomena that can be studied in the lab.

We therefore decided to showcase how one can achieve independent control of the couplings in the x- and y-directions on the lattice using what is called Floquet engineering – the application of high-frequency periodic drives to the system. Floquet theory uses the Hamiltonian formalism to derive an effective Hamiltonian whose equations of motion describe the stroboscopic dynamics. This is where our embedding becomes essential, since nonreciprocal systems (without the embedding) do not have a Hamiltonian.

We revised the manuscript accordingly.

Reviewer's comment:

- For example, what is the motivation for writing the periodic drive within the coupling term? What is meant by "dynamical localization" in the present case? What exactly is the connection to the physics of nonreciprocal systems? Maybe the effective Hamiltonian (10) is just needed as a starting point for the Floquet-Magnus expansion??

Authors' Response:

What is the motivation for writing the periodic drive within the coupling term?

The motivation comes from (hard) condensed matter and specifically the physics of Josephson-Junction arrays (see Ref. [60]), where an external AC magnetic field is modeled by a vector potential that couples to the interaction between the phase of the superconducting order parameter (the XY degree of freedom). In our

model with nonreciprocal interactions, we consider a drive that couples in this way, since clearly, if one assumes no control over the interaction strength, one cannot modulate J_{ij} itself in time; hence, we use a coupling to an external field, motivated by the above setup.

What is meant by "dynamical localization" in the present case?

A suppression of the interaction strength between XY spins along the x-direction. The term originates in the quantum literature. We decided to move this comment and the corresponding explanation to a footnote, since it is not essential to the discussion.

What exactly is the connection to the physics of nonreciprocal systems?

Periodic driving allows one to use Hamiltonian engineering to acquire improved control over model parameters; in turn, this allows one to access wider parameter regions in the phase diagram of nonreciprocal systems and study dimensional crossover regimes. We have clarified this point in the revised manuscript.

Maybe the effective Hamiltonian (10) is just needed as a starting point for the Floquet-Magnus expansion?

It could be considered the first term in a series of higher-order terms in the Floquet-Magnus expansion to be computed. The key contribution of our work is to enable the use of the Hamiltonian formalism in nonreciprocal systems in the first place (with the Floquet Hamiltonian providing a concrete example). The symplectic structure of our Hamiltonian embedding, however, is much more general, and we expect it to enable the transfer of other ideas and techniques and lead to new physical insights for nonreciprocal systems.

In the revised manuscript, we made explicit modifications to clarify all these questions.

Reviewer's comment:

- as the authors state in the conclusion, embedding methods are also used in other contexts, and they name a few. Another example worthwhile to be mentioned is Markovian embedding that is routinely used to describe and simulate non-Markovian systems with memory kernels (See, e.g.,
https://journals.aps.org/pre/abstract/10.1103/PhysRevE.81.011136,
https://pubs.rsc.org/en/content/articlelanding/2018/sm/c8sm01817k,
https://iopscience.iop.org/article/10.1088/1742-5468/abdead).

Authors' Response:

We thank the referee for bringing up this point. We added a sentence in the conclusion where we refer to the above papers.

Reviewer's comment:

- the authors state that their method could also be helpful in the context of formulating a thermodynamic description of nonreciprocal systems. I would like to mention that several recent works in this direction already exist, see, in particular, https://journals.aps.org/prresearch/abstract/10.1103/PhysRevResearch.5.L022033,
https://iopscience.iop.org/article/10.1088/1367-2630/abcc1e.

Authors' Response:

We appreciate the point raised. We now explicitly refer to these articles in the conclusion of the manuscript.
